# Cobalt Complex-Directed Self-Assembly of a Polyoxometalate-Based Species: Influence of Synthetic Methods on the Structure and Properties of Hybrid Assemblies

**DOI:** 10.1021/acsomega.5c00186

**Published:** 2025-04-17

**Authors:** Dino Kuzman, Mario Pajić, Lucija Drempetić, Josipa Sarjanović, Jana Pisk, Tomica Hrenar, Marina Cindrić, Višnja Vrdoljak

**Affiliations:** † Faculty of Science, Department of Chemistry, University of Zagreb, Horvatovac 102a, Zagreb 10000, Croatia; ‡ Laboratory of Phisical Chemistry, Rudjer Bošković Institute, Bijenička cesta 54, Zagreb 10000, Croatia

## Abstract

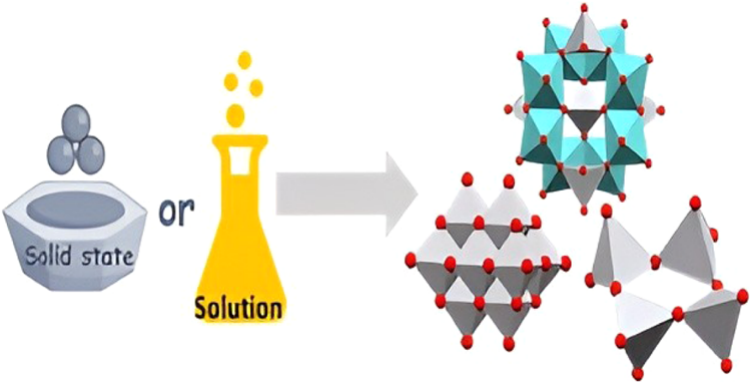

A study of the influence of a counterion, mono- and dicarboxylic acid, the addition of the MoO_4_^2–^ anion, and synthetic routes on the formation of polyoxo(molybdo)vanadates was presented. Using four Co^III^(ammine) complex cations in acidic aqueous solutions of VO_3_^–^, 10 crystalline solids with seven different oxovanadate anions were isolated: [H_2_V_10_O_28_]^4–^ (in **1** and **5**), [V_10_O_28_]^6–^ (in **2**, **6** and **7**), [H_3_V_10_O_28_Na(H_2_O)_2_]*_n_*^*n*–^ (in **3**), [H_2_V_10_O_28_Na(H_2_O)_8_]^3–^ (in **4**), [V_4_O_12_]^4–^ (in **8**), [V_2_O_6_Na(H_2_O)]*_n_*^*n*–^ (in **9**), and [V_3_O_9_]_n_^3–^ (in **10**), as well as two molybdovanadate anions [H_2_Mo_8_V_5_O_40_Na_2_(H_2_O)_8_]^2–^ (in **11**) and [HMo_2_V_7_O_27_]^6–^ (**12**). The oxovanadate species found in solids mostly correspond with the predominant species present in solution at a specific pH. Mechanochemically accelerated vapor-assisted aging and transformation from the amorphous precipitate to the crystalline products was demonstrated to be the efficient method for isolating the intermediates. Thus, in the reaction involving [Co(C_2_O_4_)(NH_3_)_4_]^+^, ammonium vanadate, sodium molybdate, and any carboxylic acid, the protonated decavanadate anions [H_2_V_10_O_28_]^4–^ and [H_2_V_10_O_28_Na(H_2_O)_8_]^2–^ transform to [H_3_V_10_NaO_28_(H_2_O)_2_]*_n_*^*n*–^ and [H_2_Mo_8_V_5_O_40_]^5–^. All of the products were characterized in the solid state via single-crystal X-ray diffraction, infrared spectroscopy, and thermogravimetric and elemental analyses. The oxovanadates **1**, **2**, **5**, **10**, and molybdovanadate **11** were also examined as catalysts for the oxidation of benzyl alcohol. The results of catalytic reactions showed that polyoxometalates as catalysts exhibit good selectivity but limited activity. In addition, for four decavanadates, **1**, **2**, **5**, and **6**, the electrostatic potential was mapped on the calculated electron isodensity surfaces. The reaction profiles for their synthesis were investigated in detail using quantum chemical calculation.

## Introduction

Polyoxometalates (POMs) are predominantly anionic inorganic clusters and important intermediates in the reaction pathway from water-soluble metal ions to insoluble metal oxides. Their isolation enables an explanation and control over reaction pathways. POMs exhibit remarkable structural diversity and potential applications in many research areas, such as catalysis,^1−3^ medicinal chemistry,^4^ and materials science.^5−7^ An important part of the POMs family, polyoxovanadates, POVs, have attracted increasing attention due to their structural diversity and electronic properties,^8^ as well as their versatile applications in industry^9,10^ and materials science.^11^ POVs contain highly symmetrical core assemblies of VO_n_ units, such as [V_3_O_9_]^3–^,^12^ [HV_4_O_12_]^3–^,^13^ and [V_5_O_14_]^3–^,^14^ with tetrahedrally coordinated V^V^ ions, or [V_10_O_28_]^6–^,^15^ [V_12_O_32_]^4–^,^16^ [V_13_O_34_]^3–^,^17^ [V_15_O_42_]^9–^,^18^ [V_16_O_42_]^4–^, [V_18_O_42_]^12–^, and [V_34_O_82_]^10–^,^19,20^ with octahedrally coordinated V^V^ ions. These VO_*n*_ units can form either discrete molecular clusters or link together to form one-dimensional chains, two-dimensional layers, or three-dimensional frameworks. When paired with alkali or alkaline earth cations, the electrostatic interactions are the predominant forces between POVs and cations in the solid state or weakly coordinating solvents. In contrast, complex cations such as [ML_*n*_]^*m+*^ show different interactions with POVs, including hydrogen bonding, ion–dipole, partially covalent bonds, van der Waals interactions, and cation−π interactions. The complex combinations of these interactions can coexist and have a significant impact on the hybrid structure and properties.

It is obvious that counterions play a crucial role in isolating pure-phase POVs, but their role goes far beyond simple charge balance. A widely used synthetic pathway for the preparation of POVs involves an acid condensation reaction, which proceeds through the protonation of smaller POVs.^21−23^ The most thermodynamically stable species obtained through a series of hydrolysis or condensation reactions are metavanadates and decavanadates. Although the pH value of the solution and temperature/pressure are considered the main factors affecting speciation, other parameters, such as the ionic strength of the solution, the presence of chelating or reducing components, and the nature of countercation also need to be carefully considered.^24^ In an aqueous solution of POVs under acidic conditions, two major species, [V_10_^V^O_28_]^6–^ and [VO_2_(H_2_O)_4_]^+^, are present,^25^ which can vary in protonation, i.e. [H*_n_*V_10_O_28_]^(6–*n*)^ with *n* = 1–4.^26,27^

In continuation of our research, we explore the reaction systems containing the [VO_3_]^−^ anion, acetic or succinic acid and, [Co(ox)(NH_3_)_4_]^+^, [Co(NH_3_)_6_]^3+^, [Co(ox)(en)_2_]^+^ or [Co(en)_3_]^3+^ cations (ox^2–^ = C_2_O_4_^2–^ oxalato anion; en = H_2_NCH_2_CH_2_NH_2_, ethylenediamine ligand).

These reactions occur within pH ranges of 4 and 6 and at constant concentrations of reactants. The pH of the solution was adjusted by using acetic or succinic acid. These two acids were used in order to examine their possible coordination to vanadium and consequently their influence the condensation process of V_*x*_O_*y*_ species. The stability of the coordinated species could be a crucial factor in the process of obtaining [V_*x*_O_*y*_]^*n*−^. Our previous investigations showed that the condensation process of [MoO_4_]^2–^ units can be correlated with the coordination properties of oxalic acid and the hydrogen bonding ability of the macrocation used.^28^ By applying solution-based methods and mechanochemically accelerated vapor-assisted aging synthesis, we were able to isolate 10 polyoxovanadates and two molybdovanadates of different nuclarities and compositions. The proposed composition of molybdovanadates, [Co(C_2_O_4_)(NH_3_)_4_]_2_[H_2_Mo_8_V_5_O_40_Na_2_(H_2_O)_8_]·*n*H_2_O (**11**) and Na_3_[Co(en)_3_][HMo_2_V_7_O_27_]·*n*H_2_O (**12**), were previously reported,^29^ and the crystal structure of (**10**) was described in the literature.^30^ However, in this work, they were obtained under different conditions. Additionally, some solid-state reactions promoted by mechanochemically accelerated vapor-assisted aging have enabled us to identify reaction intermediates. For example, in the reaction with the [Co(ox)(NH_3_)_4_]^+^ cation and in the presence of acetic or succinic acid and sodium molybdate, decavanadates transform from **1** and **4** to **3** and **11** after 21 days, respectively.

POVs are suitable molecular models for studying catalytic reactions due to their well-defined structures and adjustable redox properties, which can be fine-tuned by varying the metal center.^31^ To date, catalytic investigations have primarily focused on a limited set of POV clusters including [V_4_O_12_]^4–^, [V_6_O_19_]^8–^, [V_10_O_28_]^6–^, and semimetal-doped [V_18_O_42_]^12–^. For oxidative processes, oxidants such as *tert*-butyl hydroperoxide (TBHP) and hydrogen peroxide (H_2_O_2_) are favored over molecular oxygen, as these peroxides are readily activated by POVs to form reactive vanadium-peroxo intermediates, which are pivotal in enhancing catalytic performance.^32−36^ Recent advancements in the catalytic applications of POVs for the oxidative transformation of organic compounds have garnered significant scientific interest. Furthermore, the incorporation of metal ions or metal complexes into POV systems has been shown to stabilize the negative charge of POV anions while introducing additional active sites. This dual function improves substrate conversion efficiency and increases selectivity toward the desired products. The role of POVs as catalysts in the selective oxidation of alcohols to aldehydes or ketones has not been thoroughly investigated, especially when compared to polyoxomolybdates, polyoxotungestates, and V(V)-substituted polyoxomolybdates, which have been extensively used as catalysts.^34^ In this study, we focused on investigating the catalytic activity of compounds **1**, **2**, **5**, **10**, and **11** in the oxidation of benzyl alcohol, conducting the experiments under more environmentally friendly conditions. The results were further compared to those obtained using the precursors for the synthesis of compounds **1**, **2**, **5**, **10**, and **11**, [Co(ox)(NH_3_)_4_]NO_3_·H_2_O (**I**), [Co(en)_3_]NO_3_·H_2_O (**II**), and [Co(ox)(NH_3_)_4_]Cl·1.5H_2_O (**III**) as well as compounds previously described in the literature [Co(ox)(NH_3_)_4_]_4*n*_[Na_2_Mo_8_O_29_(H_2_O)_4_]_*n*_·6*n*H_2_O (**IV**), [Co(ox)(NH_3_)_4_]_4_[Mo_8_O_26_]·4H_2_O·C_4_H_6_O_4_ (**V**), and [Co(ox)(NH_3_)_4_]_4_[Mo_8_O_26_]·12H_2_O (**VI**).^29^

## Results and Discussion

### Synthetic Procedures

**Figure 1:**
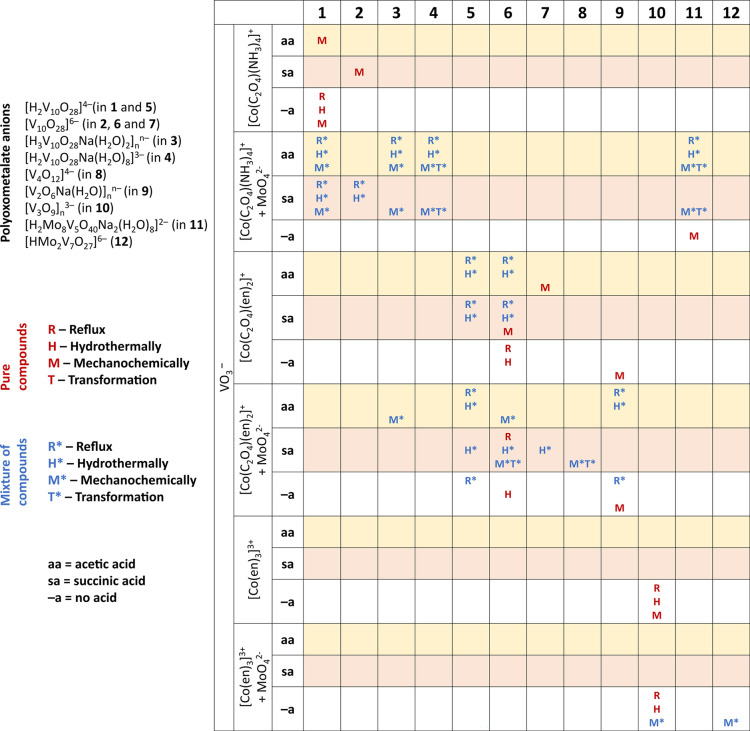
Schematic representation of the reaction conditions and obtained products.

The synthetic methods used to obtain poloxovanadates and molybdovanadates were simple one-pot solution synthesis under reflux or hydrothermal conditions, as well as mechanochemically accelerated vapor-assisted aging synthesis. The isolation of the compounds was dependent on the choice of Co(III) precursor, reaction temperature, and added acid. A list of the polyoxometalate compounds formulas and the conditions under which they were obtained are given in Tables 1 and S1 and Figure 1. Products obtained exclusively as mixtures of two or more crystalline phases were separated mechanically.

**Table 1:** List of Products and Their Formulas

compound	formula
**1**	[Co(ox)(NH_3_)_4_]_4_[H_2_V_10_O_28_]·6H_2_O
**2**	[Co(ox)(NH_3_)_4_]_6_[V_10_O_28_]·16H_2_O
**3**(37)	[Na_2_(H_2_O)_10_]*_n_*[H_3_V_10_O_28_Na(H_2_O)_2_]*_n_*·3*n*H_2_O
**4**	[Co(ox)(NH_3_)_4_]_2_[H_2_V_10_O_28_Na(H_2_O)_8_]·8H_2_O
**5**	[Co(ox)(en)_2_]_4_[H_2_V_10_O_28_]·12H_2_O
**6**	[Co(ox)(en)_2_]_6_[V_10_O_28_]·*n*H_2_O (*n* ≈ 13.5)
**7**	(H_2_en)_2_[Co(ox)(en)_2_]_2_[V_10_O_28_]·*n*H_2_O (*n* ≈ 2)
**8**	[Co(ox)(en)_2_]_4_[V_4_O_12_]·14H_2_O
**9**	[Co(ox)(en)_2_]_n_[V_2_O_6_Na(H_2_O)]*_n_*·*n*H_2_O
**10**(30)	[Co(en)_3_][V_3_O_9_]*_n_*·*n*H_2_O
**11**(29)	[Co(ox)(NH_3_)_4_]_3_[H_2_Mo_8_V_5_O_40_Na_2_(H_2_O)_8_]·5.5H_2_O
**12**(29)	Na_3_[Co(en)_3_][HMo_2_V_7_O_27_]·*n*H_2_O

The pH of the solutions ranged between 4 and 6, where the formation of decavanadate clusters was expected.^38,39^ The main products of the solution-based reactions between the vanadate anion and the tetraammineoxalatocobalt(III), or bis(ethylenediamine)oxalatocobalt(III) cation in the presence of acetic and succinic acid were differently protonated decavanadates, [H_*x*_V_10_O_28_]^(6–*x*)–^, which are the most stable vanadium(V) species under acidic conditions.

Extensive H-bonding frameworks between ions, cations [Co(ox)(NH_3_)_4_]^+^, [Co(ox)(en)_2_]^+^ and H_2_en^+^, and decavanadate anions, build up supramolecular 3D structures and are present in all isolated species: [H_2_V_10_O_28_]^4–^ (in **1** and **5**) and [V_10_O_28_]^6–^ (in **2**, **6**, and **7**).

**Figure 2:**
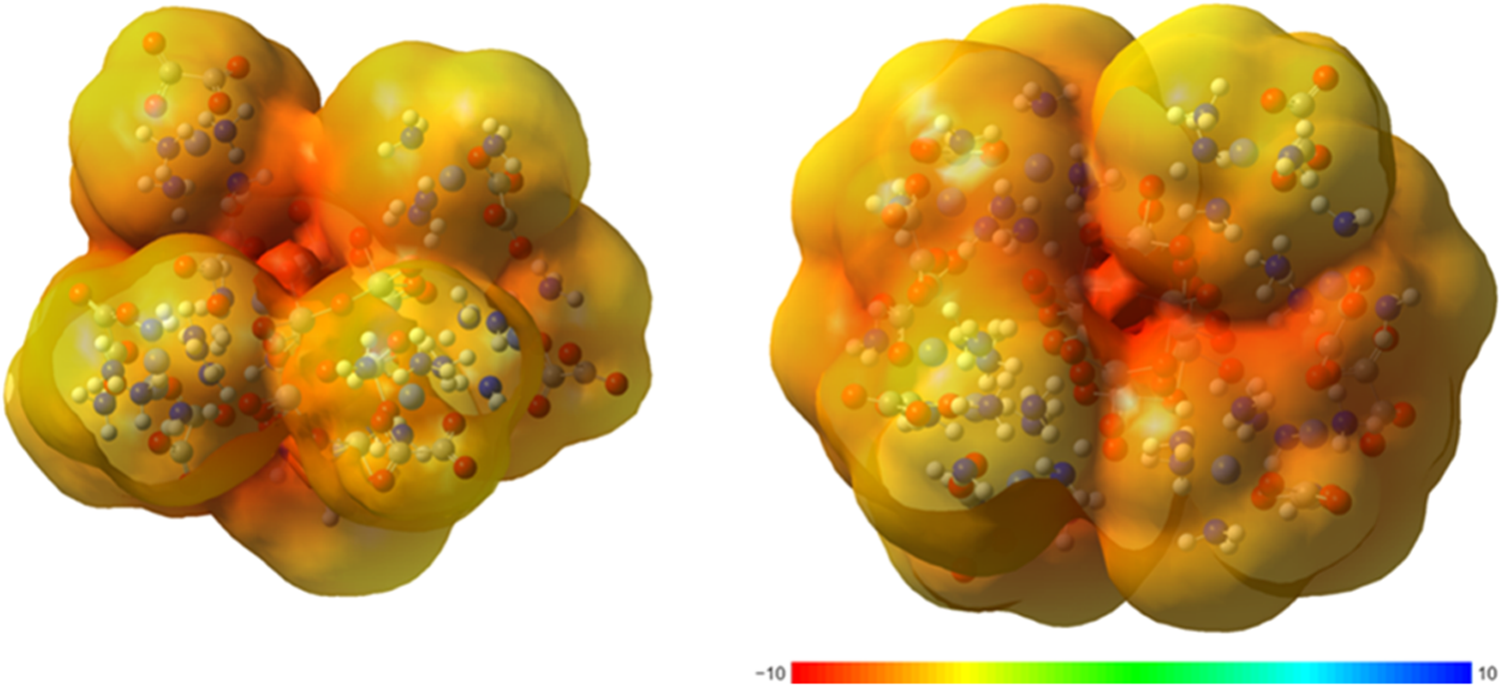
Electrostatic potential mapped on the electron density isosurface (0.001 au) for the optimized structure of **1** and **2**.

Triply and doubly protonated decavanadate anions incorporating sodium cations, [H_3_V_10_O_28_Na(H_2_O)_2_]*_n_*^2–^ and [H_2_V_10_O_28_Na(H_2_O)_8_]*_n_*^2–^, found in **3** and **4**, respectively, are 2D coordination polymers, where protonated decavanadate anions are interconnected by sodium atoms, forming chains. These anionic chains are further linked into a three-dimensional supramolecular framework through hydrogen bonding with neighboring cobalt(III) cations. They exhibit bidirectional hydrogen bonding, accepting hydrogen bonds at nonprotonated oxygen atoms and donating hydrogen bonds at protonated oxygen atoms. This interaction plays a key role in the self-assembly of decavanadates. These types of unique intermolecular interactions present in decavanadates are a consequence of their lower acidity.^40,41^ As part of our research, we performed ground state geometry optimizations on four decavanadates, [Co(NH_3_)_4_]_4_ [H_2_V_10_O_28_]·6H_2_O (**1**), [Co(NH_3_)_4_]_6_[V_10_O_28_]·16H_2_O (**2**), [Co(ox)(en)_2_]_4_[H_2_V_10_O_28_]·12H_2_O (**5**) and [Co(ox)(en)_2_]_6_[V_10_O_28_]·*n*H_2_O (*n* ≈ 13.5) (**6**) using density functional theory B3LYP/def2SVPP with the Grimme D3 correction and Becke–Johnson damping function. For all compounds, the electrostatic potential was mapped on the electron isodensity surface using the isodensity value of 0.001 au The electrostatic potentials for [Co(NH_3_)_4_]_4_[H_2_V_10_O_28_]·6H_2_O (**1**) and [Co(NH_3_)_4_]_6_[V_10_O_28_]·16H_2_O (**2**), are shown in Figure 2. These findings confirm that the most negative electrostatic potential resides within the metal core, while the outer ligands provide a less negative potential that stabilizes the more negative inner core. A similar trend is observed in decavanadates **5** and **6**, as depicted in Figure S13.

The influence of the protonation state on the hydrogen bonding and, consequently, on the overall structure is noteworthy. However, the size of the cations also plays a significant role in determining the key structural features. Specifically, larger cations lead to monomeric anions, whereas smaller cations promote the formation of polymeric anions.^42−44^ Furthermore, the cation size affects the stability of decavanadates and polyoxometalates in general. This has been confirmed by investigation of the reaction profile performed for the compounds [Co(NH_3_)_4_]_4_[H_2_V_10_O_28_]·6H_2_O (**1**), [Co(NH_3_)_4_]_6_[V_10_O_28_]·16H_2_O (**2**), [Co(ox)(en)_2_]_4_[H_2_V_10_O_28_]·12H_2_O (**5**), and [Co(ox)(en)_2_]_6_[V_10_O_28_]·*n*H_2_O (*n* ≈ 13.5) (**6**) obtained under reflux or solvothermal conditions. The analysis of relative binding energies revealed that products **5** and **6** are over 1100 kJ mol^–1^ more stable than products **1** and **2**. This finding implies that both procedures provide additional energy to the system, facilitating access to a wider landscape of possible outcomes. Therefore, identifying products, **5** and **6** as the more stable compounds is not only justified but also expected, as they contain the [Co(ox)(en)_2_]^+^ cation.

**Figure 3:**
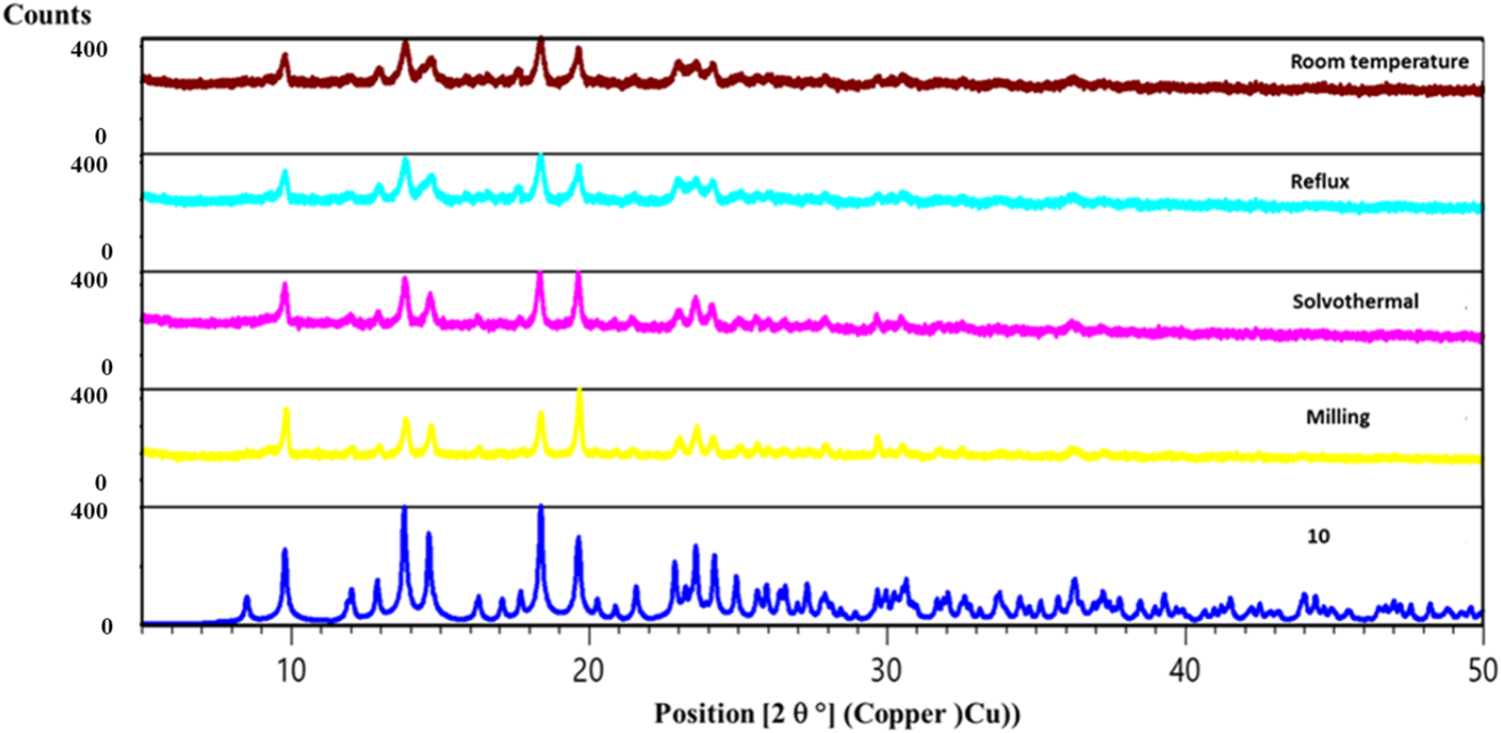
PXRD patterns of compound **10** (as yellow powder) obtained by different methods: reflux (brown), reflux (light blue), solvothermal (violet) mechanochemistry (yellow), and generated from SCXRD data of crystals of product **10** (dark blue).

However, in addition to protonated and nonprotonated decavanadates, in the reactions involving [Co(ox)(en)_2_]^+^ or [Co(en)_3_]^3+^ cations (Figure 1 and Table 1) we isolated two polymers, divanadate [V_2_O_6_Na(H_2_O)]*_n_*^–^ (in **9**) and a linear trivanadate [V_3_O_9_]*_n_*^3–^ (in **10**). Polymeric trivanadate was the only product in all reactions conducted in the presence of the [Co(en)_3_]^3+^ cation, without acid and regardless of the synthetic method used. The result was confirmed by PXRD experiments (Figures 3 and S12).

The isolation of [V_2_O_6_Na(H_2_O)]*_n_*^–^ and [V_3_O_9_]*_n_*^3–^ species is not unexpected, considering that at the given pH values, different oxovanadate species are in equilibrium. Investigations of the stability of decavanadates in aqueous solution as a function of pH showed that under acidic conditions (pH between 5 and 6), the disassembly of [H_*x*_V_10_O_28_]^(6–x)–^ is significantly slower, compared to that under basic conditions. After several hours, [V_4_O_12_]^4–^ units were observed in the mixture with [H_*x*_V_10_O_28_]^(6–x)–^.^45^

The reactions with the [Co(en)_3_]^3+^ cation in the presence of acids resulted in an insoluble flocculent powder of an undefined composition. It can be assumed that this powder is a mixture of products that cannot be separated based on solubility. Similarly, reactions performed with the [Co(NH_3_)_6_]^3+^ cation under the same conditions led to the isolation of an inhomogeneous powder, which was insoluble in any solvent.

**Scheme 1:**
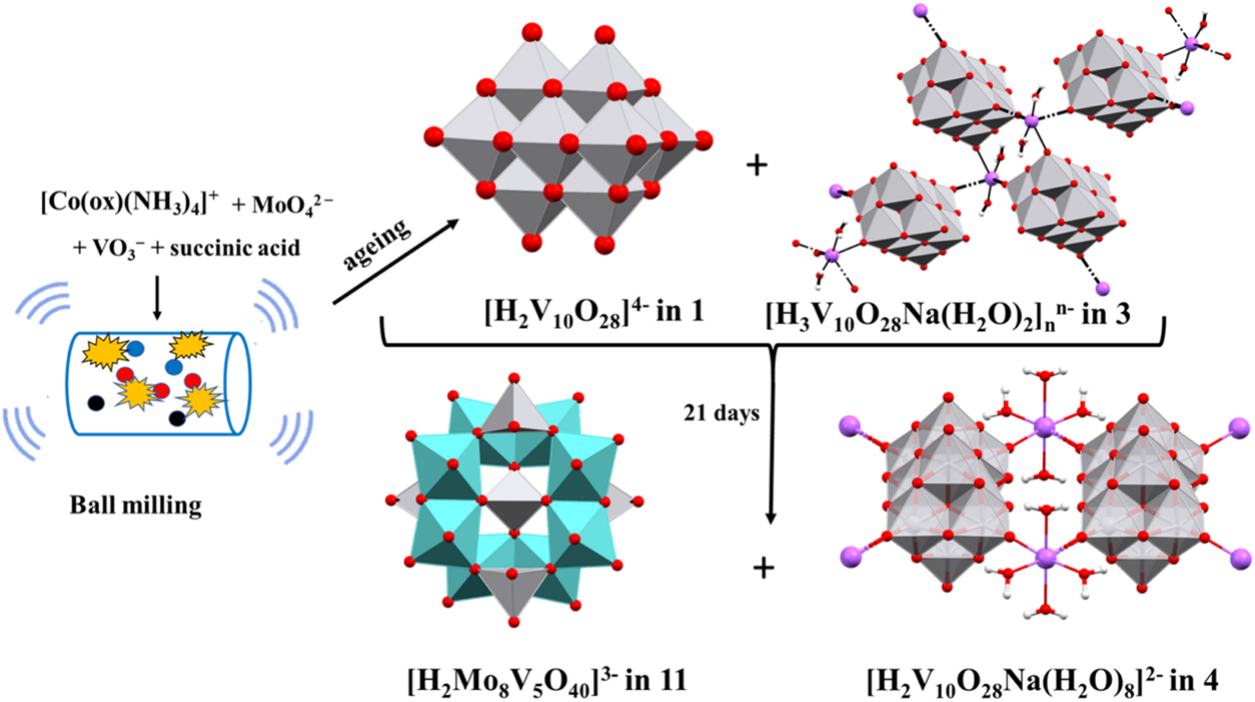
Transformation of [Co(ox)(NH_3_)_4_]_4_[H_2_V_10_O_28_]·6H_2_O (**1**) and [Na(H_2_O)_2_]*_n_*[H_3_V_10_O_28_Na(H_2_O)_2_]*_n_*·3*n*H_2_O^37^ (**3**) to [Co(ox)(NH_3_)_4_]_2_[H_2_V_10_O_28_Na(H_2_O)_8_]·8H_2_O (**4**) and [Co(ox)(NH_3_)_4_]_3_[H_2_Mo_8_V_5_O_40_Na_2_(H_2_O)_8_]·5.5H_2_O (**11**) While Exposing the Reaction Mixture to Water Vapor

Mechanochemically conducted reactions accompanied by exposure of the powder to water vapor yielded interesting results. Reaction of the [Co(ox)(NH_3_)_4_]^+^ cation with molybdate and vanadate anions, regardless of the added acid and exposure of the powder to water vapor initially produced a mixture of rose-colored powder, di- and triprotonated decavanadates **1** and **3**. After 21 days, that mixture transformed into a two-dimensional polymer, **4**, and molybdovanadate **11** (Scheme 1). In reactions without the molybdate anion and without acid, the only product obtained was **1**. However, in the presence of the molybdate anion, molybdovanadate **11** was successfully isolated.

**Scheme 2:**
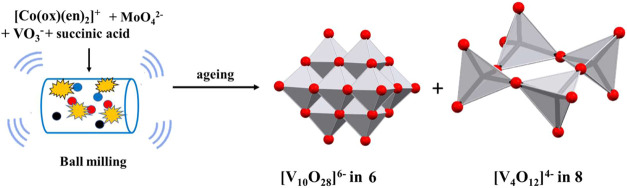
Formation of [Co(ox)(en)_2_]Cl·4H_2_O, [Co(ox)(en)_2_]_6_[V_10_O_28_]·*n*H_2_O (*n* ≈ 13.5) (**6**), and [Co(ox)(en)_2_]_4_[V_4_O_12_]·14H_2_O (**8**) While Exposing the Reaction Mixture to Water Vapor

Mechanochemical reactions also yielded protonated and unprotonated decavanadates similar to solution-based reactions (Figure 1 and Table 1). The reaction of VO_3_^–^, [Co(ox)(en)_2_]^+^, succinic acid, and sodium molybdate produced a powdered product and crystals of tetrahydrate Co(III) complex, [Co(ox)(en)_2_]Cl·4H_2_O^46^ which disappear within a few hours, leading to the formation of products **6** and **8** formed (Scheme 2). Reactions carried out with or without the molybdate anion and without acid resulted in polymer **9**.

In the reactions involving the [Co(en)_3_]^3+^ cation, molybdate, and vanadate anion without acid, dark yellow sticks of **12** and light-yellow sticks of **10** were obtained. In contrast, a reaction conducted without the molybdate anion and in the presence of acid resulted in gray-yellow insoluble powder.

### Spectroscopic and Thermal Investigations

**Figure 4:**
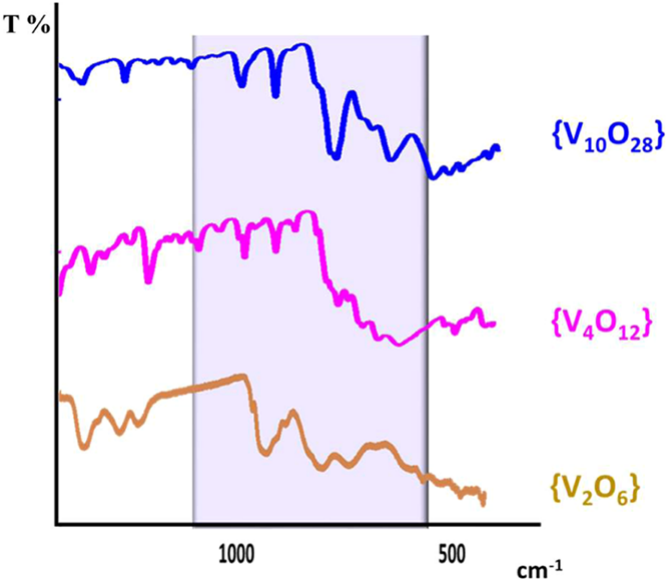
Part of the IR spectra, between 1000 and 500 cm^–1^, of polyoxovanadates built up from [V_2_O_6_(Na(H_2_O))]^2–^, [V_4_O_12_]^4–^, and [V_10_O_28_]^6–^ anions.

As expected, IR spectra of all oxovanadates reveal the presence of vanadium–oxygen IR stretching frequencies, which also depend on the type of cation associated with the anion (Figure 4).

The peaks in the range of 998–1055 cm^–1^ are ascribed to terminal V–O bonds, strong bands at 855 and 752 cm^–1^ and at 657 and 514 cm^–1^ are assigned to the antisymmetric stretching vibrations of V–O–V bond. The broad and medium intensity bands present at 3498–3286 cm^–1^ confirmed the existence of the O–H···O hydrogen bonds. In the IR spectra of all of the compounds, the strong bands at 1744–1699 and 1709–1280 cm^–1^ were assigned to the asymmetric and symmetric stretching vibrations of C–O and C–C from the oxalate or ethylenediamine ligand present in Co(III) complex cation (in **1**, **2**, **4**–**12** and in yellow powder) (Table S2 and Figures S18 and S19).

The thermogravimetric studies were conducted on products **1**, **2**, **3**, **5**, **6**, **7**, **9**, **10**, and **11**, under an oxygen atmosphere and in the temperature interval of 25–600 °C (Figures S2–S11). The dehydration and decomposition of the anhydrous part of the examined compounds were observed, and agreed with other investigations, *e*.*g*. structural and chemical analysis and literature data.^47^ The decompositions of compounds **1**–**3**, **5**–**7**, **9**–**11**, and yellow powder (identified as compound **10**) proceeded in three steps (two in case of **2** and **6**), starting with the endothermic process associated with the loss of water molecules of crystallization. The thermograms showed a weight loss of 6.55% for **1** (calc. 5.6%), 12.89% for **2** (calc. 11.36%), 5.66% for **3** (calc. 4.16%), 8.38 for **5** (calc. 9.62%), 10.28 for **6** (calc. 10.11%), 9.48% for **7** (calc. 8.18%), 7.32% for **9** (calc. 7.06%), 3.18% for **10** (calc. 2.89%), in the temperature range of 32–189 °C. The thermogram **11** showed a weight of 6.89% (calc. 7.42%), in the higher temperature range, 32–282 °C. The higher temperature could be the result of stronger hydrogen bonds. The further weight loss can be attributed to the degradation of the unsolvated species in two distinct stages or one (for **2** and **6**). The first ones occurred in the temperature range of 148–405 °C (for **1**; 148–394 °C; for **2**, 189–465 °C; for **3**, 163–364 °C; for **5**, 148–394 °C; for **6**, 185–483 °C; for **7**, 163–364 °C; for **9**, 203–369 °C; for **10**, 200–332 °C; for **11**, 282–342 °C) and the second stage in the range of 328–485 °C (for **1**; 395–480 °C; for **3**, 354–485 °C; for **5**, 395–480 °C; for **7**, 354–485 °C; for **9**, 371–436 °C; for **10**, 340–408 °C; for **11**, 342–389 °C). These stages represent the decomposition of Co cation and oxovanadate cores. The residual solids consisted of mixtures of Co_*x*_V_*y*_O_*z*_ (of **1**, **2**, **5**, **6**, **7**) or NaCo_*x*_V_*y*_O_*z*_ (of **3**, **6**) or NaCo_*x*_Mo_*z*_V_*y*_ (of **11**).

## Crystal Studies

**Figure 5:**
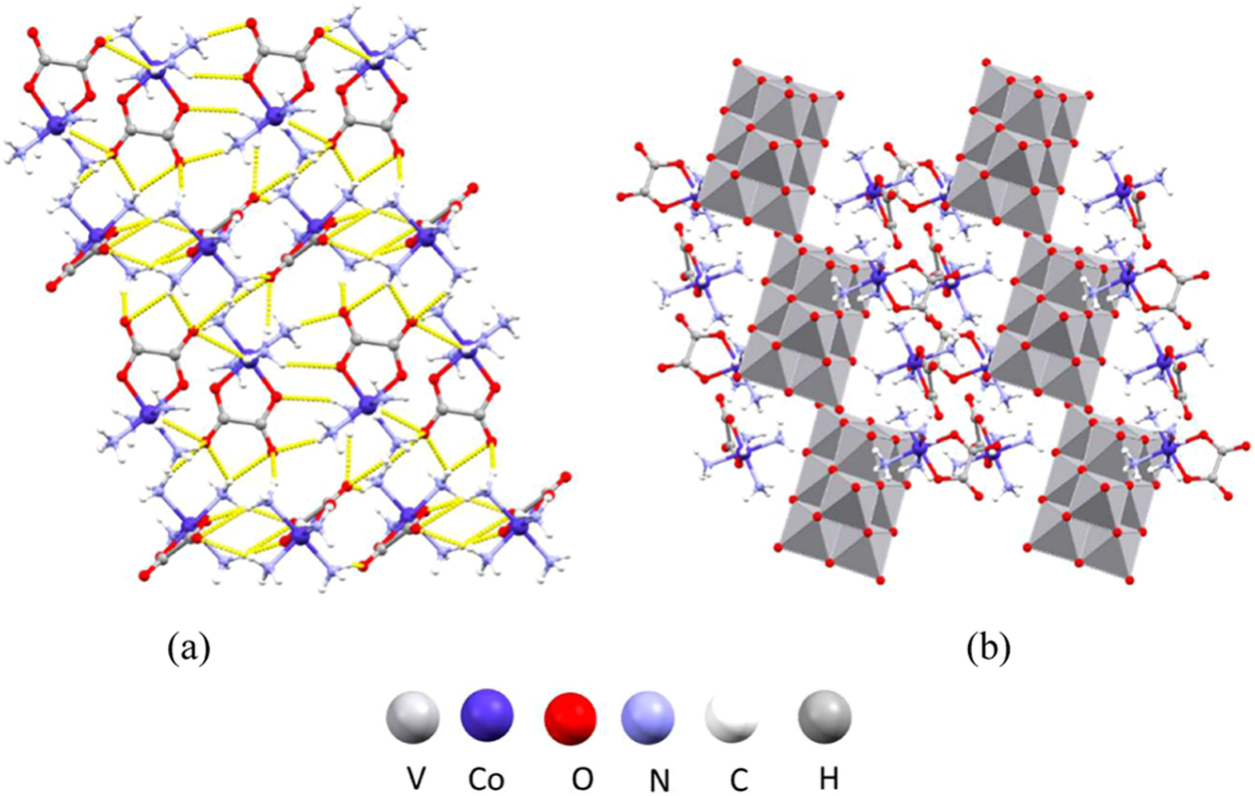
Structure of **1**: (a) hydrogen-bonded layer of cations perpendicular to the crystallographic *c*-axis; (b) crystal packing in the unit cell viewed along the crystallographic *b*-axis.

Crystallographic data for compounds **1**–**9** is given in Table S4. The unit cell of [Co(ox)(NH_3_)_4_]_4_[H_2_V_10_O_28_]·6H_2_O (**1**) (Figure S1a) comprises cobalt(III) cations [Co(ox)(NH_3_)_4_]^+^, and a doubly protonated decavanadate anion [H_2_V_10_O_28_]^4–^, with two formula units per unit cell. The geometry of the anion is consistent with those found in the previously reported structures.^48^ The cations form a two-dimensional hydrogen-bonded layer perpendicular to the crystallographic *c*-axis (Figure 5a). The NH_3_ groups of the cation form 21 hydrogen bonds with oxygen atoms of oxalate ligands of neighboring cations (Table S5). The layers are further interconnected by hydrogen bonding with the oxo ligands of the polyoxovanadate. Each anion forms 15 hydrogen bonds with 10 neighboring cations (Table S5). Anions and cations form an extensive three-dimensional supramolecular framework that contains crystallization water molecules (Figure 5b).

**Figure 6:**
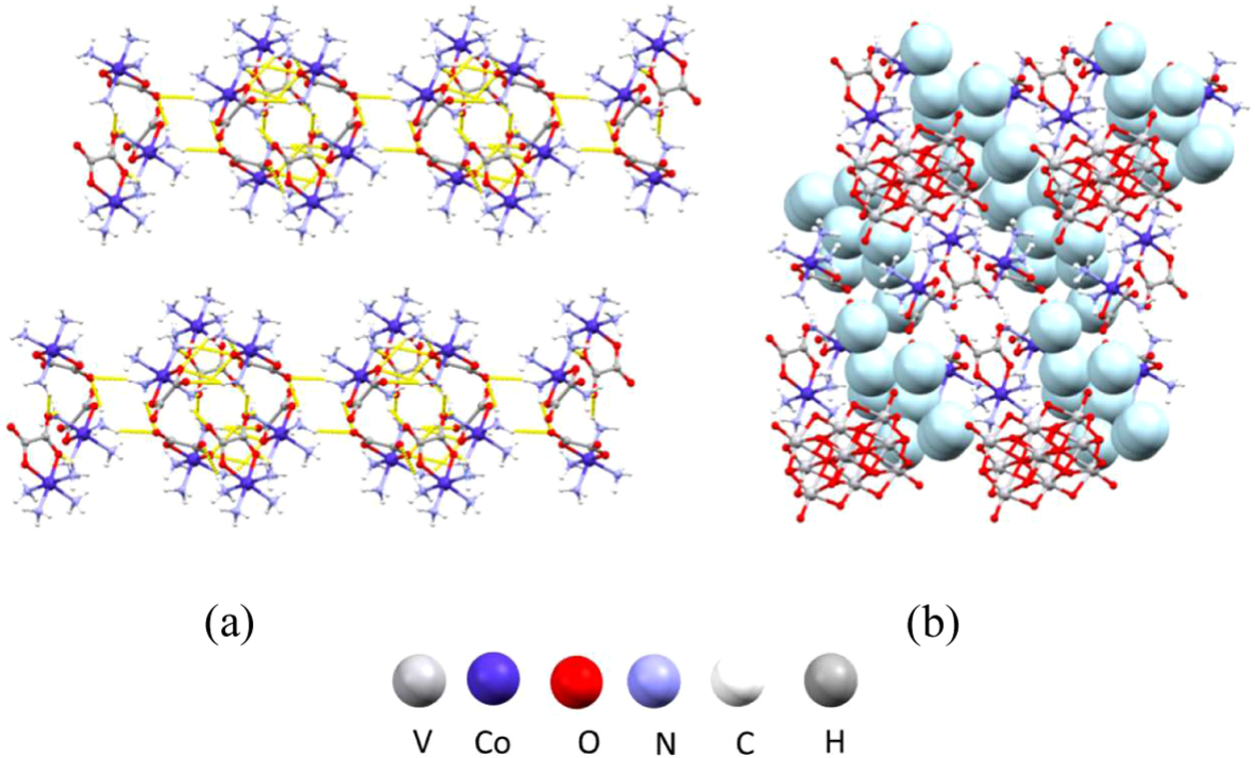
Structure of **2**: (a) hydrogen-bonded chains of cations along the crystallographic *b*-axis; (b) crystal packing viewed along the crystallographic *a*-axis with channels containing water molecules (light blue).

Just as compound **1**, [Co(ox)(NH_3_)_4_]_6_[V_10_O_28_]·16H_2_O (**2**) (Figure S1b) crystallizes in *P*-1 space group, but with a considerably larger unit cell, since it contains two additional [Co(ox)(NH_3_)_4_]^+^ cations and water molecules. The decavanadate [V_10_O_28_]^6–^ cation in **2** is not protonated and its geometry aligns with those found in the previously reported structures.^49^ The cations are connected into chains that run along the crystallographic *a*-axis *via* 18 hydrogen bonds (Table S7) formed between NH_3_ ligands and oxalate ligands of the neighboring cations (Figure 6a). The NH_3_ groups also act as hydrogen bond donors toward the oxo ligands of the polyanion with them they form 15 hydrogen bonds (Table S6). Anions and cations are interconnected into a supramolecular framework that forms channels containing water molecules (Figure 6b).

**Figure 7:**
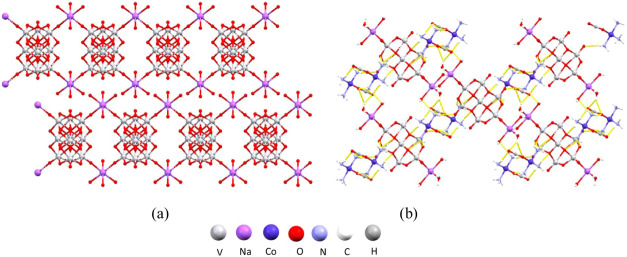
Structure of **4**: (a) polyanionic chains along the crystallographic *b*-axis (hydrogen atoms were omitted for clarity); (b) crystal packing in the unit cell viewed along the crystallographic *b*-axis.

Compound [Co(ox)(NH_3_)_4_]_2_[H_2_V_10_O_28_Na(H_2_O)_8_]·8H_2_O (**4**) (Figure S1c) is a two-dimensional polymer comprising doubly protonated decavanadate {H_2_V_10_O_28_}^4–^ units interconnected by sodium ions forming chains of [H_2_V_10_O_28_Na(H_2_O)_8_]^3–^ along the crystallographic *b*-axis. Decavanadate anions are coordinated by four sodium atoms, with bridging oxo ligands bonded to sodium atoms being in the *cis* position. Each sodium atom is further coordinated with four additional water molecules (Figure 7a). The anionic chains are interconnected into a three-dimensional supramolecular framework by hydrogen bonding to the neighboring cobalt(III) cations [Co(ox)(NH_3_)_4_]^+^ with which they form four N–H···O hydrogen bonds (N2–H2B···O6 of 3.233(6) Å, N2–H2C···O6 of 3.233(6) Å, N3–H3C···O2 of 2.994(7) Å, and N4–H4B···O7 of 3.041(10) Å) (Figure 7b). The cations and anions are arranged in a way that leaves spaces within the structure that accommodate crystallized water molecules.

**Figure 8:**
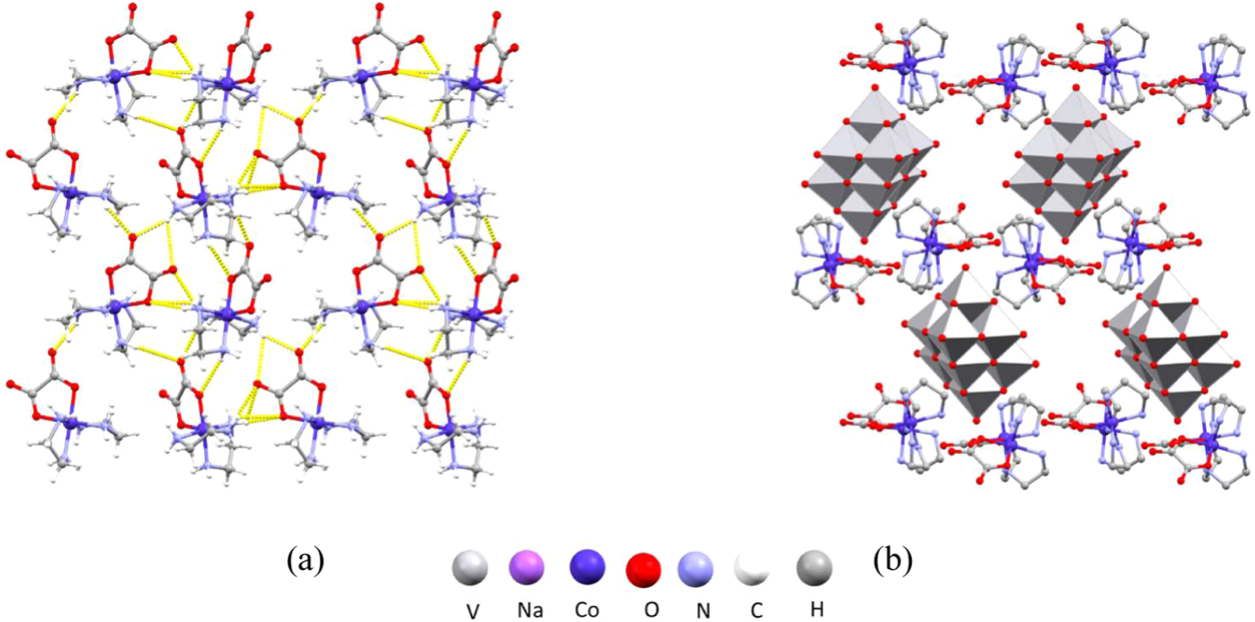
Structure of **5**: (a) hydrogen-bonded layer of cations perpendicular to the crystallographic *c*-axis; (b) crystal packing in the unit cell viewed along the crystallographic *a*-axis (hydrogen atoms were omitted for clarity).

Compound [Co(ox)(en)_2_]_4_[H_2_V_10_O_28_]·12H_2_O (**5**) (Figure S1d) consists of four [Co(ox)(en)_2_]^+^ cations and a doubly protonated decavanadate anion [H_2_V_10_O_28_]^4–^ in an asymmetric unit. The ethylenediamine ligands on the complex cobalt cations serve as the hydrogen bond donors, with the oxygen atoms of the oxalate ligands of the neighboring cations being the hydrogen bond acceptors. The cations are thus connected to a two-dimensional supramolecular layer that is oriented perpendicular to the crystallographic *c*-axis (Figure 8a). Cations mutually form eight N–H···O hydrogen bonds (Table S7). The cation layers are further interconnected by hydrogen bonding to the decavanadate polyanions, forming an extensive supramolecular framework with areas containing water molecules (Figure 8b). Anions and cations form altogether 11 hydrogen bonds (Table S7).

**Figure 9:**
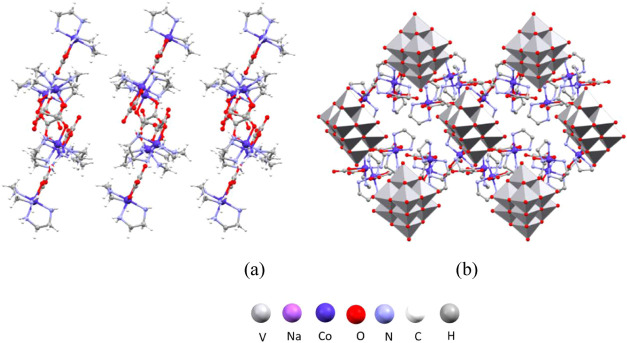
Structure of **6**: (a) layers of cations perpendicular to the crystallographic *b*-axis; (b) crystal packing in the unit cell viewed along the crystallographic *c*-axis (hydrogen atoms were omitted for clarity).

In compound [Co(ox)(en)_2_]_6_[V_10_O_28_]·*n*H_2_O (*n* ≈ 13.5) (**6**) (Figure S1e) the decavanadate anion [V_10_O_28_]^6–^ is not protonated and its relatively large negative charge is compensated by six [Co(ox)(en)_2_]^+^ cations. The ethylenediamine ligands of the complex Co(III) cations participate in the formation of 21 N–H···O hydrogen bonds with the oxalate ligands of the neighboring cations (Table S8), as well with water molecules, forming layers perpendicular to the crystallographic *b*-axis (Figure 9a). The cation layers contain grooves in which decavanadate anions are positioned (Figure 9b). The oxo ligands of the vanadate anion act as acceptors of 24 N–H···O hydrogen bonds formed with the surrounding cations (Table S8).

**Figure 10:**
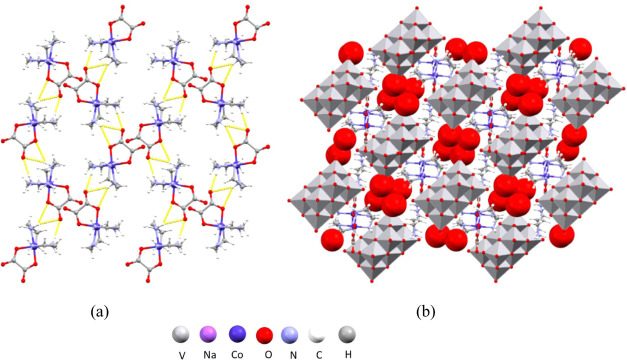
Structure of **7**: (a) chains of cations parallel to the crystallographic *b*-axis; (b) crystal packing in the unit cell viewed along the crystallographic *b*-axis.

In compound (H_2_en)_2_[Co(ox)(en)_2_]_2_[V_10_O_28_]·*n*H_2_O (*n* ≈ 2) (**7**) (Figure S1f) the negative charge of the [V_10_O_28_]^6–^ anion is compensated not only by complex cobalt(III) cations [Co(ox)(en)_2_]^+^, but also by doubly protonated diethylammonium cations. This compound crystallizes in the *P*2_1_/*n* space group as an octahydrate complex salt. The diethylammonium ligands of the complex cation form three N–H···O hydrogen bonds with the oxalate ligands of the neighboring cations (Table S9) connecting them into chains that run parallel to the crystallographic *b*-axis (Figure 10a). Each decavanadate anion participates in the formation of eight hydrogen bonds with the cobalt(III) cations and two hydrogen bonds with the diethylammonium cations (Table S9) interconnecting them into a three-dimensional supramolecular framework with channels filled with water molecules (Figure 10b).

**Figure 11:**
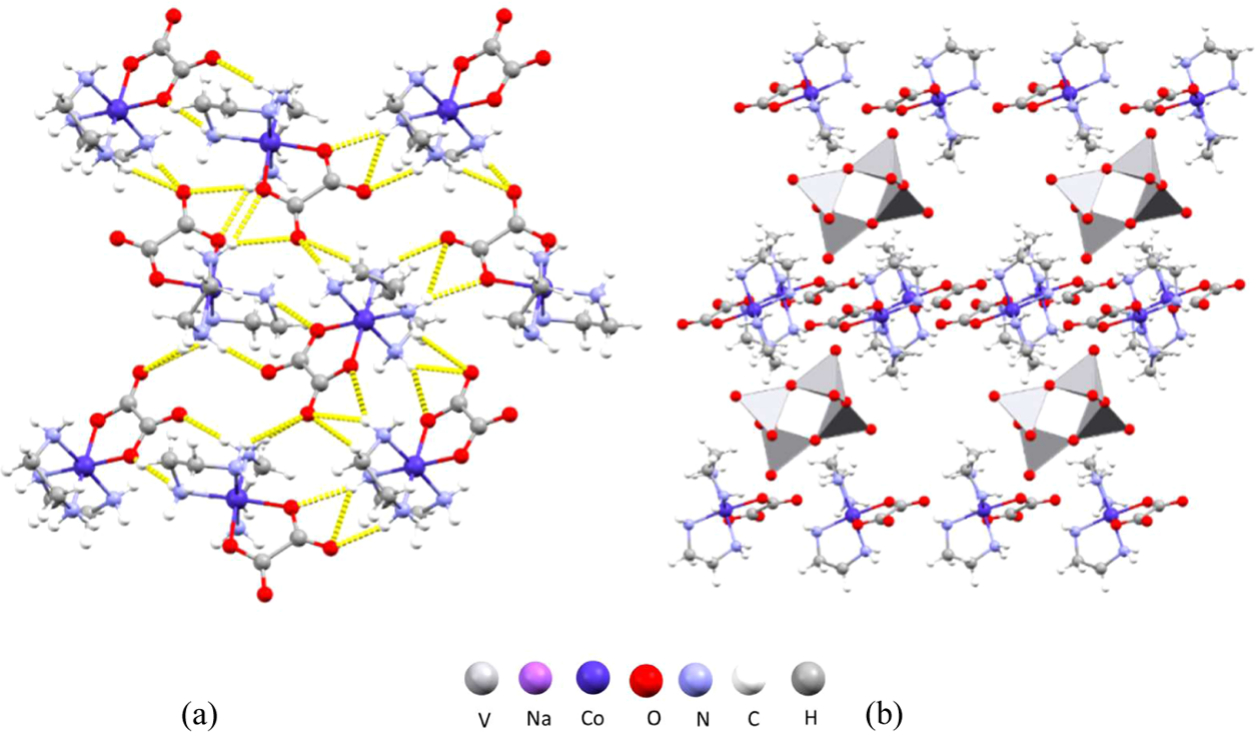
Structure of **8**: (a) hydrogen-bonded layers of anions perpendicular to the crystallographic *a*-axis; (b) crystal packing in the unit cell viewed along the crystallographic *b*-axis with layers of cations and tetravanadate anions between them.

In compound [Co(ox)(en)_2_]_4_[V_4_O_12_]·14H_2_O (**8**) (Figure S1g) a tetranuclear polyvanadate anion [V_4_O_12_]^4–^ and four complex cobalt(III) cations [Co(ox)(en)_2_]^+^ are present. The vanadate anion is built of four vertex-sharing [VO_4_] tetrahedra and its structure is in accordance with the ones previously reported.^50^ The oxygen atoms of the oxalate ligand serve as acceptors for 15 hydrogen bonds, which are formed with the ethylenediamine ligands of the neighboring cations (Table S10). Cations are interconnected *via* hydrogen bonding into two-dimensional supramolecular layers perpendicular to the crystallographic *a*-axis (Figure 11a). Tetravanadate anions are situated between the cation layers, bridging them by forming two hydrogen bonds with the ethylenediamine ligands (Table S10) making the overall structure a three-dimensional supramolecular framework (Figure 11b).

**Figure 12:**
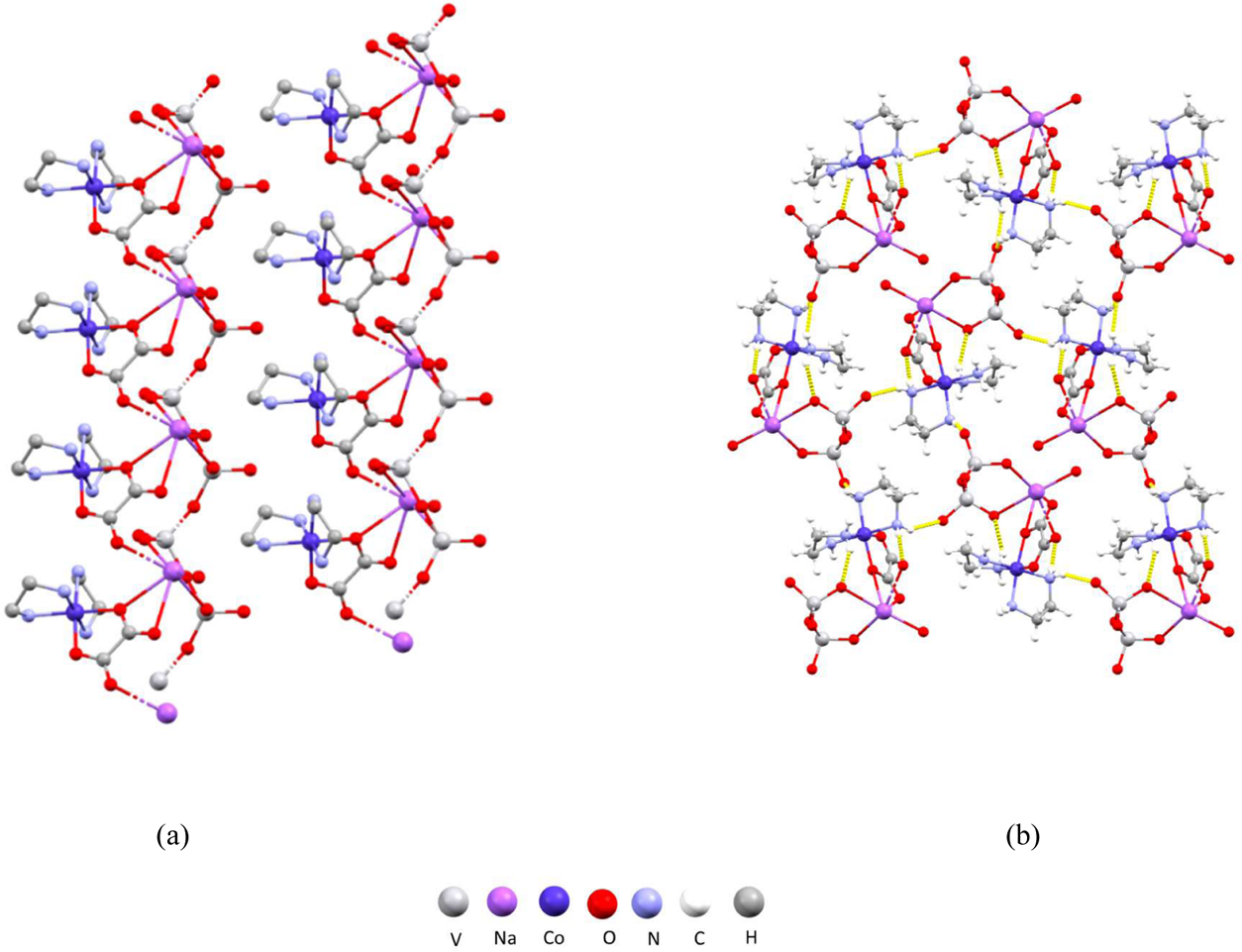
Structure of **9**: (a) polymeric chains comprising sodium and complex cobalt(III) cations and polyvanadate anions running along the crystallographic *a*-axis; (b) crystal packing in the unit cell viewed along the crystallographic *a*-axis with polymeric chains interconnected *via* hydrogen bonds.

Compound [Co(ox)(en)_2_]_n_[V_2_O_6_Na(H_2_O)]_n_·*n*H_2_O (**9**) (Figure S1h) is a one-dimensional polymer comprising polyvanadate {V_2_O_6_} unit, [Co(ox)(en)_2_]^+^ cation and sodium cation. The vanadate chains consist of vertex-sharing {VO_4_} tetrahedra with one oxo ligand on every vanadium atom also coordinated to the sodium cation. Each sodium cation is coordinated by seven oxygen atoms: two oxo ligands from the vanadate chain, three from the oxalate ligands of two neighboring complex cobalt(III) cations, and two water molecules, making the geometry around the sodium atom a distorted capped octahedron. Anion and cations form chains that span along the crystallographic *a*-axis (Figure 12a). The chains are further interconnected by hydrogen bonds formed by the ethylenediammonium ligands coordinated to cobalt cations with oxo ligands of the vanadate chains (Figure 12b). Overall, cations form six N–H···O hydrogen bonds with the vanadate, forming a three-dimensional supramolecular framework that leaves voids containing water molecules (Table S11).

## Catalytic Experiments

Catalytic experiments were conducted by using benzyl alcohol as the substrate and *tert-*butyl hydroperoxide (TBHP) as the oxidizing agent. It is known that the oxidation of benzyl alcohol can yield different products depending on the reaction conditions. For example, partial oxidation can produce benzaldehyde, while complete oxidation results in benzoic acid. Furthermore, benzyl alcohol can undergo oxidation followed by ester formation, or in some cases, benzyl alcohol undergoes dehydration instead of oxidation, forming dibenzyl ether as a byproduct. The desired product in this research was benzaldehyde, although benzoic acid was also detected in the chromatogram. However, benzoic acid was not quantified due to its solubility in the water phase while the analyzed phase by GC was the organic one.

The catalysts investigated included reference compounds [Co(ox)(NH_3_)_4_]NO_3_·H_2_O (**I**), [Co(ox)(en)_3_]NO_3_ H_2_O (**II**), [Co(ox)(en)_2_]Cl·1.5H_2_O (**III**), [Co(ox)(NH_3_)_4_]_4_[Na_2_Mo_8_O_29_(H_2_O)_4_]·6H_2_O (**IV**), [Co(ox)(NH_3_)_4_]_4_[Mo_8_O_26_]·4H_2_O·C_4_H_6_O_4_ (**V**), and [Co(ox)(NH_3_)_4_]_4_[Mo_8_O_26_]·12H_2_O (**VI**). These catalysts represent diverse structural motifs, including cobalt complexes with either nitrate, chloride, or polyoxometalate anions, and were evaluated for their activity and selectivity in the oxidation of benzyl alcohol. All of the results are compiled in Table 2.

**Table 2:** Catalytic Results of Benzyl Alcohol Oxidation^a^

	conversion^b^, %		selectivity^c^, %	
catalyst	300 min	20 min	300 min	20 min
[Co(ox)(NH_3_)_4_]NO_3_·H_2_O (**I**)	16	4	89	54
[Co(ox)(en)_3_]NO_3_·H_2_O (**II**)	41	4	64	99
[Co(ox)(en)_2_]Cl·1.5H_2_O (**III**)	17	6	71	32
[Co(ox)(NH_3_)_4_]_4_[Na_2_Mo_8_O_29_(H_2_O)_4_]·6H_2_O (**IV**)	27	4	78	56
[Co(ox)(NH_3_)_4_]_4_[Mo_8_O_26_]·4H_2_O·C_4_H_6_O_4_ (**V**)	23	5	78	76
[Co(ox)(NH_3_)_4_]_4_[Mo_8_O_26_]·12H_2_O (**VI**)	21	1	84	79
[Co(en)_3_][V_3_O_9_]*_n_*·*n*H_2_O (**10**)	45	6	62	79
[Co(ox)(NH_3_)_4_]_4_[H_2_V_10_O_28_]·6H_2_O (**1**)	82	12	21	75
[Co(ox)(NH_3_)_4_]_6_[V_10_O_28_]·16H_2_O (**2**)	69	7	40	71
[Co(ox)(NH_3_)_4_]_4_[H_2_Mo_8_V_5_O_40_Na_2_(H_2_O)_8_]·5.5H_2_O (**11**)^d^	92	21	10	49
[Co(ox)(en)_2_]_4_[ H_2_V_10_O_28_]·12H_2_O (**5**)	72	18	29	67

a Reaction conditions: time, 5 h; temperature, 80 °C, *n*(cyclooctene)/*n*(oxidant) = 20 mmol/40 mmol.

b Alcohol consumed at 20 min and at the end of reaction.

c *n*(alcohol) transformed/*n*(catalyst)/time (h) at 20 min and at the end of the reaction.

d The reaction was followed for 180 min and not 300.

**Figure 13:**
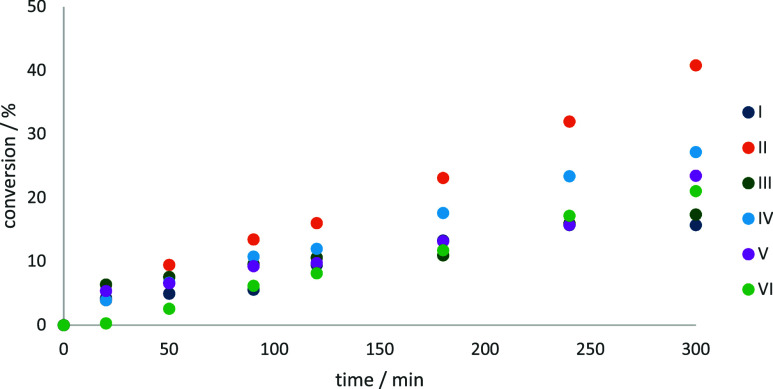
Kinetic profiles for benzyl alcohol oxidation with catalysts **I**–**VI**.

The polyoxometalate-based catalysts **IV**–**VI**^29,51^ featuring octamolybdate cores and cobalt cationic components exhibited relatively low benzyl alcohol conversion rates (21–27%) after 300 min, with the kinetic profiles as presented in Figure 13. However, they demonstrated high selectivity toward benzaldehyde, ranging from 72 to 84%, Figure 13. This combination of moderate activity and high selectivity highlights the role of the octamolybdate framework in favoring partial oxidation to the aldehyde without overoxidation to carboxylic acids or other byproducts.

Conversely, the cobalt complexes with nitrate or chloride counterions **I** and **III** showed varying catalytic activities. Among these, **II** was the most active, achieving a benzyl alcohol conversion of 41%, while **I** and **III** displayed lower conversion rates, approximately 15%, Figure 13. Interestingly, the selectivity trends for benzaldehyde followed the order **II** (63%) < **I** (71%) < **III** (89%), suggesting that the chloride-containing complex **III** is particularly effective at suppressing overoxidation pathways, Figure 13.

**Figure 14:**
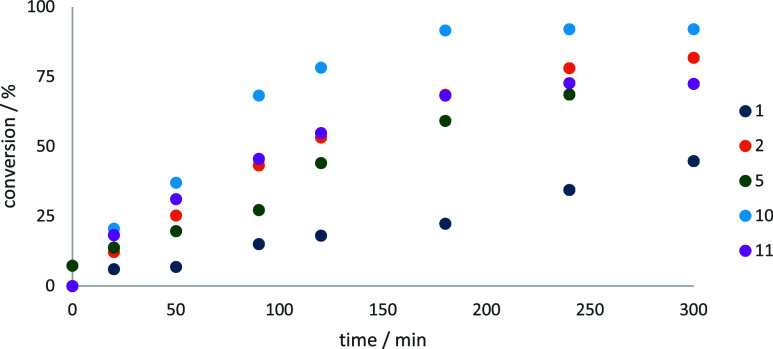
Kinetic profiles for benzyl alcohol oxidation with catalysts **1**, **2**, **5**, **10**, and **11**.

Catalytic activity was evaluated for polyoxometalates **1**, **2**, **5**, **10**, and **11**. Among these, **11** exhibited distinct catalytic behavior, Figure 14. After 20 min of reaction, it achieved a benzyl alcohol conversion of 21% with 50% selectivity toward benzaldehyde. However, prolonged reaction times led to a decline in the aldehyde yield, concurrent with the appearance of benzoic acid in the chromatogram. Benzoic acid, being water-soluble, partitioned partially into the aqueous phase, which was not analyzed and, thus, not quantified. After 180 min, the reaction plateaued at a conversion of 91%, but aldehyde selectivity decreased to 10%, Figure 14. It can be concluded that the inclusion of molybdenum (as in {H_2_Mo_8_V_5_O_40_}) affects the catalytic behavior, enhancing initial selectivity but promoting secondary oxidation over time.

**Figure 15:**
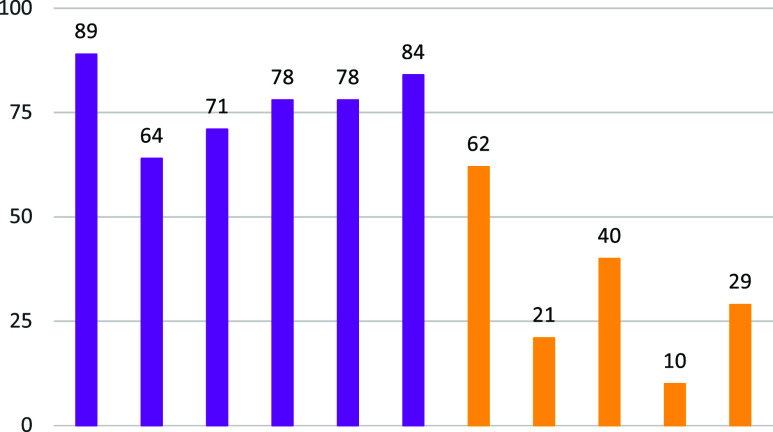
Comparison of the aldehyde selectivity for (from left to right): **I**, **II**, **III**, **IV**, **V**, and **VI** (purple bars) and **1**, **2**, **5**, **10**, and **11** (yellow bars).

Similar behavior was observed for the other complexes. Benzyl alcohol conversion values of 45% (**10**), 69% (**2**), 73% (**5**), and 82% (**1**) were recorded after 180 min. Aldehyde selectivity (Figure 15) showed an inverse trend, with values of 62% (**10**), 41% (**2**), 28% (**5**), and 21% (**1**). The type of cobalt coordination plays a crucial role, with ethylenediamine ligands promoting selectivity, while NH_3_ and C_2_O_4_-based ligands enhance activity but lead to aldehyde conversion.

The nitrate- and chloride-based cobalt complexes exhibit higher activity, though selectivity varies significantly, while the polyoxometalate-based catalysts excel in selectivity, but their activity is limited. These findings suggest that the catalytic performance depends on the nature of the cobalt complex, including the ligand environment and counterion. The observed significant activity when cobalt is coordinated with ammonia or oxalate as ligands compared to cobalt coordinated with ethylenediamine may be related to the greater stability of the ethylenediamine complex cation, [Co(en)_3_]^3+^. Also, the above results also suggest that the structural features of polyoxometalates play an important role in their activity. It is well known that the coordination environment around the V(V) center significantly impacts the catalytic activity during alcohol oxidation.^52^ Catalysts based on {V_4_O_12_} and {V_6_O_17_} demonstrate better activity for alcohol oxidation compared to that of those based on {V_10_O_28_}. These results indicate that vanadium centers situated in an unsaturated coordination environment – specifically, VO_4_ in tetrameric or hexametric forms – exhibit better activity than those in a saturated coordination mode, such as VO_6_ found in the decavanadate structure, for the oxidation of substrate.

## Conclusions

The synthesis of poloxovanadates and molybdovanadates demonstrated the influence of the cation type, reaction conditions, and acidity on product formation. Protonated decavanadates were identified as the most stable species in acidic environments, forming intricate supramolecular frameworks with cobalt(III) cations. Cation size played a crucial role in determining whether monomeric or polymeric anions would form, directly impacting the overall structure and stability of the compounds. The isolation of divanadates and trivanadates, along with the consistent formation of specific products from certain ion combinations, highlights the complexity of these reactions. Stability analyses indicated that the disassembly of decavanadates is significantly slower under acidic conditions. Additionally, the protonation state of cations significantly impacts hydrogen bonding, which, in turn, affects molecular assembly, particularly in the formation of monomeric or polymeric anions. Understanding how these factors intertwine to influence the arrangement of vanadate units remains a significant challenge for scientists.

Mechanochemical methods provided diverse products, suggesting promising avenues for further exploration in the synthesis and applications of polyoxometalate compounds. We found that this method could be a valuable technique, especially when it comes to isolating reaction intermediates. In our investigation of the catalytic activity of selected decavanadates in alcohol oxidation reactions, we demonstrated how the coordination environment around the V(V) center and Co(III)-cation significantly impacts the catalytic activity.

## Experimental Section

### Materials and Instruments

Starting cobalt(III) complex salts, [Co(ox)(NH_3_)_4_](NO_3_)·H_2_O, [Co(NH_3_)_6_]Cl_3_, [Co(en)_3_]Cl_3_, and [Co(ox)(en)_2_]Cl·1.5H_2_O used as reaction precursors, were prepared according to the literature data.^53,54^ NH_4_VO_3_, acetic, and succinic acid were commercially available reagent-grade chemicals that were employed as received without further purification. Elemental analyses (C, H, N, Co, Mo, V, and Na) were provided by the Analytical Services Laboratory of the Ruđer Bokovi Institute, Zagreb and by the Analytical Services Laboratory of the Faculty of Science and Faculty of Forestry, University of Zagreb (Table S3). Thermal studies (TGA-SDTA) were performed on a Mettler Toledo TGA/DSC 3+ STARe Systems instrument using aluminum oxide crucibles under an O_2_ atmosphere and in the temperature range from 25 to 600 °C. The heating rate was 10 °C min^**–**1^ (Figures S2–S11). Infrared spectra were recorded on a PerkinElmer Spectrum RXI FTIR-ATR spectrometer in the 4000–400 cm^**–**1^ range (Figures S18 and S19). Ground state geometry optimizations for compounds in investigated reactions were performed using hybrid functional B3LYP^55,56^ with the D3 version of Grimme’s dispersion^57^ and Becke Johnson damping function combined with the def2SVPP basis set. Harmonic vibrational frequencies were calculated for all optimized structures to ensure that the obtained geometries correspond to the minimum on the potential energy surface.^58^ Standard Gibbs energies of formation were calculated at *T* = 298.15 K and *p* = 101325 Pa and used for estimation of the reaction energies. Electrostatic potentials were mapped on the electron density isosurface (0.001 au) of optimized structures. All calculations were performed using the Gaussian 16 software.^59^ The catalytic oxidation of benzyl alcohol was monitored using gas chromatography (GC) performed on an Agilent 8860 chromatograph (Agilent Technologies, Santa Clara, CA). The instrument was equipped with a flame ionization detector (FID) and an HP-5 column (30 m × 0.320 mm × 0.25 μm). Reaction parameters were quantified using calibration curves generated from the authentic standards of the reactants and products. The conversion of benzyl alcohols to respective aldehydes was determined relative to the internal standard, biphenyl. Calibration curve correlation coefficients (*R*^2^) consistently reached 0.9999, demonstrating the accuracy of the quantification. Single-crystal X-ray diffraction data of **1**, **2**, and **4–9** were collected on a XtaLAB Synergy-SCCD diffractometer with Cu Kα (λ = 1.54184 Å) radiation at room temperature or at 170 K. Data reduction was performed using the CrysAlis software package.^60^ Solution, refinement, and analysis of the structures were done using the programs integrated in the WinGX^61^ and OLEX2^62^ systems. All structures were solved and refined with the SHELX program suite.^63^ Structural refinement was performed on *F*^2^ using all of the data. All hydrogen atoms were placed at calculated positions and treated as riding on their parent atoms. Geometrical calculations were done using PLATON.^64^ Drawings of the structures were prepared using PLATON and MERCURY programs.^65^ Powder X-ray diffraction (PXRD) data were collected on a Malvern Panalytical Aeris powder diffractometer in the Bragg–Brentano geometry with a PIXcel^1D^ detector, using Cu Kα radiation (λ = 1.5406 Å). Samples were contained on a Si sample holder. Powder patterns were collected at room temperature in the range from 5 to 50°(2θ) with a step size of 0.043 and 7.14 s per step (Figures S12–S17). The data were collected and visualized by using the Malvern Panalytical HighScore Software Suite.^66^ Crystallographic information files are available from the Cambridge Crystallographic Data Centre (CCDC) upon request (http://www. ccdc.cam.ac.uk, CCDC deposition numbers 2411860–2411867).

### Synthetic Procedures

In order to explore the influence of the synthetic route on the formation of double-complex salts of Co(III) and V(V), their syntheses were performed via conventional solution-based methods (under reflux or hydrothermally at 110 °C) and mechanochemically promoted by liquid-assisted ball milling followed by vapor-assisted aging.

All isolated compounds are insoluble in water and common organic solvents such as ethanol, acetone, or acetonitrile. They dissolve in DMF and DMSO with decomposition. The elemental analysis (C, H, N, Co, Na, Mo, and V), thermogravimetric, and PXRD analyses were taken as methods for determining sample purity and their identification. The PXRD patterns confirmed that the bulk powder is a single-phase material. The PXRD method was also used as a method of identification of the product obtained by different synthetic procedures.

#### Procedures for Synthesis of Compounds **1–4**

##### Solution-Based Methods: Hydrothermal Synthesis at 110 °C and under Reflux

Equal amounts of NH_4_VO_3_ (5 mmol) and CH_3_COOH or C_4_H_4_O_4_ (5 mmol) were dissolved in 10.0 mL of water. In the prepared aqueous solution, 10.0 mL of a [Co(ox)(NH_3_)_4_](NO_3_)·H_2_O (2 mmol) solution was added. The final reaction mixture was heated in a 30 mL Teflon-lined autoclave at 110 °C for 2 h. After cooling, the orange prisms of **1** and orange-yellow sticks of **2** were obtained in reaction with succinic acid. In reaction with acetic acid, the same mixture of **1** and **2** was isolated. The mixture of the crystalline products was filtered off, washed with a small amount of cold water, and dried in a desiccator. The products were separated mechanically (yield **1**:29.7 mg, 3.08%; yield **2**:29.0 mg, 2.57% in reaction with acetic acid; yield **1**:23.8 mg, 2.47%; yield **2**:14.2 mg, 1.12% in reaction with succinic acid). The same products were obtained in reactions performed under reflux. If the reaction was performed without the addition of acid the only product was **1**(yield: 35.21 mg, 3.65%).

##### Solid-State Method: Liquid-Assisted Ball Milling Followed by Vapor-Assisted Aging

The solids of NH_4_VO_3_ (5 mmol), CH_3_COOH or C_4_H_4_O_4_ (5 mmol) and [Co(ox)(NH_3_)_4_](NO_3_)·H_2_O (2 mmol) and acetone (25 μL) were placed in a 5 mL stainless steel jar. The reactants were milled for 30 min at a 25 Hz frequency. The produced rose solid reaction mixture was exposed to 100% humidity. After few days, a few crystals of **1** were observed and the transformation of rose solid to crystalline product **1** finished within 2 months (yield **1**: 24 mg; 2.49%; in reaction with acetic acid; yield **1**: 13.8 mg, 1.43%; in reaction with succinic acid; yield **1**: 28.7 mg, 2.98% in reaction without acid).

##### Solution-Based Methods: Hydrothermal Synthesis at 110 °C and under Reflux with Addition of Na_2_MoO_4_·2H_2_O

Equal amounts of NH_4_VO_3_ (5 mmol), Na_2_MoO_4_·2H_2_O (5 mmol), and CH_3_COOH or C_4_H_4_O_4_ (0.5 mmol) were dissolved in 10.0 mL of water. In the thus prepared aqueous solution of ammonium vanadate, 10.0 mL of [Co(ox)(NH_3_)_4_](NO_3_)·H_2_O (2 mmol) solution was added. The final reaction mixture was heated in a 30 mL Teflon-lined autoclave at 110 °C for 2 h. In the reaction with acetic acid, the mixture of products was obtained, orange prisms of **1**, orange plates of **3**, a few yellow-orange sticks of **4**, and several rose sticks of **11**.^29^ The mixture(s) of crystals was filtered off, washed with a small amount of cold water, and dried in a desiccator. The products were separated mechanically. After cooling the reaction mixture that contained acetic acid instead of succinic acid, the orange-orange prisms of **1** and orange-yellow sticks of **2** were obtained (yield **1**:63.7 mg, 6.61%; yield **3**:22.8 mg, 3.12%; yield **4**:0.5 mg, insufficient for analysis; yield **11**:43 mg, 1.69% in reaction with acetic acid yield **1**:62.8 mg; 6.51%; yield **3**:17.3 mg; 2.66% in reaction with succinic acid).

The same products were obtained in reactions conducted under the reflux (yield **1**:63.7 mg, 6.61%; yield **3**:22.8 mg; 3.51%; yield **4**:0.5 mg, insufficient for analysis; yield **11**:55.65 mg, 2.19% in reaction with acetic acid yield **1**:62.8 mg, 6.51%; yield **3**:17.3 mg, 2.66% in reaction with succinic acid).

If the reaction was performed without addition of acid, the only product was insoluble orange to red powder (yield: 35.2 or 45.1 mg).

##### Solid-State Method: Liquid-Assisted Ball Milling Followed by Vapor-Assisted Aging with Addition of Na_2_MoO_4_·2H_2_O

The solids of NH_4_VO_3_ (5 mmol), CH_3_COOH or C_4_H_4_O_4_ (5 mmol), [Co(ox)(NH_3_)_4_](NO_3_)·H_2_O (2 mmol), and acetone (25 μL) were placed in a 5 mL stainless steel jar. The reactants were milled for 30 min at 25 Hz frequency. The produced rose solid reaction mixture was exposed to 100% humidity. In both reactions (with acetic or succinic acid), a mixture of rose powder and crystals of **1** and **3** were obtained. After 10 days the crystals (of **1** and **3**) and rose powder transform to **4** and rose sticks of **11**.^29^ The crystals of **1**, **3**, **4**, and **11** were separated mechanically. If the reaction was performed without addition of acid, the only product was **11** (yield: 43.0 mg, 1.69%).

#### Procedures for the Synthesis of Compounds **5–9**

##### Solution-Based Methods: Hydrothermal Synthesis at 110 °C and under Reflux

Equal amounts of NH_4_VO_3_ (5 mmol) and CH_3_COOH or C_4_H_4_O_4_ (5 mmol) were dissolved in 10.0 mL of water. In the thus prepared aqueous solution of ammonium vanadate, 10.0 mL of a [Co(ox)(en)_2_]Cl·H_2_O (2 mmol) solution was added. The final reaction mixture was heated in a 30 mL Teflon-lined reactor at 110 °C for 2 h. After cooling mixture of the orange-red plates of **5** and dark orange sticks of **6** was obtained. The mixture was filtered off, washed with a small amount of cold water, and dried in a desiccator up to a constant weight. The crystalline products were separated mechanically (yield **5**:37.90 mg, 3.48%; yield **6**:40.25 mg, 2.83% in reaction with acetic acid; yield 5:31.50 mg, 2.81%; yield **6**:43.24 mg, 3.05% in reaction with succinic acid).

The same products were obtained in reactions performed under reflux (yield **5**:79.00 mg, 7.10%; yield **6**:59.10 mg, 4.15% in reaction with acetic acid; yield **5**:63.8 mg, 5.73%; yield **6**:44.35 mg, 3.12% in reaction with succinic acid).

If reactions were performed without adding acid, the only product was dark orange sticks of **6** (yield **6**:48.34 mg, 3.40%).

##### Solid-State Method: Liquid-Assisted Ball Milling Followed by Vapor-Assisted Aging

The solids of NH_4_VO_3_ (5 mmol), CH_3_COOH or C_4_H_4_O_4_ (5 mmol), [Co(ox)(en)_2_]Cl·H_2_O (2 mmol), and acetone (25 μL) were placed in a 5 mL stainless steel jar. The reactants were milled for 30 min at 25 Hz frequency. The produced rose powder was exposed to 100% humidity. After a few days, rose powder transformed into crystals of **6** and **7** (yield **6**: 43.78 mg, 6.78%; yield **7**: 33.20 mg, 3.77%; yield **6**: 57.10 mg, 4.01%; yield: **7**: 25.90 mg, 2.84%). The crystalline products were separated mechanically.

In reaction without acid, rose powder transforms to **9** (yield **9**: 24.00 mg, 1.83%).

##### Solution-Based Methods: Hydrothermal Synthesis at 110 °C and under Reflux with Addition of Na_2_MoO_4_

Equal amounts of NH_4_VO_3_ (5 mmol), Na_2_MoO_4_·2H_2_O (5 mmol), and CH_3_COOH or C_4_H_4_O_4_ (5 mmol) were dissolved in 10.0 mL of water. In the thus prepared aqueous solution of ammonium vanadate, 10.0 mL of [Co(ox)(en)_2_]Cl·H_2_O (2 mmol) solution was added. The final reaction mixture was heated in a 30 mL Teflon-lined reactor at 110 °C for 2 h. After cooling, the orange-red plates of **5** and dark orange prisms of **9** were obtained in a reaction with acetic acid. In a reaction performed in the presence of succinic acid after 7 days, crystals of **5** were isolated. After 21 days from the mother liquor, a mixture of **6** and **7** was obtained. The products were filtered off, washed with a small amount of cold water, and dried in a desiccator up to a constant weight. The crystalline products were separated mechanically (yield **5**: 46.20 mg, 4.15%; yield **9**: 42.20 mg, 3.22% in reaction with acetic acid; yield **5**: 56.90 mg, 5.12%; yield **6**: 48.50 mg, 3.12%; yield **7**: 25.90 mg, 2.84% in reaction with succinic acid).

Under reflux and in the presence of acetic acid, the resulting dark brown powder transformed after 20 days to the mixture of dark orange prisms of **9** and orange plates of **5**, while in reactions with succinic acid crystals of **6** were obtained. The crystalline products of **9** and **5** were separated mechanically (yield **5**: 79.20 mg, 7.12%; yield **9**: 71.00 mg, 5.42% in reaction with acetic acid; yield **6**: 83.80 mg, 5.90%; in reaction with succinic acid).

If the reaction was performed without addition of acid, the only product was 5 (yield 5:65.60 mg, 5.89%).

##### Solid-State Method: Liquid-Assisted Ball Milling Followed by Vapor-Assisted Aging with Addition of Na_2_MoO_4_

The solids of NH_4_VO_3_ (5 mmol), Na_2_MoO_4_·2H_2_O (5 mmol), CH_3_COOH or C_4_H_4_O_4_ (5 mmol), [Co(ox)(en)_2_]Cl·H_2_O (2 mmol), and acetone (25 μL) were placed in a 5 mL stainless steel jar. The reactants were milled for 30 min at 25 Hz frequency. The produced rose powder was exposed to 100% humidity. After few days, a mixture of orange sticks of **6** and prisms of **3** was obtained in reaction with acetic acid. The crystalline products of **6** and **3** were separated mechanically (yield **3**: 15.00 mg, 2.31%; yield **6**: 28.35 mg, 2.00% in reaction with acetic acid). In reaction with succinic acid, the first product was yellow sticks of [Co(ox)(en)_2_]Cl·4H_2_O,^46^ which disappeared after several hours. Dark orange sticks of **6** and several rose plates of **8** appeared after 3 days. The crystalline products of **6** and **8** were separated mechanically (yield **6**: 10.10 mg, 0.70% yield of **8**: <0.53 mg: insufficient for chemical and thermogravimetric analysis).

If the reaction was performed without adding acid, the only product was 9 (yield 9:14.00 mg, 1.07%.).

#### Procedures for the Synthesis of Compounds **10**

##### Solution-Based Methods: Hydrothermal Synthesis at 110 °C and under Reflux

Equal amounts of NH_4_VO_3_ (5 mmol) and CH_3_COOH or C_4_H_4_O_4_ (5 mmol) were dissolved in 10.0 mL of water and in solution was added solution of [Co(en)_3_](NO_3_)_3_·H_2_O (2 mmol in 10.0 mL). Upon mixing the solutions, the yellow voluminous product was precipitated instantaneously (yield: 76.08 mg).

Only in the reaction without acid, we were able to isolate a mixture of yellow powder and crystals of **10** (yield of **10**: 118.08 mg, 11.37%).

##### Liquid-Assisted Ball Milling Followed by Vapor-Assisted Aging

The solids of NH_4_VO_3_ (5 mmol), CH_3_COOH or C_4_H_4_O_4_ (5 mmol), [Co(ox)(en)_2_]Cl·H_2_O (2 mmol), and acetone (25 μL) were placed in a 5 mL stainless steel jar. The reactants were milled for 30 min at 25 Hz frequency. The produced yellow solid reaction mixture was exposed to 100% humidity. The final product after several days was a yellow powder (Yield: 24.40 mg, 2.35%).

Only in the reaction without acid, we were able to isolate a mixture of yellow powder and crystals of **10** (yield of **10**: 48.49 mg, 4.67%)

##### Solution-Based Method: Hydrothermal Synthesis at 110 °C and under Reflux with Addition of Na_2_MoO_4_

Equal amounts of NH_4_VO_3_ (5 mmol), Na_2_MoO_4_·2H_2_O (5 mmol), and CH_3_COOH or C_4_H_4_O_4_ (5 mmol) were dissolved in 10.0 mL of water. In the thus prepared aqueous solution of ammonium vanadate, 10.0 mL of a [Co(en)_3_](NO_3_)_3_·H_2_O (2 mmol) solution was added.

In all reactions, regardless of the added acid, the only product was insoluble gray-yellow powder.

Only in the reaction without acid, we were able to isolate a mixture of yellow powder and crystals of **10** (yield of **10**: 101.00 mg, 9.73%).

##### Liquid-Assisted Ball Milling Followed by Vapor-Assisted Aging with Addition of Na_2_MoO_4_·2H_2_O

The solids of NH_4_VO_3_ (5 mmol), Na_2_MoO_4_·2H_2_O (5 mmol), CH_3_COOH or C_4_H_4_O_4_ (5 mmol), and [Co(en)_3_](NO_3_)_3_·H_2_O (2 mmol) and acetone (25 μL) were placed in a 5 mL stainless steel jar. The reactants were milled for 30 min at 25 Hz frequency. The produced rose solid reaction mixture was exposed to 100% humidity. In all reactions with acids, the final product was a yellow powder; only in the reaction without acid, dark yellow sticks of Na_3_[Co(en)_3_][HMo_2_V_7_O_27_]·18H_2_O (**12**) and light-yellow sticks of **10** were obtained. The crystalline products **10** and **12** were separated mechanically (yield **10**: 22.14 mg, 2.13%; yield of **12**: 13.14 mg, 1.14%).

#### Reactions with [Co(NH_3_)_6_]Cl_3_ in the Presence or Absence of Na_2_MoO_4_·2H_2_O

In all reactions, regardless of the used method, added acid, or the presence of Na_2_MoO_4_·2H_2_O, the result was an insoluble mixture of differently colored powder products.

### Catalytic Procedure for Benzyl Alcohol Oxidation

A reaction mixture was prepared with benzyl alcohol (2.16 g, 20 mmol), biphenyl (0.1152 g, 0.75 mmol) as the internal standard, and catalysts **I**, **II**, **III** (0.1 mmol), and other catalysts (0.05 mmol). The mixture was heated to 80 °C prior to the addition of aqueous *tert*-butyl hydroperoxide (TBHP, 70% (w/w), 5.54 mL, 40 mmol). All measurements have been performed in triplicate and are in alignment with the typically accepted error deviations.

## Data Availability

The data supporting this study are provided throughout the manuscript. Raw data are not publicly available due to ongoing intellectual property considerations but may be obtained from the corresponding author upon reasonable request, subject to confidentiality agreements.

## Supporting Information Available

Additional experimental details, materials and methods, including IR spectra, thermograms, and PXRD patterns (PDF)

## Abbreviations

ox: oxalate anion
en: ethylenediamine
PXRD: powder X-ray diffraction
SCXRD: single-crystal X-ray diffraction
