# Supplementary material for: Cobalt Complex-Directed Self-Assembly of a Polyoxometalate-Based Species: Influence of Synthetic Methods on the Structure and Properties of Hybrid Assemblies
Source: ACS Omega. 2025 Apr 17;10(16):16668–82. doi: 10.1021/acsomega.5c00186 (PMC12044461; doi:10.1021/acsomega.5c00186)
Supplement: Supplementary file 1 — ao5c00186_si_009.pdf [file ao5c00186_si_009.pdf]

Cobalt Complex-Directed Self-Assembly of a Polyoxometalate-Based Species: Influence of Synthetic Methods on Structure and Properties of Hybrid Assemblies

Dino Kuzman[a]*, Mario Pajić[b], Lucija Drempetić[a], Josipa Sarjanović[a], Jana Pisk[a], Tomica Hrenar[a], Marina Cindrić[a]*, Višnja Vrdoljak[a]

[a]University of Zagreb, Faculty of Science, Department of Chemistry, Horvatovac 102a, Zagreb, Croatia

[b]Rudjer Boskovic Institute, Laboratory of Phisical Chemistry, Bijenička cesta 54, Zagreb, Croatia

Table S1. Reaction conditions and products for compounds **1-12**

Table S2. IR data of the compounds **1-8**, and **10-12** (crystals and yellow powder)

Table S3. Analytical data of compounds **1-3**, **5-7, 9-12** (for **10** crystals and yellow powder)

Table S4. Crystallographic data of the compounds **1-9**

Table S5. Hydrogen bonds in **1**

Table S6. Hydrogen bonds in **2**

Table S7. Hydrogen bonds in **5**

Table S8. Hydrogen bonds in **6**

Table S9. Hydrogen bonds in **7**

Table S10. Hydrogen bonds in **8**

Table S11. Hydrogen bonds in **9**

Figure S1. Asymmetric units of (a) 1, (b) 2, (c) 4, (d) 5, (e) 6, (f) 7, (g) 8 and (h) 9. Atoms are shown as thermal ellipsoids with 50% probability level

Figure S2: TG curve of $[Co(ox)(NH_3)_4]_4[H_2V_{10}O_{28}] \cdot 6H_2O$ (**1**)

Figure S3: TG curve of $[Co(ox)(NH_3)_4]_6[V_{10}O_{28}] \cdot 16H_2O$ (**2**)

Figure S4: TG curve of $[Na_2(H_2O)_{10}]n[[H_3V_{10}O_{28}Na(H_2O)_2\}]_n \cdot 3H_2O$ (**3**)

Figure S5: TG curve of $[Co(ox)(en)_2]_4[H_2V_{10}O_{28}]\cdot 12H_2O$ (**5**)

Figure S6: TG curve of $[Co(ox)(en)_2]_6[V_{10}O_{28}]\cdot nH_2O$ ($n \approx 13.5$)(**6**)

Figure S7: TG curve of $(H_2en)_2[Co(ox)(en)_2]_2[V_{10}O_{28}]\cdot nH_2O$ ($n \approx 2$) (**7**)

Figure S8: TG curve of $[Co(ox)(en)_2]_n[V_2O_6NaH_2O]_n\cdot nH_2O$ (**9**)

Figure S9: a) TG curve of crystals $[Co(en)_3]_n[V_3O_9]_n\cdot nH_2O$ (**10**)

b) TG curve of compound yellow powder of compound

Figure S10: TG curve of $[Co(ox)(NH_3)_4]_2[H_2Mo_8V_5O_{40}Na_2(H_2O)_8]\cdot5.5H_2O$ (**11**)

Figure S11: Comparasion of TG of compounds **10** and yellow powder obtained in reactions of $[Co(en)_3]Cl_3\cdot H_2O$ and NH_4VO_3 without acid and $[Co(en)_3]Cl_3\cdot H_2O$, $Na_2MoO_4\cdot2H_2O$ and NH_4VO_3 without acid

Figure S12: Comparasion of PXRD of compounds **10** and yellow powder obtained in reactions of $[Co(en)_3]Cl_3\cdot H_2O$ and NH_4VO_3 without acid and $[Co(en)_3]Cl_3\cdot H_2O$, $Na_2MoO_4\cdot2H_2O$ and NH_4VO_3 without acid

Figure S13: PXRD patterns of compounds **1** and **2**

Figure S14: PXRD patterns of compounds **3** and **4**

Figure S15: PXRD patterns of compounds **5** and **6**

Figure S16: PXRD patterns of compounds **7** and **8**

Figure S17: PXRD patterns of compound **9**

Figure S18: IR Spectra of compounds **1-11**

Figure S19. Comparasion of IR spectra of compounds **10** (red) and yellow powder (black)

Figure S20. Electrostatic potential mapped on the electron density isosurface (0.001 a.u.) for the optimized structure of $[Co(ox)(en)_2]_4[H_2V_{10}O_{28}]\cdot12H_2O$ (**5**) and $[Co(ox)(en)_2]_6[V_{10}O_{28}]\cdot nH_2O$ (n ≈ 2) (**6**)

Table S1. Reaction conditions and products (yellow: products obtained in reactions with $[Co(ox)(NH_3)_4]^+$ cation; grey: products obtained in reactions with $[Co(ox)(en)_2]^+$ cation; green: products obtained in reactions with $[Co(en)_3]^{3+}$ cation; products assigned red **4** and **11** are results of transformation of **1** and **3** during ageing; products assigned red **6** and **8** are finally products of transformation of mixture $[Co(ox)(en)_2]Cl·4H_2O^·$ and rose powder

	Hydrothermal 110 °C	Reflux	Mechanochemistry
Acetic acid	(1) $[Co(ox)(NH_3)_4]_4[H_2V_{10}O_{28}]·6H_2O$	(1) $[Co(ox)(NH_3)_4]_4[H_2V_{10}O_{28}]·6H_2O$	(1) $[Co(ox)(NH_3)_4]_4[H_2V_{10}O_{28}]·6H_2O$
	(2) $[Co(ox)(NH_3)_4]_6[V_{10}O_{28}]·16H_2O$	(2) $[Co(ox)(NH_3)_4]_6[V_{10}O_{28}]·16H_2O$	(3) $[Na_2(H_2O)_{10}]_n[H_3V_{10}O_{28}Na(H_2O)_2]_n·3nH_2O$
	(3) $[Na_2(H_2O)_{10}]_n[H_3V_{10}O_{28}Na(H_2O)_2]_n·3nH_2O$	(3) $[Na_2(H_2O)_{10}]_n[H_3V_{10}O_{28}Na(H_2O)_2]_n·3nH_2O$	(4) $[Co(ox)(NH_3)_4]_2[H_2V_{10}O_{28}Na(H_2O)_8·8H_2O$
	(4) $[Co(ox)(NH_3)_4]_2[H_2V_{10}O_{28}Na(H_2O)_8]·8H_2O$	(4) $[Co(ox)(NH_3)_4]_2[H_2V_{10}O_{28}Na(H_2O)_8]·8H_2O$	(11)$[Co(ox)(NH_3)_4]_2[H_2Mo_8V_5O_{40}Na_2(H_2O)_8]·5.5H_2O$
	(11)$[Co(ox)(NH_3)_4]_2[H_2Mo_8V_5O_{40}Na_2(H_2O)_8]·5.5H_2O$	(11)$[Co(ox)(NH_3)_4]_2[H_2Mo_8V_5O_{40}Na_2(H_2O)_8]·5.5H_2O$	(6) $[Co(ox)(en)_2]_6[V_{10}O_{28}]·16H_2O$
	(5) $[Co(ox)(en)_2]_4[H_2V_{10}O_{28}]$ $·12H_2O$	(5) $[Co(ox)(en)_2]_4[H_2V_{10}O_{28}]$ $·12H_2O$	(7) $(H_2en)_2[Co(ox)(en)_2]_2[V_{10}O_{28}]·8H_2O$
	(6) $[Co(ox)(en)_4]_6[V_{10}O_{28}]·16H_2O$	(6) $[Co(ox)(en)_4]_6[V_{10}O_{28}]·16H_2O$	
	(9) $[Co(ox)(en)_4]_n[V_2O_6Na(H_2O)]_n·nH_2O$	(9) $[Co(ox)(en)_4]_n[V_2O_6Na(H_2O)]_n·nH_2O$	
Succinic acid	(1) $[Co(ox)(NH_3)_4]_4[H_2V_{10}O_{28}]·6H_2O$	(1) $[Co(ox)(NH_3)_4]_4[H_2V_{10}O_{28}]·6H_2O$	(1) $[Co(ox)(NH_3)_4]_4[H_2V_{10}O_{28}]·6H_2O$
	(2) $[Co(ox)(NH_3)_4]_6[V_{10}O_{28}]·16H_2O$	(2) $[Co(ox)(NH_3)_4]_6[V_{10}O_{28}]·16H_2O$	(2) $[Co(ox)(NH_3)_4]_6[V_{10}O_{28}]·16H_2O$
	(5) $[Co(ox)(en)_2]_4[H_2V_{10}O_{28}]·12H_2O$	(5) $[Co(ox)(en)_2]_4[H_2V_{10}O_{28}]·12H_2O$	(3) $[Na_2(H_2O)_{10}]_n[H_3V_{10}O_{28}Na(H_2O)_2]_n·3nH_2O$
	(6) $[Co(ox)(en)_2]_6[V_{10}O_{28}]·16H_2O$	(6) $[Co(ox)(en)_2]_6[V_{10}O_{28}]·16H_2O$	(4) $[Co(ox)(NH_3)_4]_2[H_2V_{10}O_{28}Na(H_2O)_8]·8H_2O$
	(7) $(H_2en)_2[Co(ox)(en)_2]_2[V_{10}O_{28}]·8H_2O$		(11)$[Co(ox)(NH_3)_4]_2[H_2Mo_8V_5O_{40}Na_2(H_2O)_8]·5.5H_2O$
			* $[Co(ox)(en)_2]Cl·4H_2O$ + powder
			(6) $[Co(ox)(en)_2]_6[V_{10}O_{28}]·16H_2O$
			(8) $[Co(ox)(en)_2]_4[V_4O_{12}]$ $·14H_2O$ (8)
Without acid	(1) $[Co(ox)(NH_3)_4]_4[H_2V_{10}O_{28}]·6H_2O$	(1) $[Co(ox)(NH_3)_4]_4[H_2V_{10}O_{28}]·6H_2O$	(1) $[Co(ox)(NH_3)_4]_4[H_2V_{10}O_{28}]·6H_2O$
	(5) $[Co(ox)(en)_2]_4[H_2V_{10}O_{28}]$ $·12H_2O$	(5) $[Co(ox)(en)_2]_4[H_2V_{10}O_{28}]$ $·12H_2O$	(11)$[Co(ox)(NH_3)_4]_2[H_2Mo_8V_5O_{40}Na_2(H_2O)_8]·5.5H_2O$
	(6) $[Co(ox)(en)_2]_6[V_{10}O_{28}]·16H_2O$	(6) $[Co(ox)(en)_2]_6[V_{10}O_{28}]·16H_2O$	(9) $[Co(ox)(en)_4]_n[V_2O_6Na(H_2O)]_n·nH_2O$
	(10) $[Co(en)_3][V_3O_9]_n·nH_2O$	(10) $[Co(en)_3][V_3O_9]_n·nH_2O$	(10) $[Co(en)_3][V_3O_9]_n·nH_2O$
			(12) $Na_3[Co(en)_3][HMo_2V_7O_{27}]$ $·18H_2O$

Table S2. IR data of the compounds **1-12** and yellow powder

		**Experimental / ω(%)**	**Calculated / ω(%)**
**1. $[Co(ox)(NH_3)_4]_4[H_2V_{10}O_{28}]\cdot6H_2O$**		**C: 4.58; H: 3.69; N: 11.46; Co: 11.20; V: 24.89.**	**C: 4.98; H: 3.24; N: 11.63; Co: 12.23; V: 26.42.**
**2. $[Co(ox)(NH_3)_4]_6[V_{10}O_{28}]\cdot16H_2O$**		**C: 5.62; H: 4.15; N: 13.45; Co: 12.98; V: 19.22.**	**C: 5.68; H: 4.13; N: 13.26; Co: 13.94; V: 20.09.**
**3. $[Na_2(H_2O)_{10}]_n[H_3V_{10}NaO_{28}(H_2O)_2\}]_n\cdot3H_2O$**		**H: 3.49; Na: 4.70; V: 34.88.**	**H: 3.52; Na: 4.72; V: 34.85.**
**4. $[Co(ox)(NH_3)_4]_2[H_2V_{10}O_{28}Na(H_2O)_8]\cdot8H_2O$**	**Insufficient for analysis**		
**5. $[Co(ox)(en)_2]_4[H_2V_{10}O_{28}]\cdot12H_2O$**		**C: 12.52; H: 3.99; N: 9.76; Co: 10.54; V: 22.44.**	**C: 12.84; H: 4.04; N: 9.99; Co: 10.50; V: 22.72.**
**6. $[Co(ox)(en)_2]_6[V_{10}O_{28}]\cdot nH_2O$ ($n \approx 13.5$)**		**C: 15.12; H: 4.45; N: 11.45; Co: 12.33; V: 17.84.**	**C: 15.42; H: 4.42; N: 11.99; Co: 12.61; V: 18.17.**
**7. $(H_2en)_2[Co(ox)(en)_2]_2[V_{10}O_{28}]\cdot nH_2O$ ($n \approx 2$)**		**C: 10.66; H: 4.01; N: 9.45; Co: 6.38; V: 28.24.**	**C: 11.63; H: 3.42; N:10.17; Co: 7.13; V: 30.84**
**8. $[Co(ox)(en)_2]_4[V_4O_{12}]\cdot14H_2O$**	**Insufficient for analysis**		
**9. $[Co(ox)(en)_2]_n[V_2O_6Na(H_2O)]_n\cdot nH_2O$**		**C: 14.08; H: 3.78; N: 10.99: Co: 13.84; Na:4.60; V: 19.94.**	**C: 13.75; H: 3.85; N: 10.69; Co: 11.25; Na: 4.39; V: 19.44.**
**10. $[Co(en)_3]_n[V_3O_9]_n\cdot nH_2O$**		**C: 12.76; H: 4.54; N: 15.05; Co: 10.24; V: 27.00.**	**C: 13.01; H: 4.73; N: 15.17; Co: 10.64; V: 27.58.**
**11. $[Co(ox)(NH_3)_4]_3[H_2Mo_8V_5O_{40}Na_2(H_2O)_8]\cdot5.5H_2O$**		**C: 2.08; H; 1.63; N: 4.98; Na: 1.22; Co: 4.96; Mo: 21.53 ; V: 35.55**	**C: 1.98; H; 1.55; N: 4.68; Na: 1.21; Co: 4.66; Mo: 21.29; V: 35.45**
**12. $Na_2[Co(en)_3][HMo_2V_7O_{27}]\cdot nH_2O$**		**C: 4.40; H; 3.62; N: 5.11; Na: 4.18; Co: 3.33; Mo: 11.45 ; V: 22.05**	**C: 4.47; H; 3.75; N: 5.21; Na4.28; Co: 3.65; Mo: 11.90; V: 22.11**
**Yelow powder**		**C: 12.98; H; 4.33; N: 14.98; Co: 10.51; V: 27.16.**	**C: 13.01; H: 4.73; N: 15.17; Co: 10.64; V: 27.58.**

Table S3. Analytical data of compounds **1-3**, **5-7, 9-11** and for yellow powder

	ṽ (N–H) + ṽ (C–H) + ṽ (O–H iz H_2O)	ṽ (C–O) + δ(NH)	ṽ (C–O)	ṽ (C–O) + ṽ (C–C)	ṽ ($M–O_t$) (M = V or Mo)	$ṽ_{as}$ $M–O_b$ + ṽ (C–C) + δ(O–C–O) (M = V or Mo)	$ṽ_s$ ($M–O_b$) (M = V or Mo)
1. $[Co(ox)(NH_3)_4]_4[H_2V_{10}O_{28}]·6H_2O$	3187m, b	1670m, 1651vs,	1399vs, 1292s	1260m	952vs	814vs, 744m	560m
2. $[Co(ox)(NH_3)_4]_6[V_{10}O_{28}]·16H_2O$	3281m,b	1701sh, 1669bs	1399b,s, 1316m	1262m	930s	783s	657s
3. $[Na_2(H_2O)_{10}]_n[H_3V_{10}NaO_{28}(H_2O)_2\}]_n·nH_2O$	3458-3129m,b	1699s, 1660vs, 1575vs	1457m, 1391vs	1257w, 1225w,	1054s, 940vs,	799s, 740m	586s
4. $[Co(ox)(NH_3)_4]_2[H_2V_{10}O_{28}Na(H_2O)_8]·8H_2O$	3544-3223m,b	1677s, 1634vs, b	1455m, 1409s	1234m, 1212m	998s, 976s	782s, 755s	589s
5. $[Co(ox)(en)_2]_4[H_2V_{10}O_{28}]·12H_2O$	3476-3134m, b	1699m, 1658vs, 1578s	1458w, 1388s	1286w, 1262w,	1055m, 943s, 899m	805s, 727m	588s
6. $[Co(ox)(en)_2]_6[V_{10}O_{28}]·13.5H_2O$ (n ≈ 13.5)	3433-3127m, b	1709sh, 1668vs, 1569m	1459w, 1407s	1270w, 1231w	1057m, 937vs, 927vs	805vs, 732vs	588sh, 514s
7. $(H_2en)_2[Co(ox)(en)_2]_2[V_{10}O_{28}]·nH_2O$ (n ≈ 2)	3475-3129m, b	1700m, 1666s, 1574s	1459w, 1408m	1268w, 1228w	1057m, 937vs	801vs, 725vs	586s
8. $[Co(ox)(en)_2]_4[V_4O_{12}]·14H_2O$	3467-3128 m, b	1699m, 1656vs, 1577s	1460w, 1420s	1287w, 1266w	945s, 899m	804s, 734sb	578s
9. $[Co(ox)(en)_2]_n[V_2O_6Na(H_2O)]_n·H_2O$	3234-3130 m	1711s, 1657vs, 1575s	1468w, 1388vs	1289w, 1260w	1054m, 946vs, 895m	810vs, 728s	669w, 559m
10. $[Co(en)_3]_n[V_3O_9]_n·nH_2O$	30465-3039m,b	1577m	1459m, 1369m	1280m	1052m, 888vs	789vs, 738vs	621b,s, 580bs
11. $[Co(ox)(NH_3)_4]_3[H_2Mo_8V_5O_{40}Na_2(H_2O)_8]·5.5H_2O$	3125b	1698m,1672m	1409s, 1311m	1264m	938s, 874s	825m,803m	687m, 648m
12. $Na_2[Co(en)_3][HMo_2V_7O_{27}]·nH_2O$	3230-3125b	1587m	1324s	1222m	945s,769s	813m	665m, 627m
Yellow powder	30465m,b	1577m	1449m, 1320m	1276m	1012m, 876vs	790vs, 743vs	622b,s, 567b

Table S4. Crystallographic data of the compounds **1, 2, 4-9**, **11** and **12**.

	**1**	**2**	**4**
Formula	$C_8H_{62}Co_4N_{16}O_{50}V_{10}$	$C_{12}H_{104}Co_6N_{24}O_{68}V_{10}$	$C_4H_{40}Co_2N_8Na_2O_{52}V_{10}$
M_r	1927.85	2536.17	1705.68
Crystal system	triclinic	triclinic	monoclinic
Space group	*P*-1	*P*-1	*I*2/*m*
a (Å)	9.2270(4)	11.0016(10)	12.6140(6)
b (Å)	12.5720(6)	11.1678(8)	8.5700(4)
c (Å)	12.7821(7)	16.3643(9)	23.3213(12)
α (°)	78.466(4)	101.725(5)	90
β (°)	73.735(4)	98.683(7)	90.845(5)
γ (°)	72.307(4)	98.011(7)	90
V (Å^3)	1345.34(12)	1915.8(3)	2520.8(2)
Z	1	1	2
Reflections unique	16664	23116	15054
Reflections observed [$I > 2\ \sigma(I)$]	5452	7667	2776
Parameters	414	565	205
R_1(obs)	0.0378	0.1037	0.0701
wR_1(obs)	0.0996	0.2693	0.2064
GooF	1.036	1.059	1.071

	**5**	**6**
Formula	$C_{24}H_{90}Co_4N_{16}O_{56}V_{10}$	$C_{36}H_{128}Co_6N_{24}O_{68}V_{10}$
M_r	2242.22	2848.59
Crystal system	monoclinic	triclinic
Space group	*Pc*	*P*-1
a (Å)	13.3783(3)	14.3839(2)
b (Å)	12.2659(3)	18.4717(3)
c (Å)	22.1258(5)	19.9541(2)
α (°)	90	107.0810(10)
β (°)	100.514(2)	102.6030(10)
γ (°)	90	90.5690(10)
V (Å^3)	3569.82(15)	4930.15(12)
Z	2	2
Reflections unique	26341	157506
Reflections observed [$I > 2\ \sigma(I)$]	11115	20336
Parameters	1028	1340
R_1(obs)	0.0558	0.0817
wR_1(obs)	0.1481	0.2315
GooF	1.041	1.071

	**7**	**8**	**9**
Formula	$C_{16}H_{52}Co_2N_{12}O_{46}V_{10}$	$C_{24}H_{76}Co_4N_{16}O_{44}V_4$	$C_6H_{20}CoN_4NaO_{12}V_2$
M_r	1775.95	1732.48	524.05
Crystal system	monoclinic	triclinic	monoclinic
Space group	$P2_1/n$	P-1	P2$_1$/n
a (Å)	13.4331(4)	11.7312(11)	5.8708(2)
b (Å)	13.1574(3)	12.1480(7)	15.7267(7)
c (Å)	16.4034(6)	12.7010(7)	19.1592(7)
α (°)	90	90.115(5)	90
β (°)	97.131(3)	103.811(7)	92.578(4)
γ (°)	90	110.203(7)	90
V (Å^3)	2876.79(15)	1642.5(2)	1767.15(12)
Z	2	1	4
Reflections unique	22498	19909	19258
Reflections observed [$I > 2\ \sigma(I)$]	5810	6467	5913
Parameters	410	424	257
R_1(obs)	0.0596	0.0757	0.0636
wR_1(obs)	0.1664	0.2010	0.1227
GooF	1.079	1.066	1.011

	**11**	**12**
Formula	$C_6H_{57}Co_3Mo_8N_{12}Na_2O_{62.5}V_5$	$C_6H_{60}CoMo_2N_6Na_3O_{45}V_7$
M_r	2542.63	1612.95
Crystal system	triclinic	monoclinic
Space group	P-1	P-1
a (Å)	13.2251(3)	12.1225(2)
b (Å)	15.9667(3)	13.4878(2)
c (Å)	16.2567(5)	14.4274(2)
α (°)	86.364(2)	92.9430(10)
β (°)	81.442(2)	97.9090(10)
γ (°)	88.936(2)	91.0120(10)
V (Å^3)	3387.59(15)	2332.71(6)
Z	2	2
Reflections unique	44397	36368
Reflections observed [$I > 2\ \sigma(I)$]	13752	9832
Parameters	850	629
R_1(obs)	0.0979	0.0751
wR_1(obs)	0.2617	0.2170
GooF	1.045	1.058

Table S5. Hydrogen bonds in **1**

Hydrogen bonds formed between complex cations	
Donor–H···Acceptor	H-bond lenght / Å
N1–H1A···O19	3.049(3)
N1–H1B···O17	3.248(5)
N1–H1C···O16	3.218(5)
N2–H2A···O21	2.990(3)
N2–H2A···O25	3.101(4)
N2–H2B···O18	3.087(4)
N3–H3A···O25	3.137(4)
N3–H3B···O17	3.146(4)
N3–H3C···O19	3.113(4)
N4–H4C···O25	3.108(3)
N5–H5B···O19	2.975(4)
N5–H5C···O17	3.038(4)
N6–H6B···O17	3.184(4)
N6–H6B···O18	3.193(4)
N6–H6C···O20	3.134(4)
N7–H7B···O24	2.927(5)
N7–H7C···O25	3.256(4)
N8–H8A···O24	3.085(5)
N8–H8B···O20	3.256(4)
N8–H8B···O22	3.071(3)
N8–H8C···O18	3.087(3)
Hydrogen bonds formed between complex cations and anions	
N1–H1C···O3	3.064(3)
N2–H2C···O3	3.071(3)
N3–H3A···O4	3.140(4)
N3–H3B···O1	3.153(4)
N4–H4A···O7	3.145(3)
N4–H4B···O11	3.167(3)
N4–H4C···O4	3.278(4)
N4–H4C···O5	3.352(4)
N4–H4C···O9	2.985(3)
N5–H5A···O12	3.049(4)
N5–H5A···O10	3.356(3)
N6–H6A···O12	3.047(4)
N6–H6A···O13	3.279(3)
N7–H7A···O10	2.981(4)
N8–H8C···O1	3.012(3)

Table S6. Hydrogen bonds in **2**

Hydrogen bonds formed between complex cations	
Donor–H···Acceptor	H-bond lenght / Å
N1–H1A···O21	3.133(11)
N3–H3A···O25	3.001(13)
N5–H5A···O23	3.448(12)
N5–H5A···O25	3.249(13)
N5–H5B···O18	3.134(11)
N5–H5C···O17	3.161(11)
N5–H5C···O25	3.035(13)
N6–H6B···O26	2.914(13)
N6–H6C···O16	3.336(10)
N8–H8B···O16	3.095(10)
N8–H8C···O23	3.097(11)
N9–H9A···O15	3.042(11)
N9–H9C···O19	3.384(14)
N10–H10A···O22	2.942(13)
N10–H10B···O22	3.266(13)
N12–H12A···O18	3.088(11)
N12–H12B···O17	3.115(12)
N12–H12C···O19	3.354(12)
Hydrogen bonds formed between complex cations and anions	
N1–H1C···O7	3.336(10)
N1–H1C···O13	2.981(10)
N2–H2B···O9	2.932(10)
N3–H3C···O8	3.131(10)
N3–H3C···O2	3.005(11)
N4–H4B···O7	2.961(10)
N4–H4C···O2	3.228(10)
N4–H4C···O5	3.136(9)
N6–H6A···O1	3.048(10)
N6–H6A···O3	3.156(10)
N7–H7B···O13	2.909(11)
N7–H7C···O11	2.994(11)
N8–H8C···O10	2.995(10)
N9–H9B···O10	3.023(11)
N11–H11B···O8	3.031(10)

Table S7. Hydrogen bonds in **5**

Hydrogen bonds formed between complex cations	
Donor–H···Acceptor	H-bond lenght / Å
N3–H3A···O17	2.891(6)
N5–H5A···O16	3.162(6)
N5–H5A···O18	3.130(6)
N5–H5B···O21	2.829(6)
N6–H6A···O17	2.919(6)
N7–H7B···O18	3.013(6)
N7–H7B···O19	3.244(5)
N8–H8A···O18	3.094(6)
Hydrogen bonds formed between complex cations and anions	
N1–H1B···O13	3.199(6)
N1–H1B···O8	2.923(5)
N1–H1B···O9	3.172(6)
N2–H2A···O9	3.210(6)
N2–H2B···O14	2.885(6)
N3–H3A···O13	3.039(6)
N3–H3B···O14	3.027(6)
N4–H4B···O6	3.162(6)
N4–H4B···O11	2.961(6)
N6–H6B···O2	3.006(6)
N6–H6B···O14	3.319(6)

Table S8. Hydrogen bonds in **6**

Hydrogen bonds formed between complex cations	
Donor–H···Acceptor	H-bond lenght / Å
N10–H10C···O51	2.919(11)
N10–H10D···O31	2.948(10)
N10–H10D···O32	3.150(10)
N11–H11B···O32	2.968(8)
N12–H12A···O52	2.976(13)
N12–H12B···O43	2.948(8)
N12–H12B···O44	2.983(10)
N14–H14D···O36	3.031(10)
N15–H15D···O34	3.282(9)
N16–H16C···O46	3.346(9)
N16–H16C···O48	3.179(9)
N16–H16D···O40	2.963(9)
N17–H17B···O40	3.103(10)
N18–H18B···O31	3.008(7)
N19–H19B···O40	3.101(9)
N20–H20B···O31	2.992(9)
N21–H21C···O36	2.956(9)
N21–H21D···O47	2.945(8)
N21–H21D···O48	3.058(9)
N23–H23B···O35	2.889(10)
N24–H24B···O43	3.036(12)
Hydrogen bonds formed between complex cations and anions	
N1–H1B···O1	3.067(7)
N1–H1B···O10	3.072(7)
N2–H2A···O20	2.980(7)
N2–H2B···O22	2.940(7)
N2–H2B···O20	2.980(7)
N3–H3B···O2	2.968(7)
N4–H4B···O27	2.876(7)
N5–H5A···O25	2.935(7)
N6–H6A···O9	2.915(7)
N7–H7A···O21	3.032(7)
N7–H7A···O23	3.091(7)
N8–H8A···O5	2.928(7)
N8–H8A···O6	2.971(7)
N9–H9C···O7	3.008(8)
N9–H9C···O14	3.014(9)
N11–H11A···O4	3.232(7)
N11–H11A···O6	3.071(7)
N11–H11A···O7	3.190(8)
N13–H13C···O24	2.922(8)
N15–H15C···O25	2.710(8)
N15–H15C···O26	3.403(10)
N18–H18A···O2	2.940(7)
N19–H19A···O13	3.074(7)
N19–H19A···O4	3.306(8)

Table S9. Hydrogen bonds in **7**

Hydrogen bonds formed between complex cations	
Donor–H···Acceptor	H-bond lenght / Å
N2–H2A···O15	3.317(7)
N2–H2A···O17	3.283(7)
N4–H4A···O17	2.894(8)
Hydrogen bonds formed between cations and anions	
N1–H1B···O9	3.031(7)
N1–H1B···O10	3.064(8)
N2–H2B···O7	3.268(8)
N2–H2B···O8	2.825(7)
N2–H2B···O3	3.325(8)
N3–H3B···O7	2.983(8)
N4–H4B···O8	3.001(7)
N4–H4B···O2	3.109(8)
N5–H5A···O2	2.781(9)
N6–H6C···O3	2.751(10)

Table S10. Hydrogen bonds in **8**

Hydrogen bonds formed between complex cations	
Donor–H···Acceptor	H-bond lenght / Å
N2–H2A···O15	3.317(7)
N2–H2A···O17	3.283(7)
N4–H4A···O17	2.894(8)
Hydrogen bonds formed between cations and anions	
N1–H1B···O9	3.031(7)
N1–H1B···O10	3.064(8)
N2–H2B···O7	3.268(8)
N2–H2B···O8	2.825(7)
N2–H2B···O3	3.325(8)
N3–H3B···O7	2.983(8)
N4–H4B···O8	3.001(7)
N4–H4B···O2	3.109(8)
N5–H5A···O2	2.781(9)
N6–H6C···O3	2.751(10)

Table S11. Hydrogen bonds in **9**

Hydrogen bonds formed between complex cations	
Donor–H···Acceptor	H-bond lenght / Å
N1–H1A···O13	3.295(10)
N2–H2B···O12	3.026(9)
N3–H3A···O14	2.931(9)
N3–H3B···O14	2.903(9)
N4–H4A···O7	3.016(8)
N4–H4A···O9	3.180(9)
N5–H5A···O11	3.133(9)
N5–H5A···O13	3.213(10)
N6–H6A···O9	3.021(9)
N6–H6A···O10	2.960(9)
N6–H6B···O10	2.937(9)
N7–H7A···O13	3.119(9)
N7–H7B···O8	2.984(8)
N7–H7B···O10	3.220(10)
N8–H8A···O9	2.940(11)
Hydrogen bonds formed between cations and anions	
N2–H2A···O2	3.050(9)
N4–H4B···O2	3.027(10)

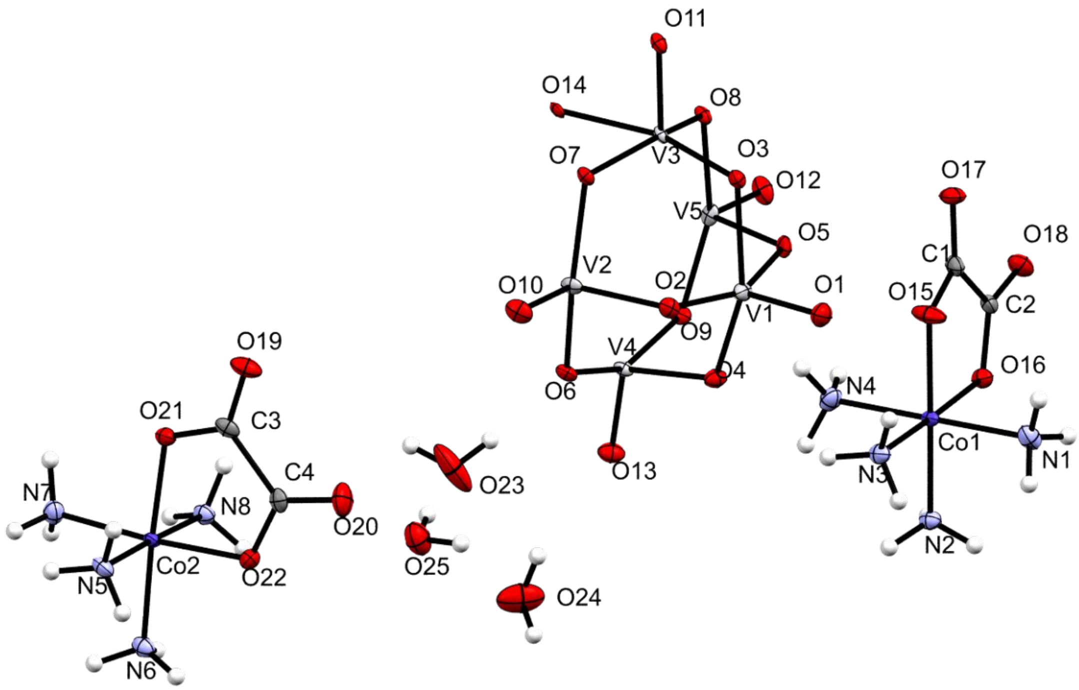
O11
O14
O8
O7
V3
O3
O12
V5
O5
O17
O18
C1
C2
O10
V2
O2
O9
V1
O1
O15
O19
V4
O6
O4
O16
N4
O21
C3
Co1
N1
N3
O13
C4
O23
N7
N8
O20
N2
Co2
O22
O25
N5
O24
N6

(a)

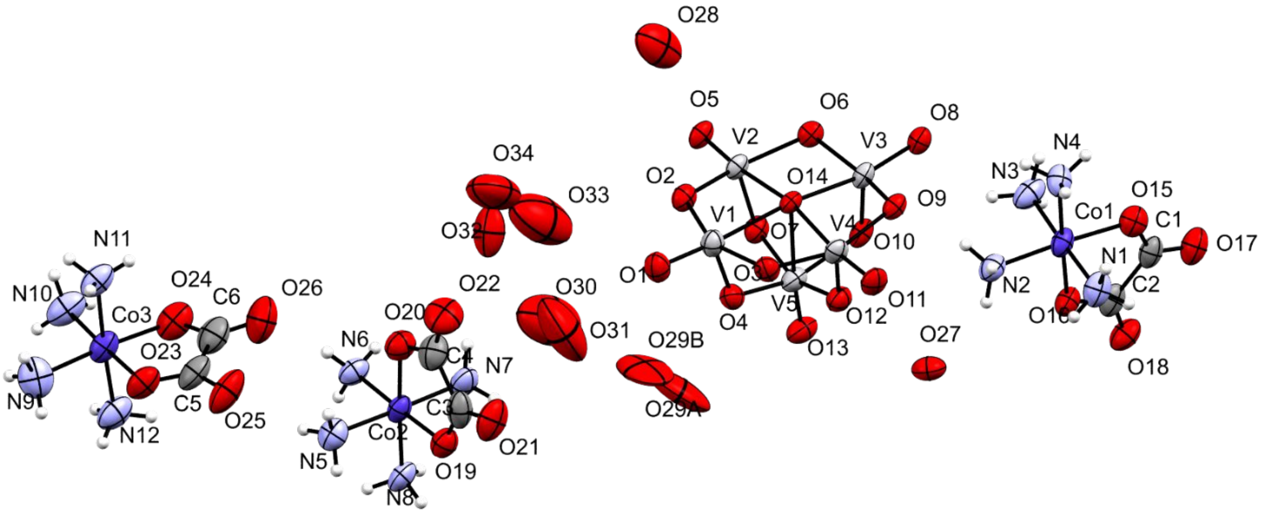
O28
O5
O6
O8
O34
V2
V3
N4
N3
O2
O14
O33
O9
O15
O32
V1
O7
V4
Co1
C1
O17
N11
O10
O3
N1
N10
O24
C6
O26
O22
O30
O11
N2
C2
Co3
O20
V5
O12
O23
O31
O29B
O4
O27
N6
N7
O13
O18
C5
C4
N9
O25
C3
O29A
N12
Co2
O21
N5
O19
N8

(b)

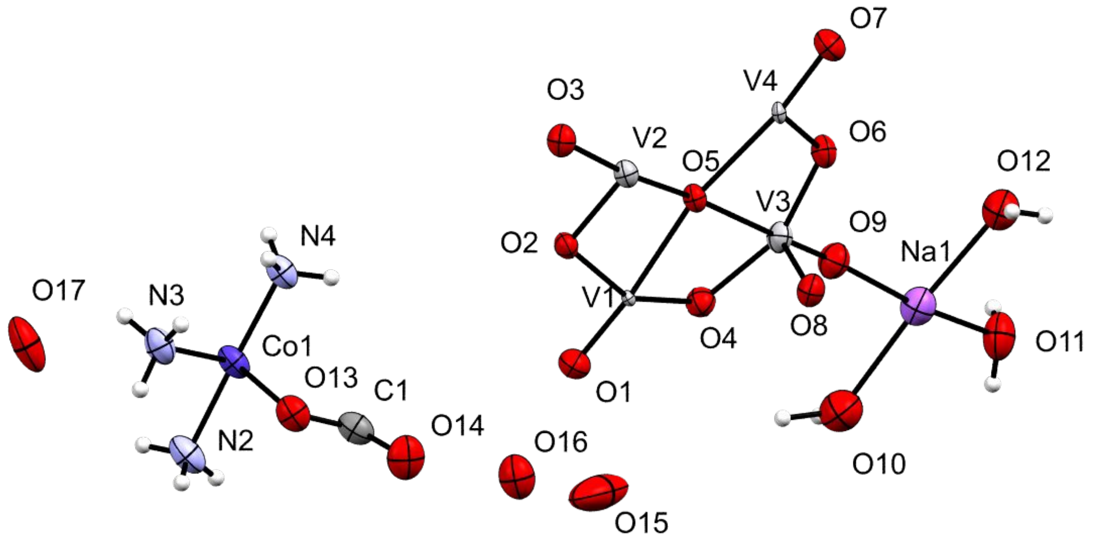
O7
V4
O3
V2
O5
O6
O12
V3
O9
Na1
O2
N4
O17
N3
V1
O4
O8
O11
Co1
O13
C1
O1
N2
O14
O16
O10
O15

(c)

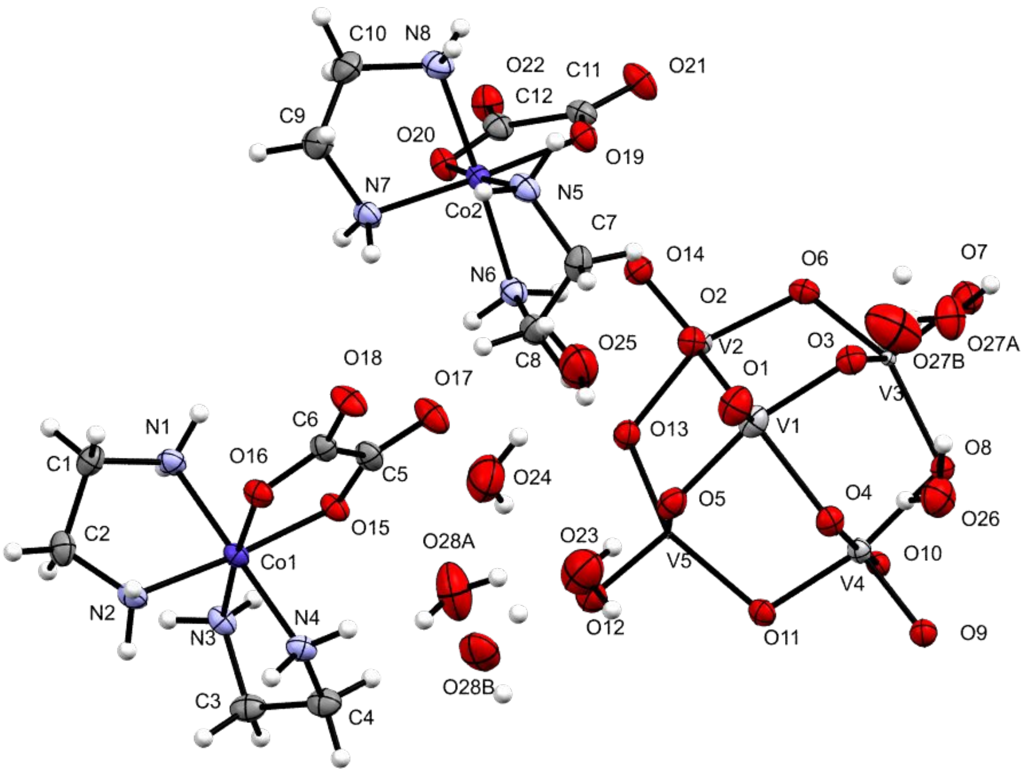
C10
N8
O22
C11
O21
C9
C12
O20
O19
N7
N5
Co2
C7
O14
O6
O7
N6
O2
V2
O3
O27B
O27A
O25
C8
O18
O17
O1
V3
N1
V1
C6
O13
O8
C1
O16
C5
O24
O5
O4
O26
C2
O15
O28A
O23
Co1
V5
V4
O10
N2
N3
N4
O12
O11
O9
C3
C4
O28B

(d)

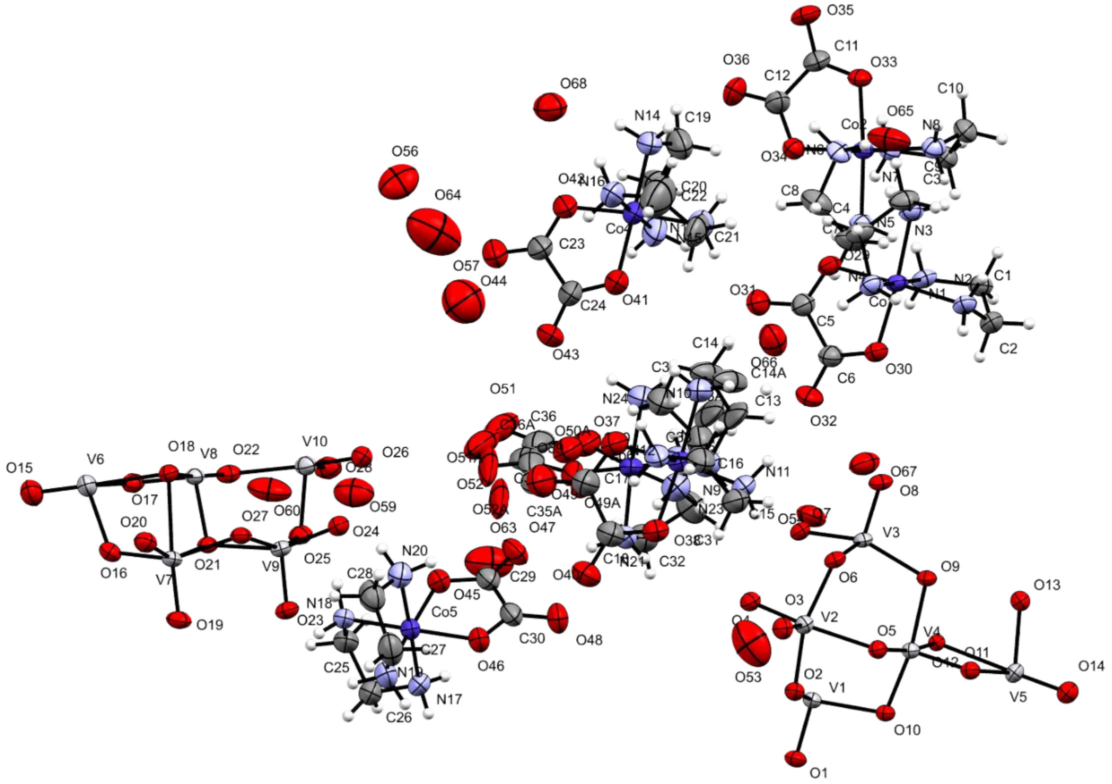
O35
O36
C11
C12
O33
C10
O68
N14
C19
O65
N8
Co2
O56
O34
N7
O64
N16
C20
C22
C8
C4
N5
N3
Co4
C21
C23
O57
O44
C24
O41
O31
N2
C1
N1
C5
C2
O43
C14
O66
C14A
C13
O30
C6
O32
C36
O37
O51
N24
C16
N11
N9
O67
O8
C17
C15
O15
V6
O18
V8
O22
V10
O26
O17
O59
O20
O27
O60
O24
C35A
O47
O49A
N23
N21
C32
V3
O63
O6
O9
O16
V7
O21
V9
O25
N20
C28
O45
C29
O13
O3
V2
O19
N18
O23
Co5
C27
O46
C30
O48
O5
V4
O11
O14
C25
N17
C26
O2
V1
O10
V5
O53
O1

(e)

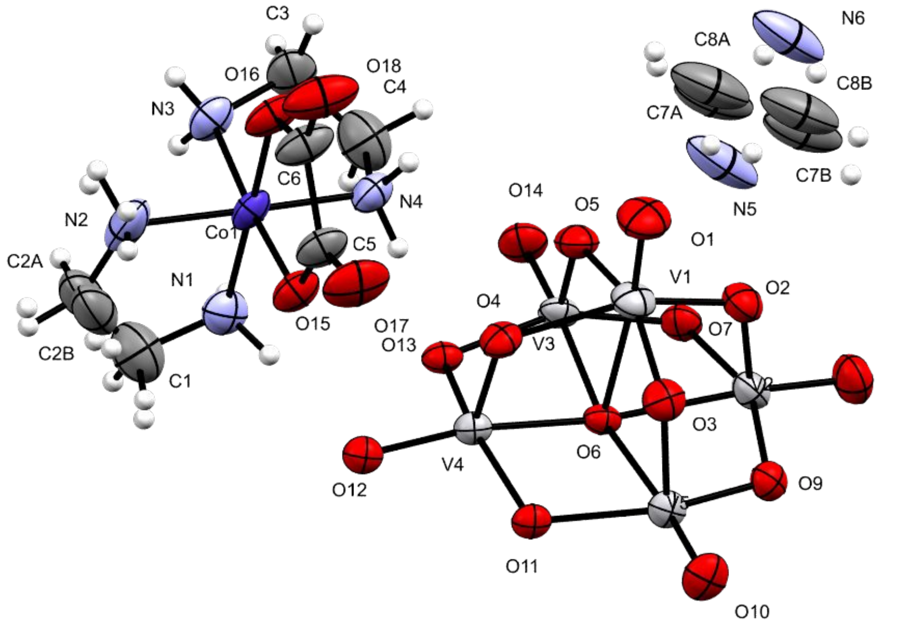
C3
O16
O18
C4
N6
C8A
C8B
C7A
C7B
N3
C6
N4
N5
N2
Co1
C5
O14
O5
O1
C2A
N1
V1
O2
O4
O7
O17
V3
O13
C2B
C1
O8
V2
O3
O6
O12
V4
O9
V5
O11
O10

(f)

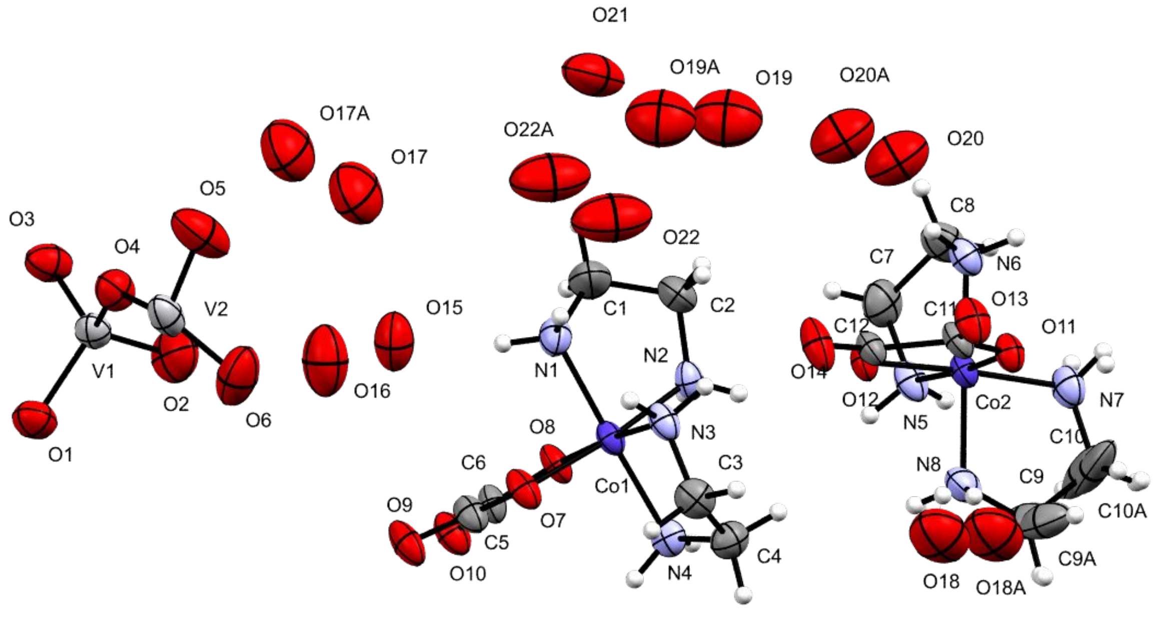

(g)

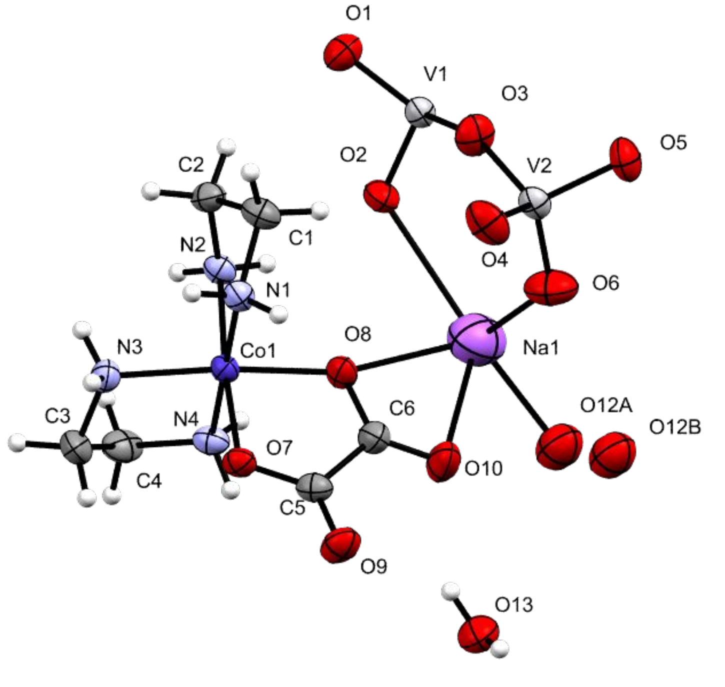

(h)

Figure S1. Asymmetric units of (a) 1, (b) 2, (c) 4, (d) 5, (e) 6, (f) 7, (g) 8 and (h) 9. Atoms are shown as thermal ellipsoids with 50% probability level

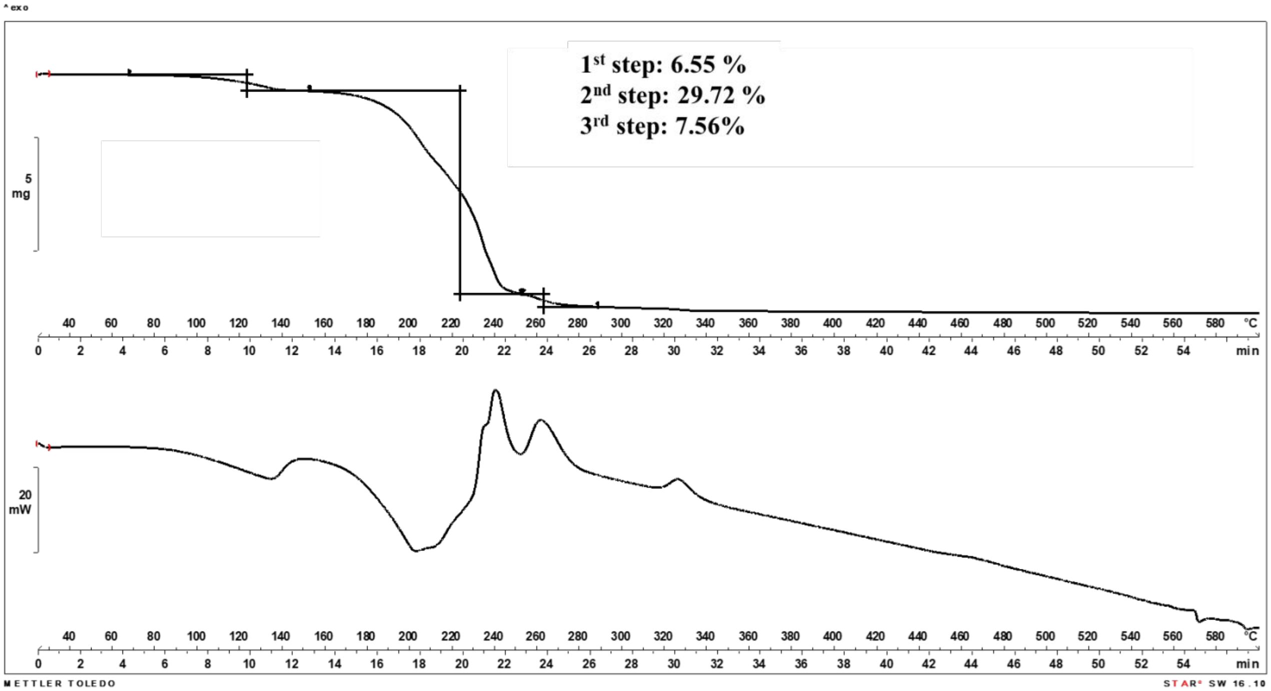

Figure S2. TG curve of $[Co(ox)(NH_3)_4]_4[H_2V_{10}O_{28}] \cdot 6H_2O$ (**1**)

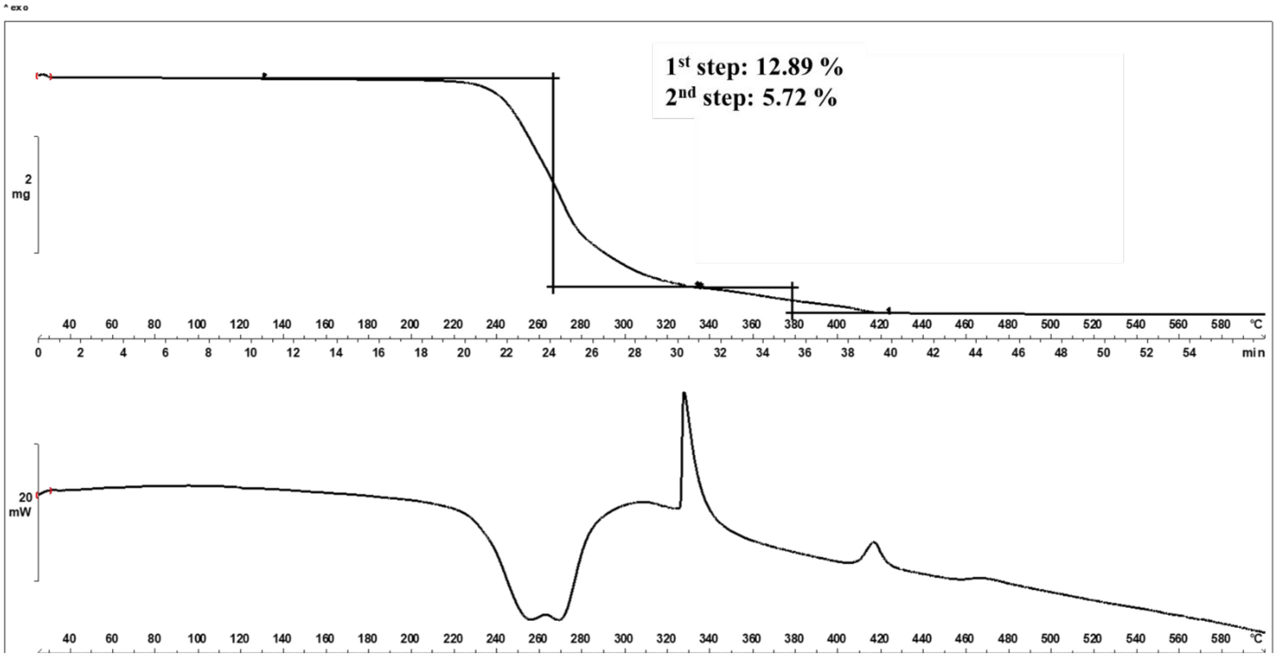

Figure S3. TG curve of $[Co(ox)(NH_3)_4]_6[V_{10}O_{28}] \cdot 16H_2O$ (**2**)

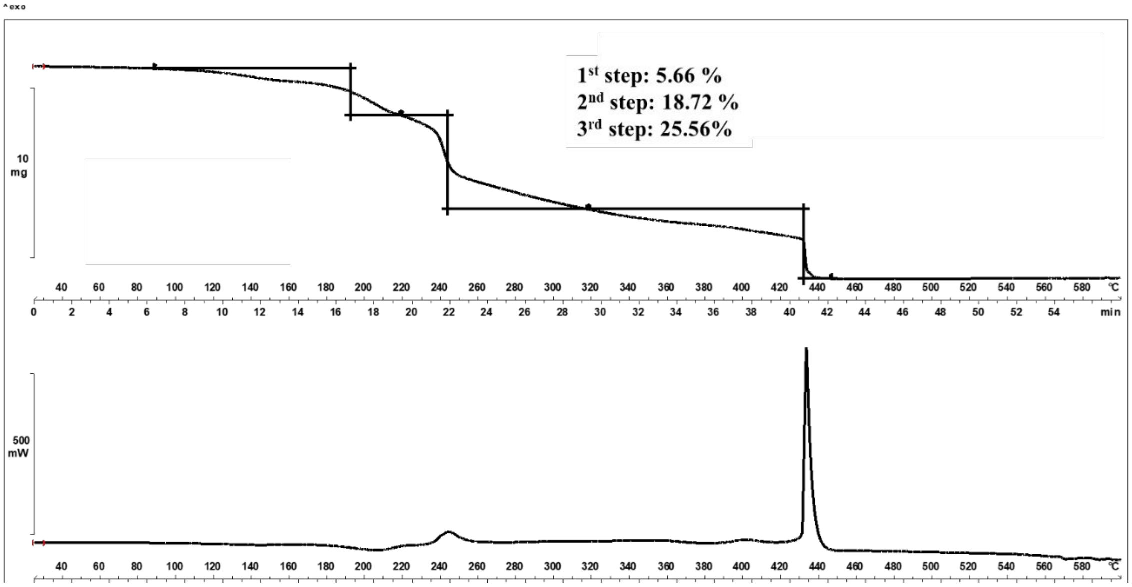

Figure S4. TG curve of $[Na_2(H_2O)_{10}][H_3V_{10}O_{28}Na(H_2O)_2]_n \cdot 3nH_2O$ (**3**)

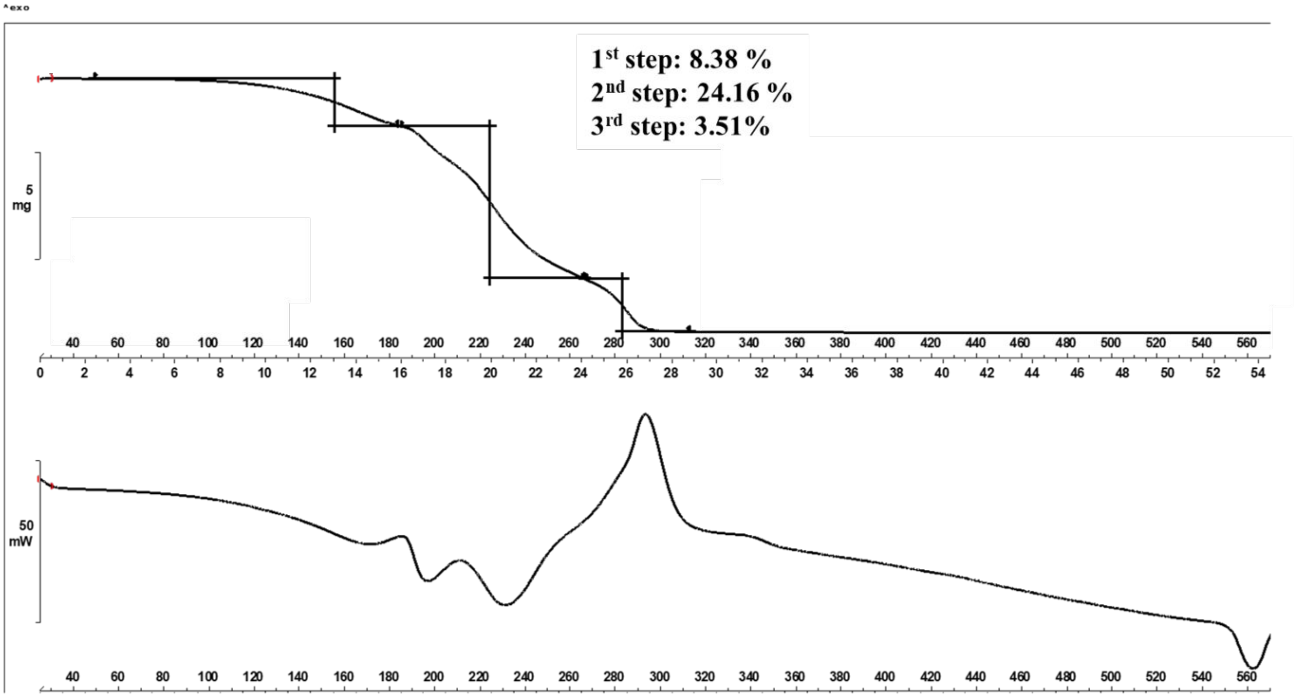

Figure S5. TG curve of $[Co(ox)(en)_2]_4[H_2V_{10}O_{28}]\cdot 12H_2O$ (**5**)

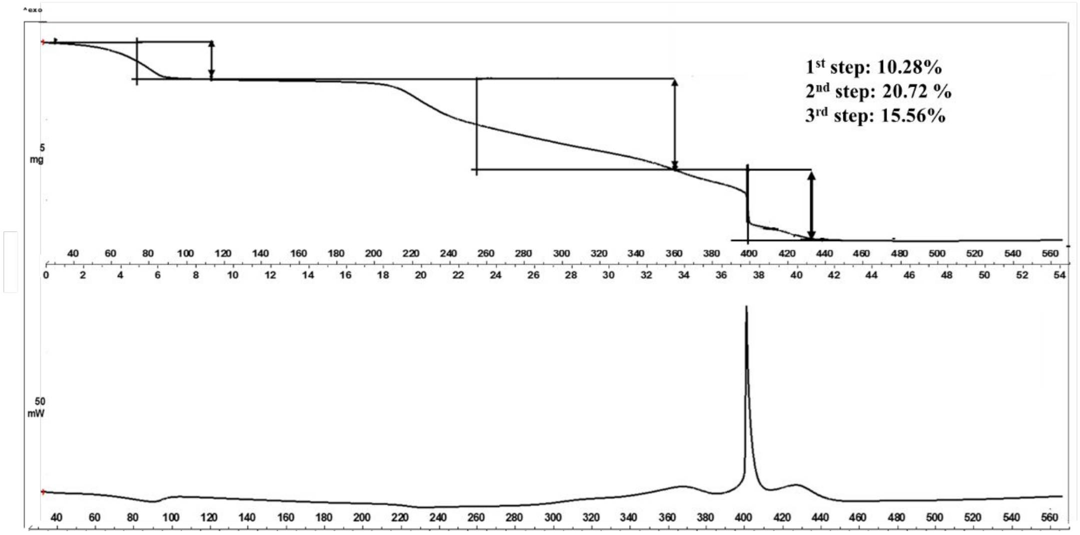

Figure S6. TG curve of $[Co(ox)(en)_2]_6[V_{10}O_{28}]\cdot 16H_2O$ (**6**)

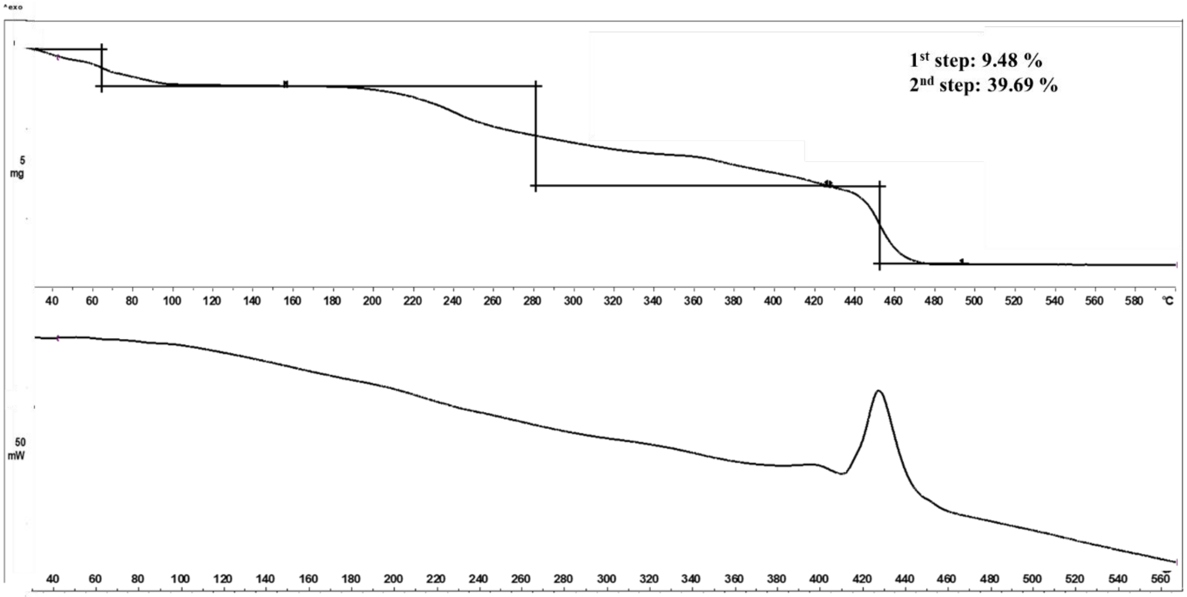

Figure S7. TG curve of $(H_2en)_2[Co(ox)(en)_2]_2[V_{10}O_{28}]\cdot 8H_2O$ (**7**)

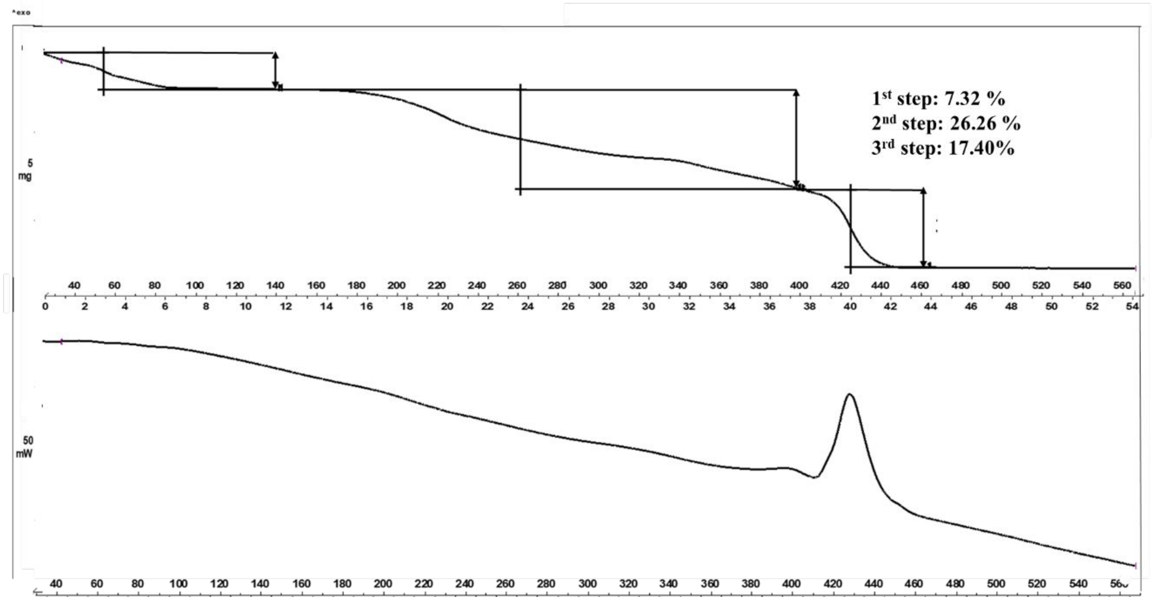

Figure S8. TG curve of $[Co(ox)(en)_2]n[V_2O_6Na(H_2O)]_n{\cdot}H_2O$ (**9**)

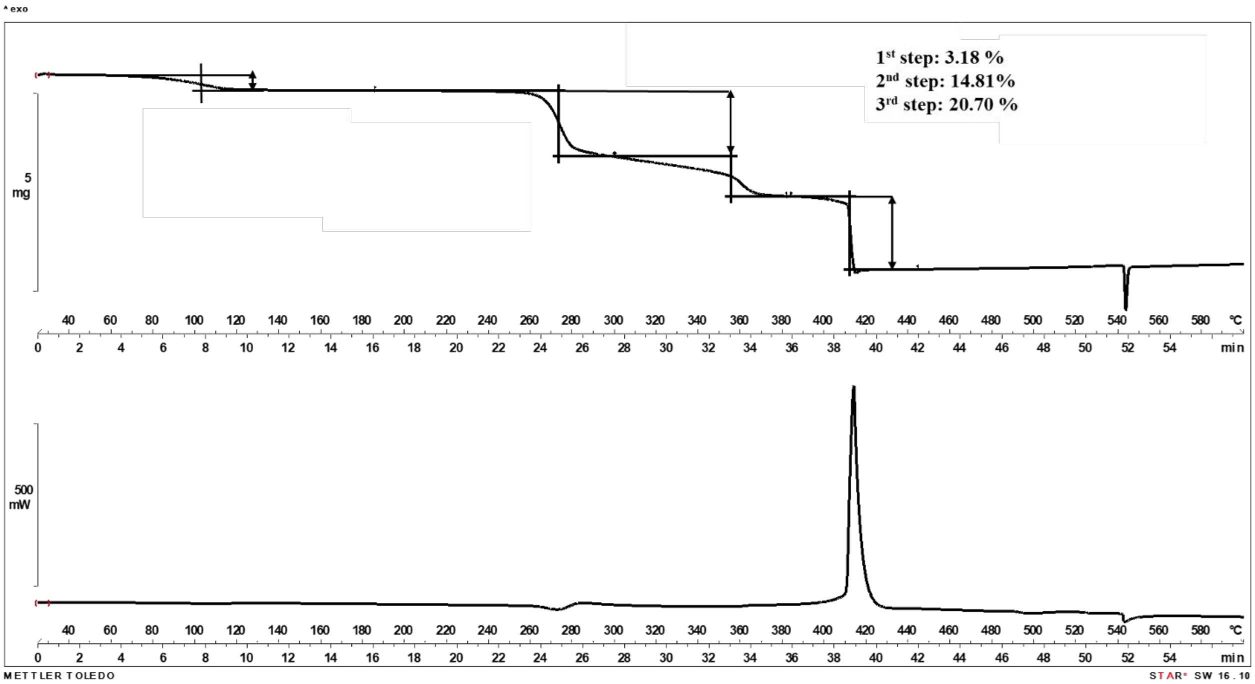

Figure S9. a) TG curve of $[Co(en)_3]_n[V_3O_9]_n{\cdot}nH_2O$ (**10**)-crystals

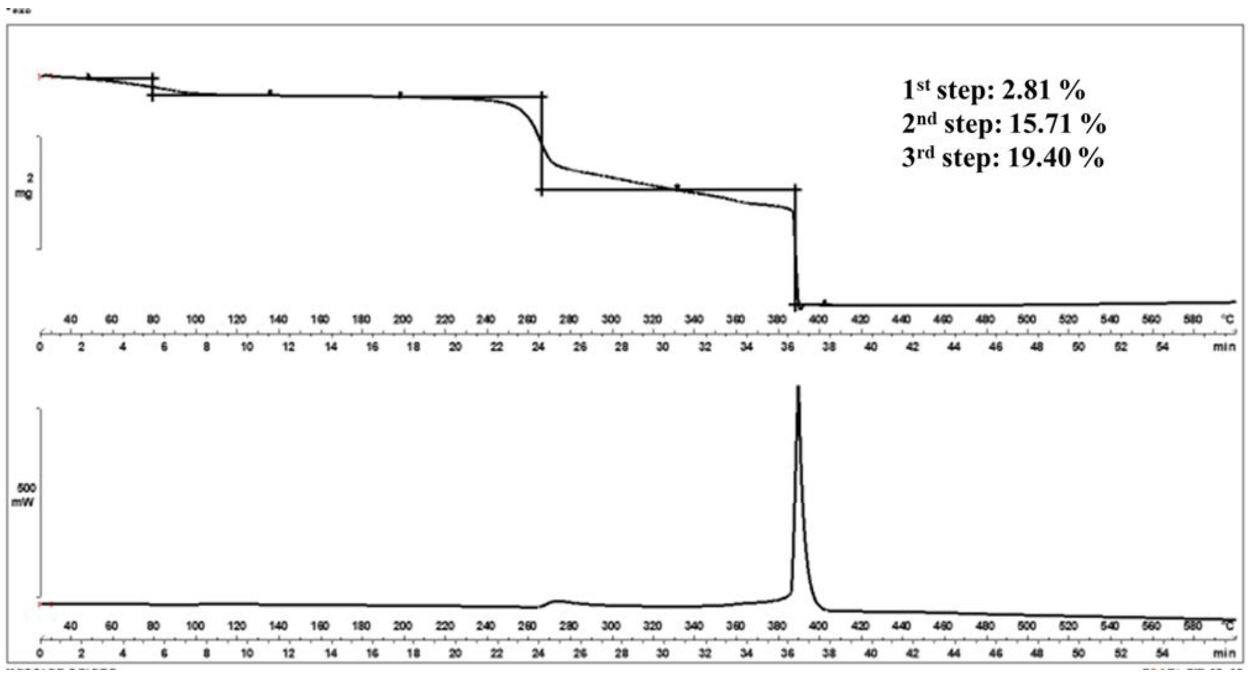

b) TG curve of yellow powder

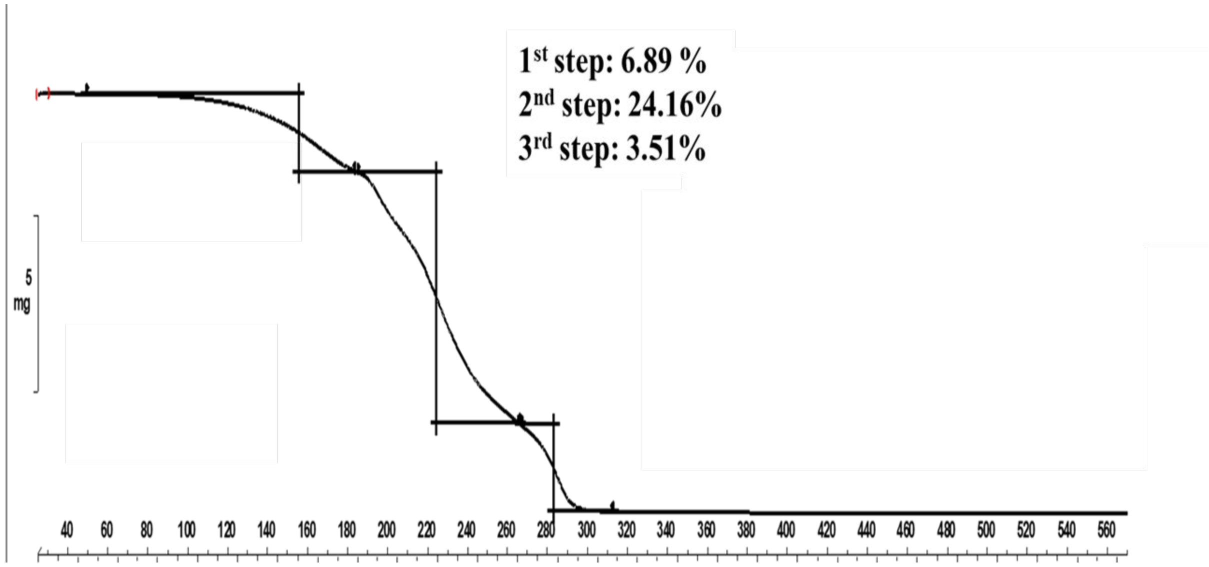

Figure S10. TG curve of $[Co(ox)(NH_3)_4]_2[H_2Mo_8V_5O_{40}Na_2(HO)_8]\cdot 5.5H_2O$ (**11**)

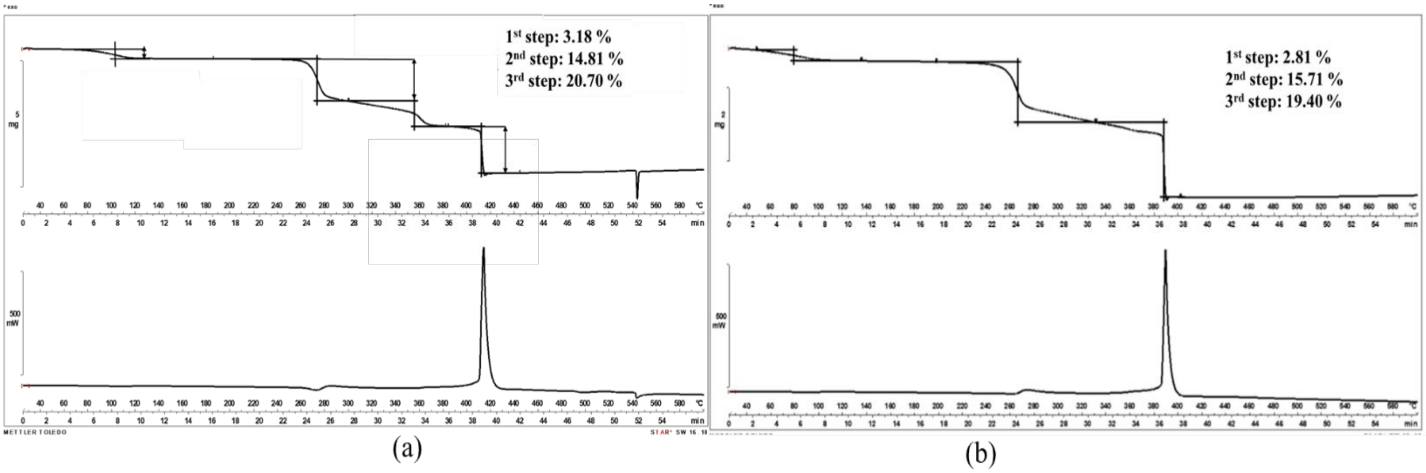

Figure S11. Comparasion of TG of compound **10** (a) and yellow powder (b) obtained in reaction of $[Co(en)_3]Cl_3\cdot H_2O$ and NH_4VO_3 without acid or in same reaction but with addition of $Na_2MoO_4\cdot 2H_2O$

To further study the purity of all crystals, the bulk products were crushed into a fine powder suitable for PXRD analysis. The PXRD patterns confirmed that the bulk powder is a single-phase material. The diffraction peak positions in the experimental PXRD patterns for samples agree well with the simulated patterns. This indicates that the structures of the bulk powders are consistent with those of single crystals, confirming that the phase purity is satisfactory.

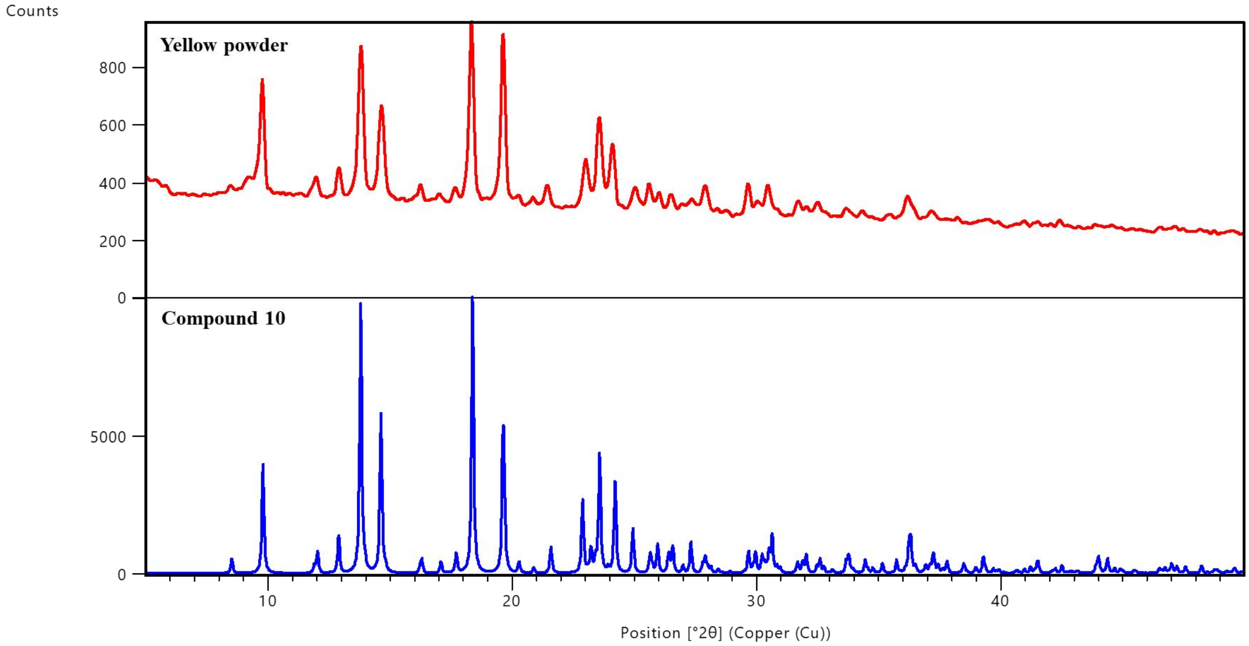

Figure S12. Comparison of PXRD of compound **10** and yellow powder obtained in reaction with $[Co(en)_3]Cl_3$ and NH_4VO_3 without acid

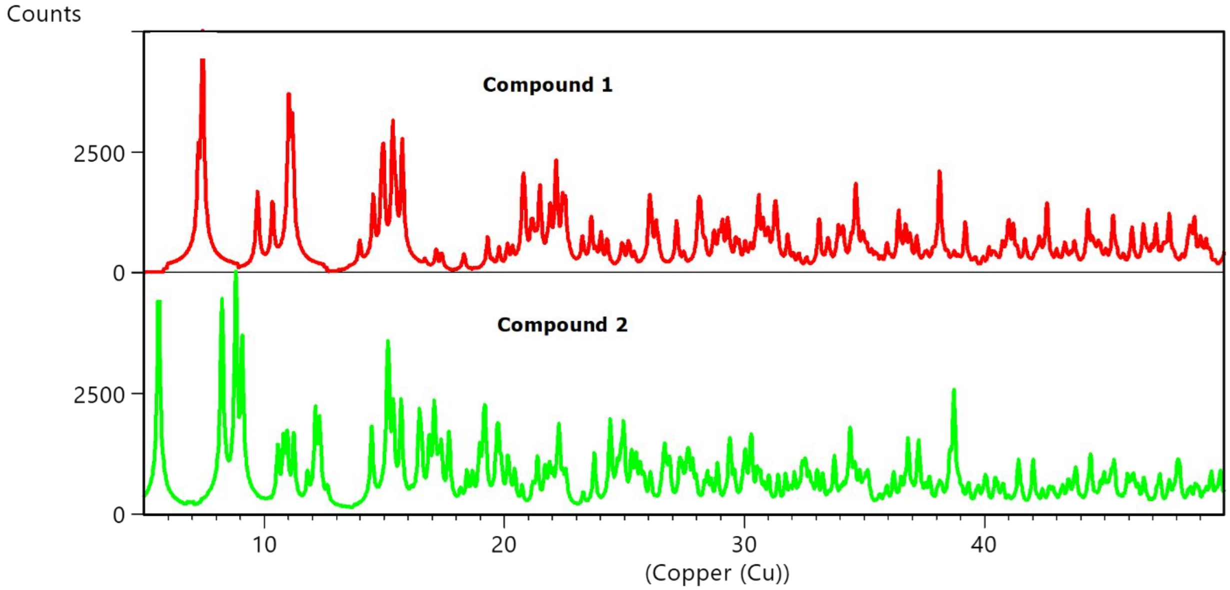

Figure S13. PXRD patterns of **1** and **2**

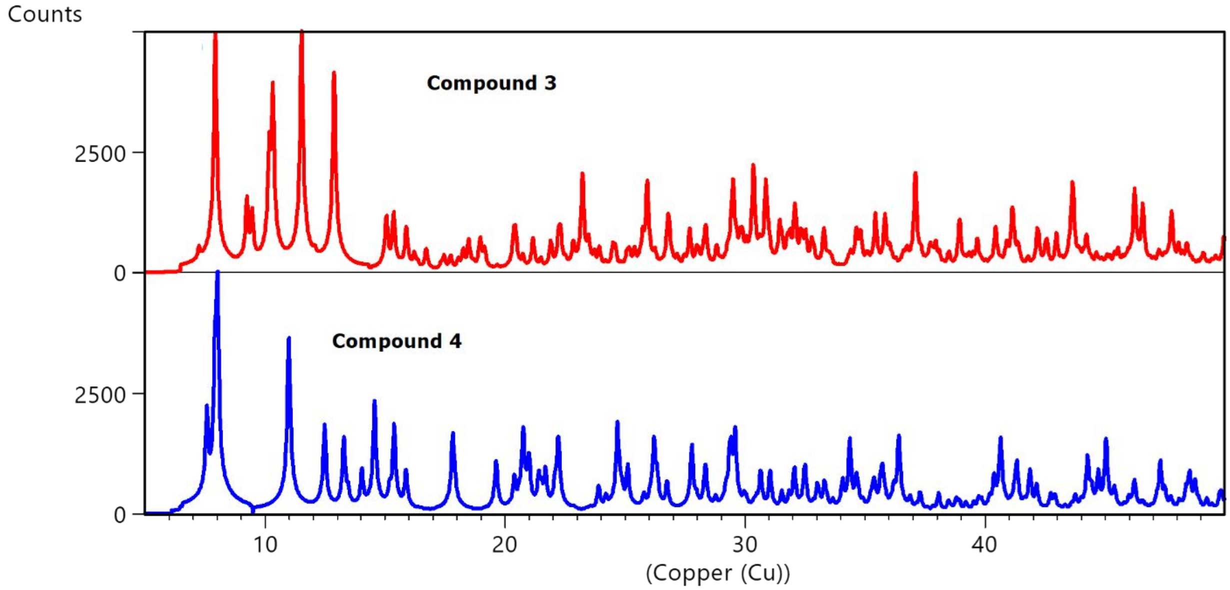

Figure S14. PXRD patterns of **3** and **4**

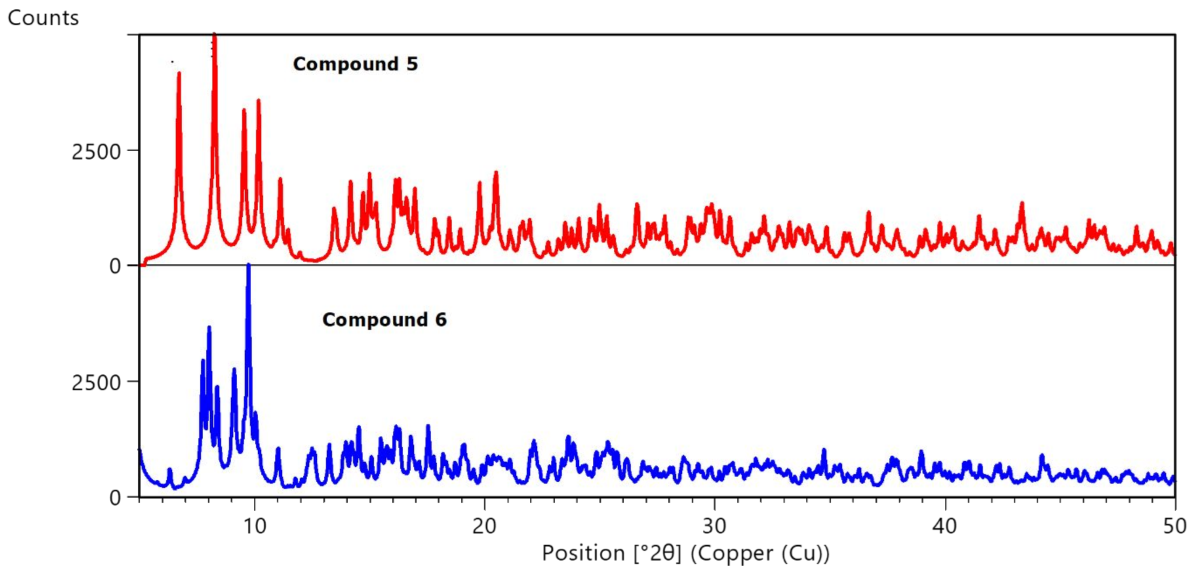

Figure S15. PXRD patterns of **5** and **6**

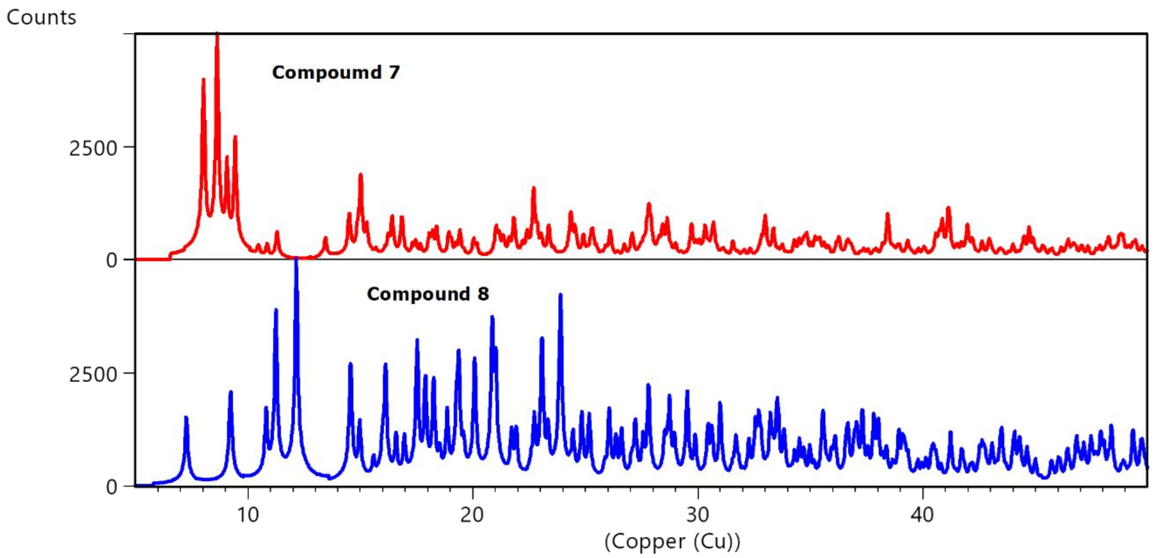

Figure S16. PXRD patterns of **7** and **8**

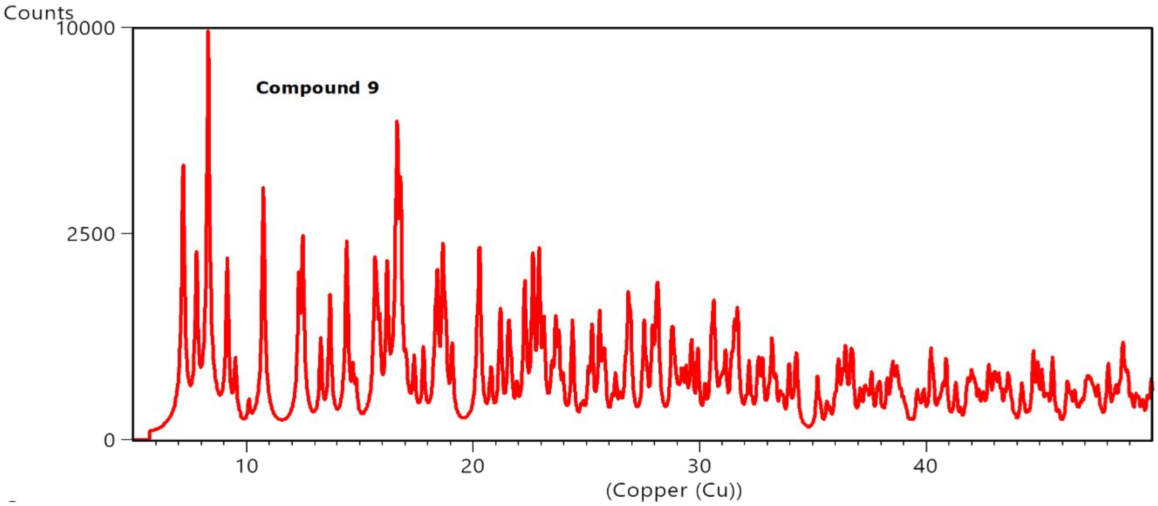

Figure S17. PXRD pattern of **9**

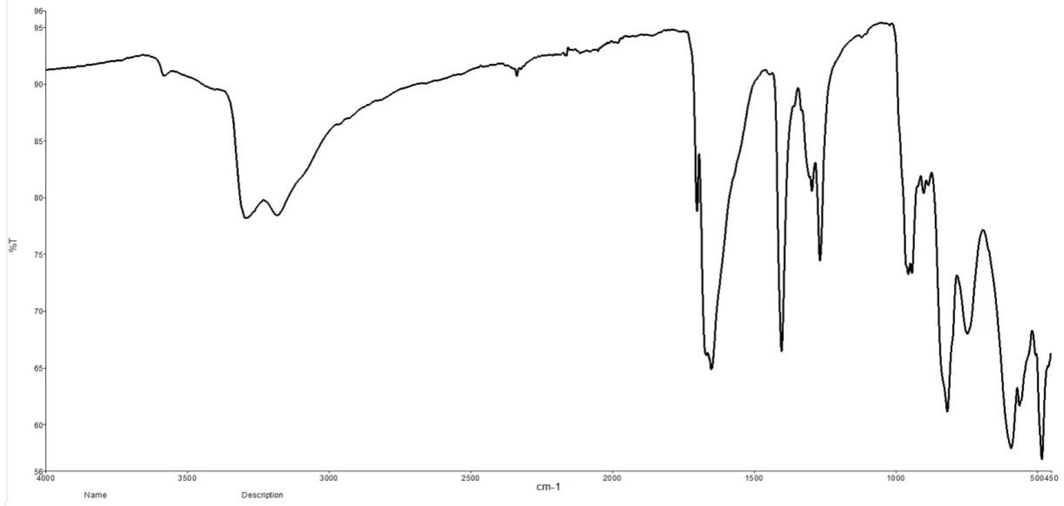

Compound 1

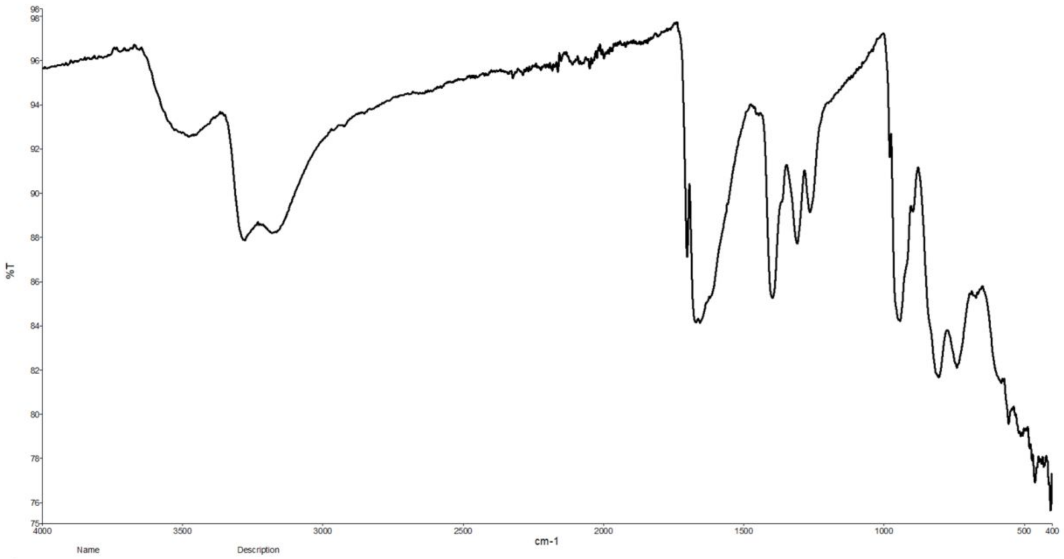

Compound 2

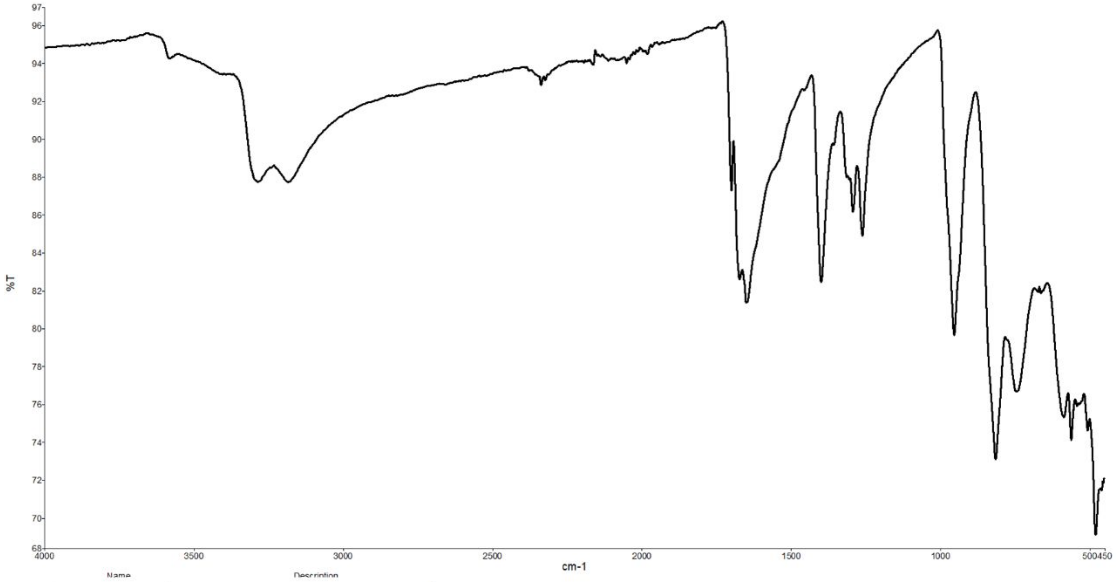

Compound 3

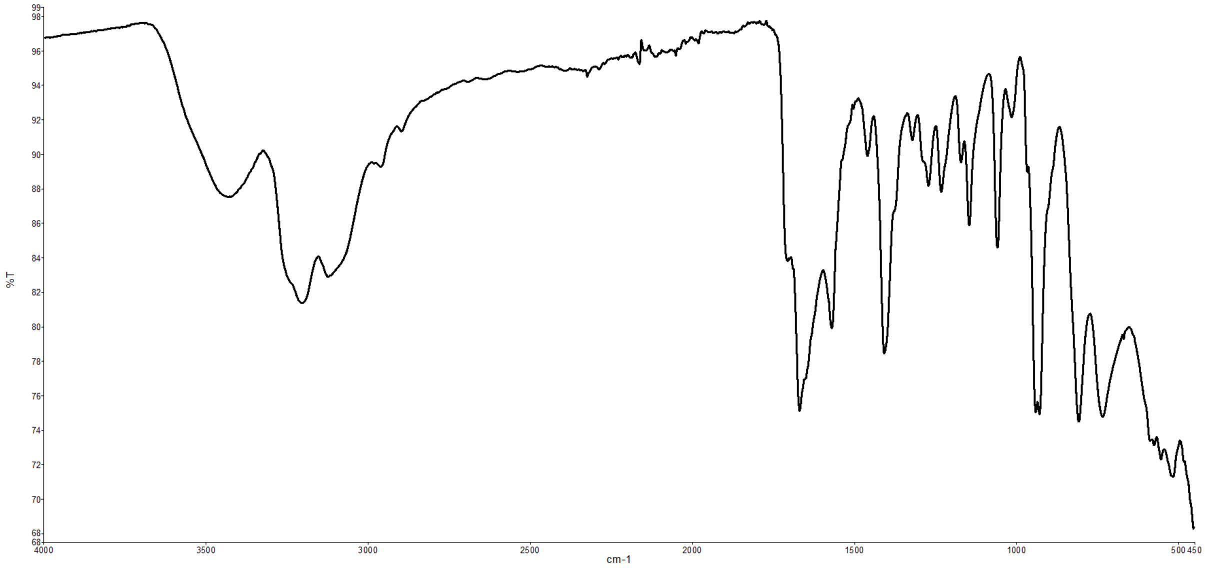

Compound 4

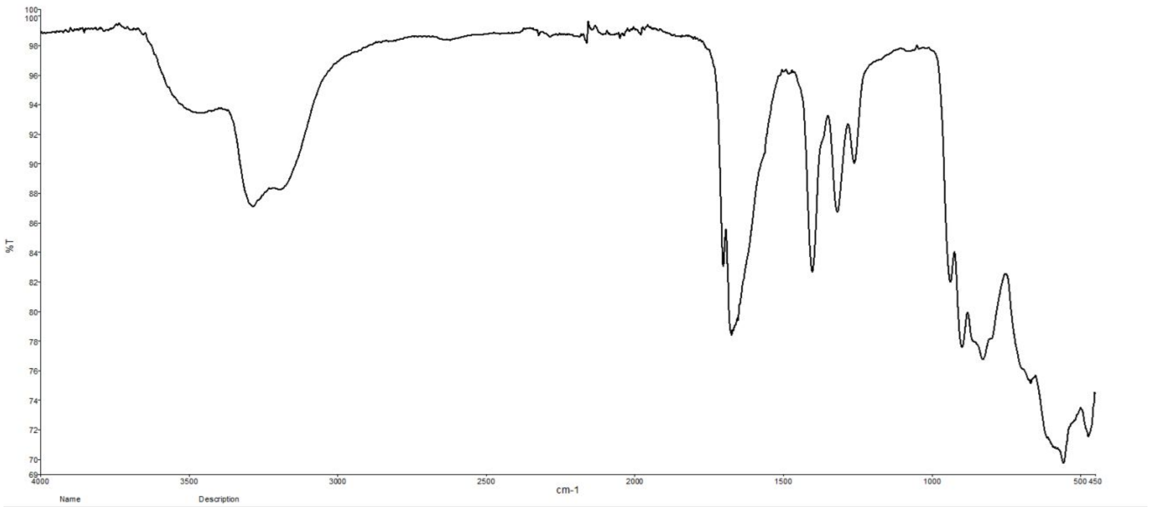

Compound 5

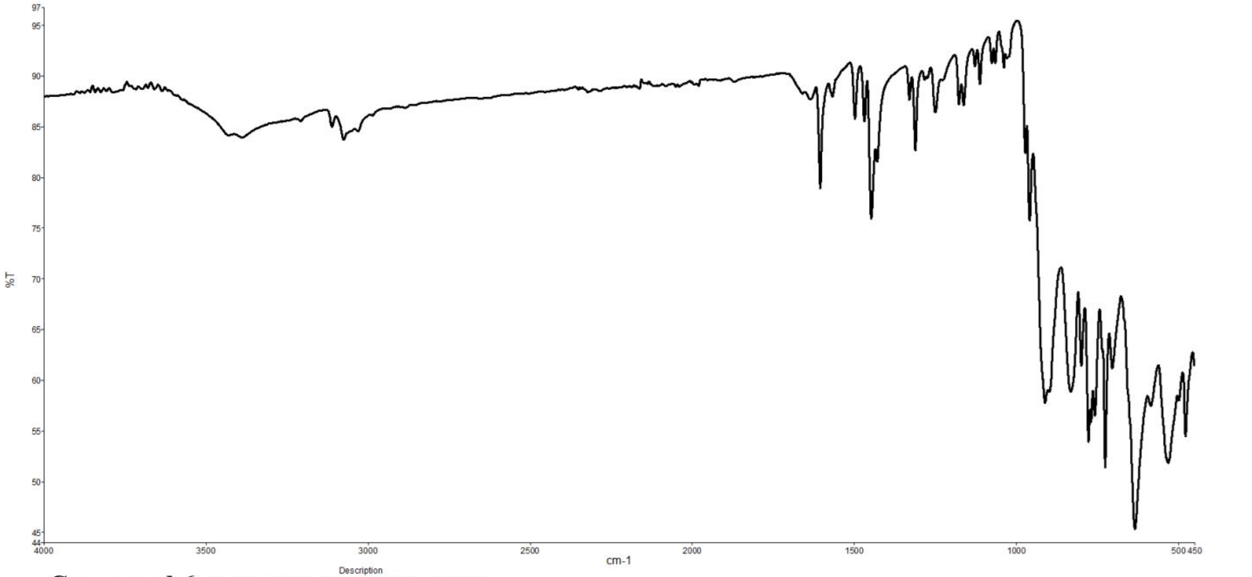

Compound 6

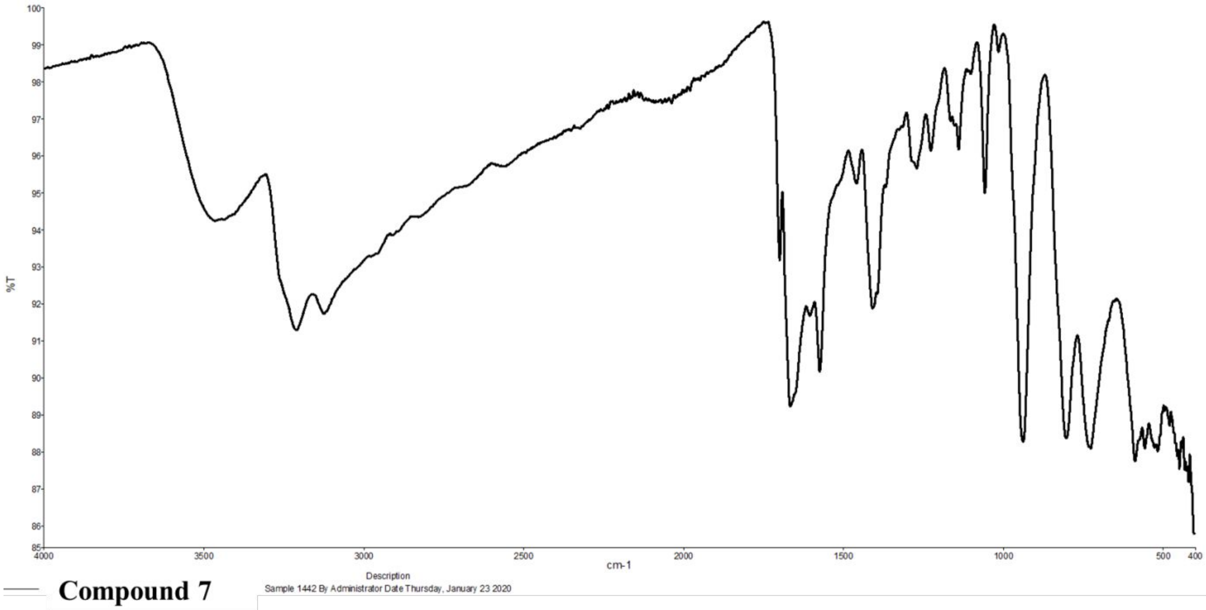

Compound 7

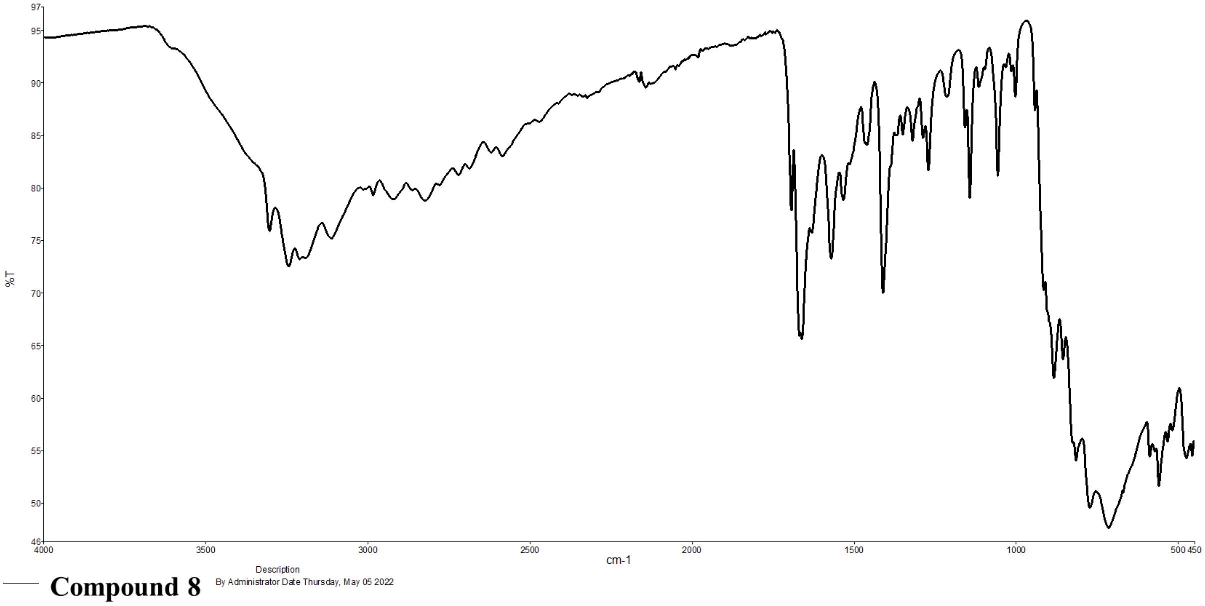

Compound 8

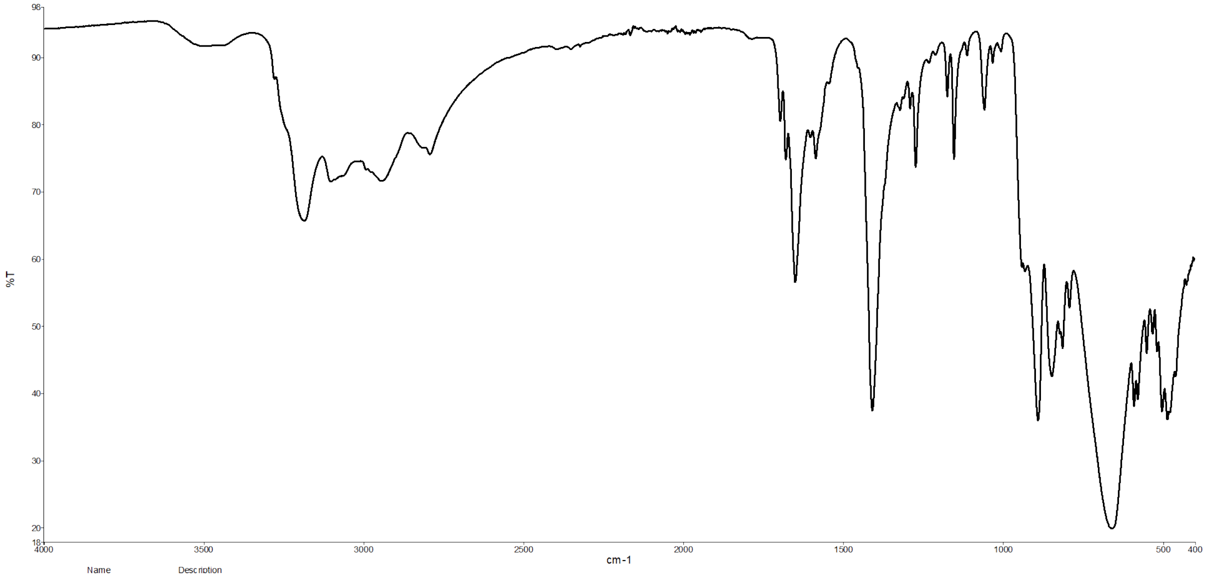

Compound 9

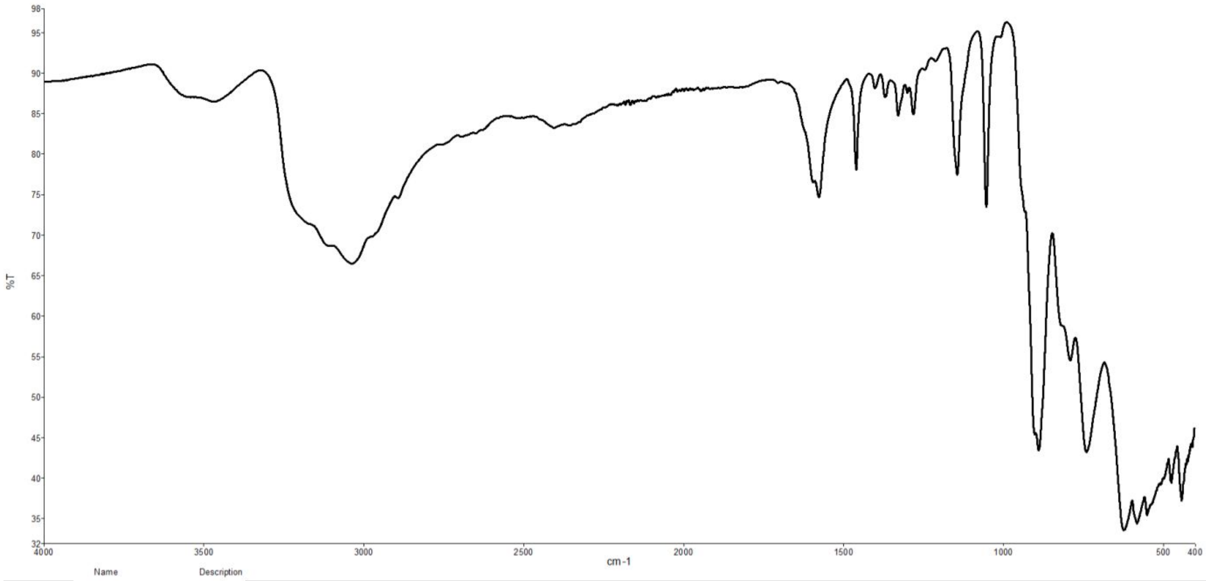

Compound 10

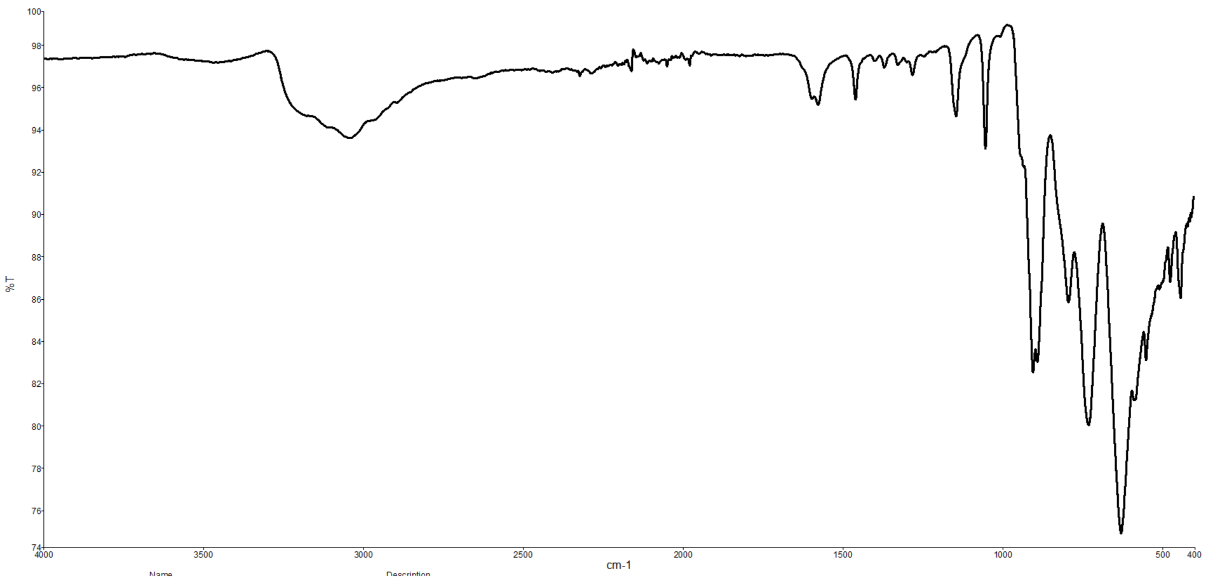

Compound 11

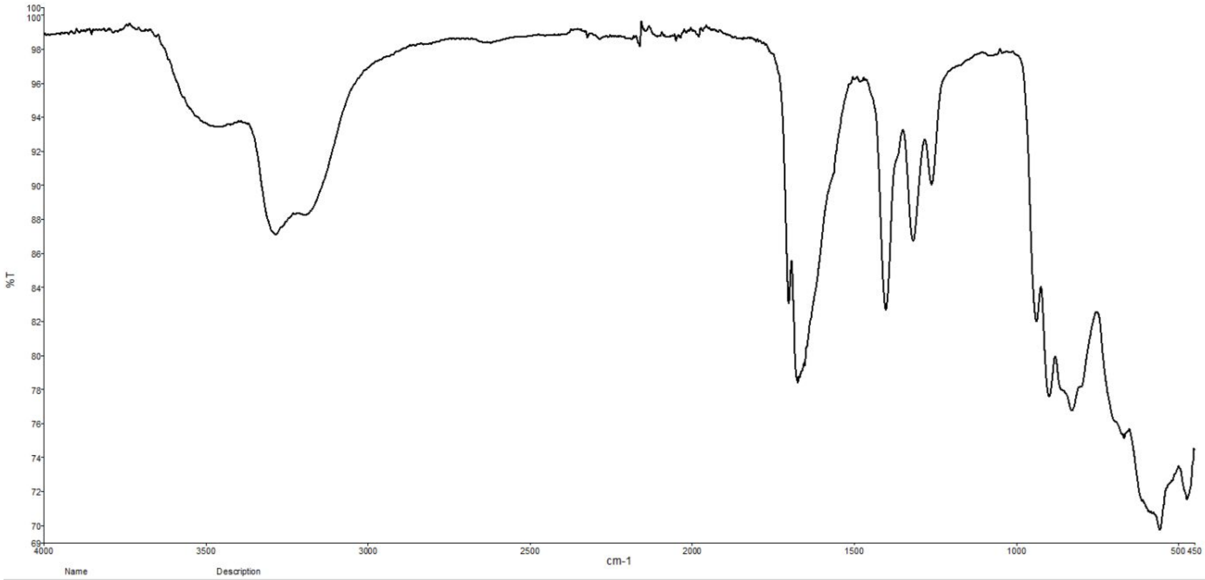

Compound 12

Figure S18. IR spectra of compounds **1-12**

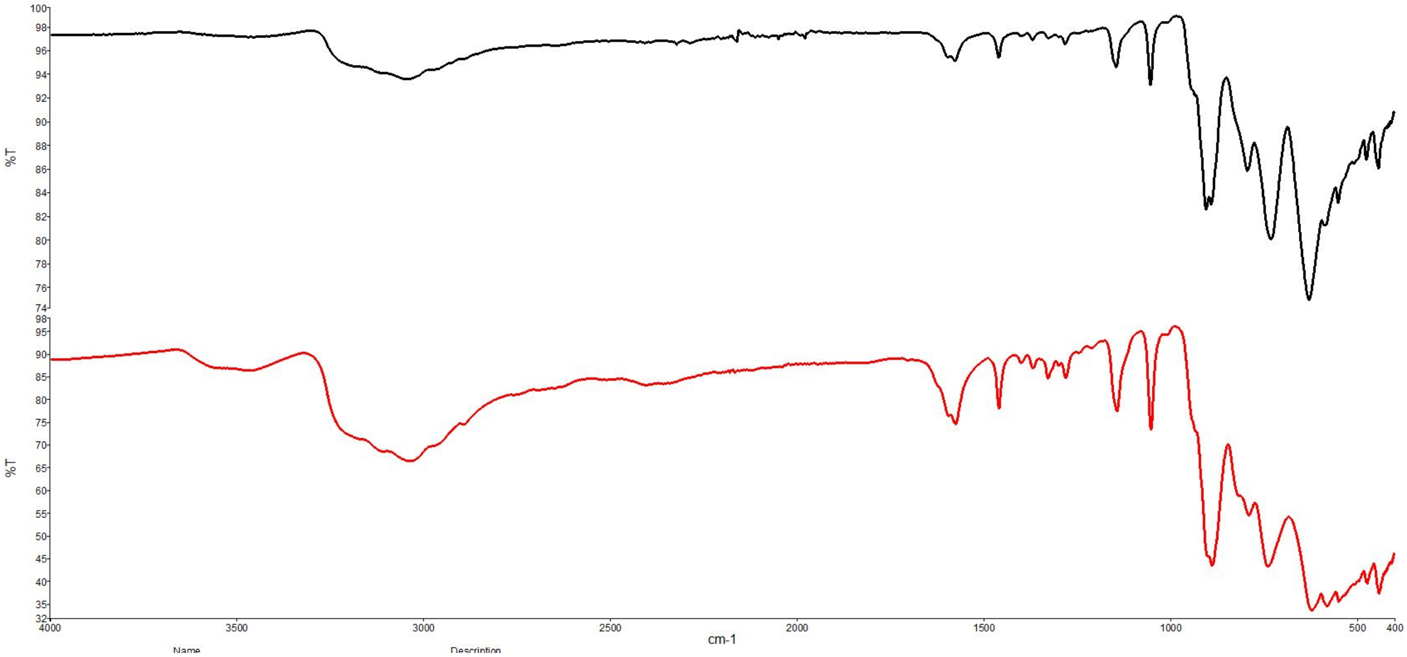

Figure S19. Comparasion of IR spectra of compounds **10** (red) and yellow powder (black)

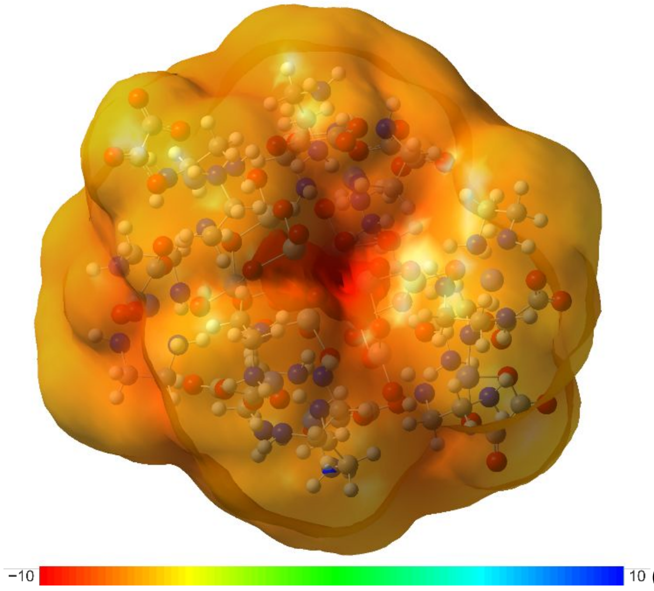

(**5**)

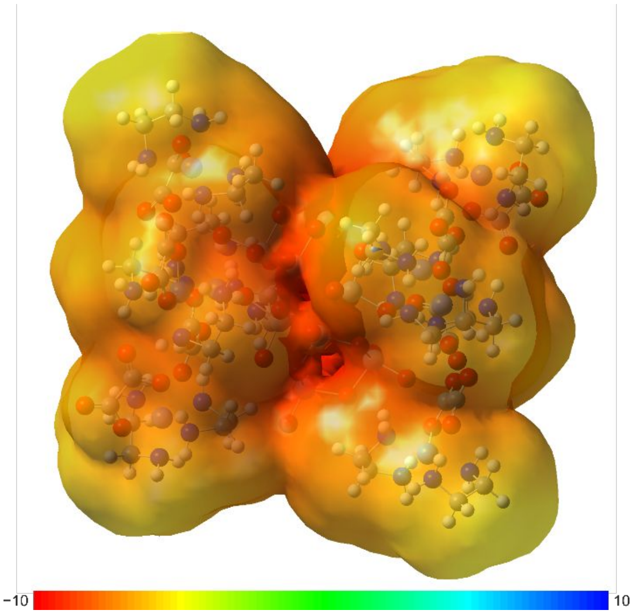

(**6**)

Figure S20. Electrostatic potential mapped on the electron density isosurface (0.001 a.u.) for the optimized structure of $[CoC_2O_4(en)_2]_4[H_2V_{10}O_{28}]\cdot 12H_2O$ (**5**) and $[CoC_2O_4(en)_2]_6[V_{10}O_{28}]\cdot 16H_2O$ (**6**)
